# Constructing a comprehensive disaster resilience index: The case of Italy

**DOI:** 10.1371/journal.pone.0221585

**Published:** 2019-09-16

**Authors:** Sepehr Marzi, Jaroslav Mysiak, Arthur H. Essenfelder, Mattia Amadio, Silvio Giove, Alexander Fekete

**Affiliations:** 1 Centro Euro-Mediterraneo sui Cambiamenti Climatici and Università Ca' Foscari Venezia, via della Libertà, Venice Marghera, Italy; 2 Department of Economics, Università Ca' Foscari Venezia, Cannaregio 873 –Fondamenta San Giobbe, Venice, Italy; 3 Institute of Rescue Engineering and Civil Protection, TH Köln (University of Applied Sciences), Betzdorfer Straße 2, Cologne, Germany; US Army Engineer Research and Development Center, UNITED STATES

## Abstract

Measuring disaster resilience is a key component of successful disaster risk management and climate change adaptation. Quantitative, indicator-based assessments are typically applied to evaluate resilience by combining various indicators of performance into a single composite index. Building upon extensive research on social vulnerability and coping/adaptive capacity, we first develop an original, comprehensive disaster resilience index (CDRI) at municipal level across Italy, to support the implementation of the Sendai Framework for Disaster Risk Reduction 2015–2030. As next, we perform extensive sensitivity and robustness analysis to assess how various methodological choices, especially the normalisation and aggregation methods applied, influence the ensuing rankings. The results show patterns of social vulnerability and resilience with sizeable variability across the northern and southern regions. We propose several statistical methods to allow decision makers to explore the territorial, social and economic disparities, and choose aggregation methods best suitable for the various policy purposes. These methods are based on linear and non-liner normalization approaches combining the OWA and LSP aggregators. Robust resilience rankings are determined by relative dominance across multiple methods. The dominance measures can be used as a decision-making benchmark for climate change adaptation and disaster risk management strategies and plans.

## Introduction and background

Climate-related disasters can affect safety and well-being of communities. In recent years, climate-related risks have increased as a result of changing climate, unplanned urbanization, demographic pressures, land-use and land-cover change, biodiversity loss, and eco-system degradation [1–3]. Reducing climate-related risks and strengthening natural disaster resilience are major societal challenges demanding a better understanding of complex interactions between societies, ecosystems and natural hazards under current and future climates. Strategic measures for monitoring and reporting progress made in disaster risk reduction and enhancing resilience are core elements of disaster risk management and climate change adaptation [4–7].

The UN Sendai Framework for Disaster Risk Reduction 2015–2030 [5] emphasized disaster resilience at all levels through “the implementation of integrated and inclusive economic, structural, legal, social, health, educational, environmental, technological, political and institutional measures” that reduce hazard exposure and vulnerability and strengthen resilience. The Sendai Framework calls for investments for resilience [3,5] and mainstreaming disaster risk reduction into the sustainable development policies [5,8,9].

The EU strategy on adaptation to climate change calls for integrating adaptation actions and disaster risk management policies to promote sustainable growth and disaster resilience at all levels [10]. In 2015, a conference entitled “Building a resilient Europe in a globalized world” was held by the Joint Research Centre (JRC) and the European Political Strategy Centre (EPSC) to explore different aspects of disaster resilience across European institutions. As a result, the Disaster Risk Management Knowledge Centre was launched to strengthen links between science and policy and enhance risk governance in Europe [11,12].

Previous attempts to measure resilience [13–16] addressed it in the form of networked social and economic capacities that comprise attributes of different dimensions such as infrastructures, economy, governance and environment [17–24]. Resilience combines preparedness to hazard strikes, social and economic cohesion and trust for facing disasters and promoting adaptive capacity and sustainability, by considering resource availability and demographic characteristics to deal with the post-disaster era [3,14,25]. Resilience focuses on the quality of life of the people at risk and on developing opportunities to enhance the societal preparedness and restoration processes [26,27]. Cimellaro et al (2010) defines a disaster resilient community as the one which can withstand an extreme event, with a tolerable level of loss, and is able to take (risk) mitigation actions consistent with achieving that level of protection [27]. A detailed background on the resilience concept is given in the S1 Appendix.

Resilience can be measured with respect to a set of components. Cutter et al. (2014, 2010, 2008) classifies resilient components as ecological, social, economic, organizational, infrastructure and community competence pillars [14,28,29]. The resilience of ecological systems can be associated with various factors related to biodiversity, redundancies, response diversity, governance and management policies [30–33]. The social pillar of resilience is influenced by factors related to communications, risk awareness and preparedness which are closely correlated with a community’s demographic characteristics and its access to resources. Post-disaster property loss and the effects of business disruption have been stated as the components of the economic pillar, revealing the operational role of businesses and organizational and institutional entities [34]. Organizational resilience comprises the physical properties of organizations and emergency assets that guarantee and manage a proper response to disasters [35]. The infrastructure pillar includes the characteristics of physical systems as well as the degree of interdependency of the infrastructure construct. Lastly, community competence captures a population’s wellness, quality of life and emotional health, which indicate how a community will perform before and after disaster strike [36]. Recently, Parsons et al. (2016) conducted a research on disaster resilience in Australia, focusing on coping and adaptive capacity as the main dimensions of resilience [15]. Accordingly, social and economic capital, infrastructure and planning, emergency services, community cohesion, remoteness, information, engagement and governance have been considered as the main components of coping and adaptive capacity for assessing disaster resilience in Australia.

Resilience measurement encompasses several stages known as “tiered approach” [37]. Low-level tiers are cost-effective screening assessments of risk reduction actions. Progression to higher tiers occurs while the risk exceeds acceptable thresholds [37,38]. The tier I assessment identifies the major social, ecological, economic and technological features of the system and is often based on indicator assessments or surveys [29,37,39]. The Tier II assessment entails a dynamic model of the system, and describes the relationships of its components over time and space. It employs participatory multi-criteria decision analysis tools such as Resilience Matrix (stakeholder-driven approach) [40]. Tier III evaluates the interactions among the system's components, along with an impact assessment [41]. The outcomes offer a range of potential performance for several possible futures [37,42,43]. Collective resilience (at national, regional, provincial and municipal scales) is assessed mostly by means of quantitative indicators and composite indices (Tier I approach) [15,25,40].

Indicator-based assessments are widely used to assess the relative resilience of geographic units by aggregating separate indicators into one composite index [16]. Place-based Composite resilience indices can capture a snapshot of the most important facets involved in promoting resilience [14]. Baseline resilience indicators for communities (BRIC), disaster resilience of place (DROP), community disaster resilience index (CDRI) and Foster’s resilience capacity index (RCI) could be mentioned as the most familiar resilience indices throughout the literature, in assessing resilience at the provincial administrative level, and have been used as a basis to build upon by various scholars and international agencies [14,16,28,44,45]. Despite the fact that Italy is highly exposed to natural hazards, very few studies at the Italian scale focus on disaster resilience indices. Recently, Graziano and Rizzi (2016) have explored the resilience of the local systems for Italian provinces by using an indicator-based assessment following the theoretical frameworks conducted by Dallara and Rizzi (2012), Graziano and Provenzano (2014) and Rizzi and Graziano (2013) [46–49]. It has been stated that to reach more robust resilience assessments, multi-scalar measurements, including various collective levels (e.g. regional, provincial and municipal levels), are preferable [13,50,51]. Marzi et al. (2018) argues that if a composite index is estimated only at a higher administrative or statistical level, the inherent variability of performance at lower administrative levels will be neglected [52]. In addition, Hinkel (2011) suggested that the indicator-based assessments are appropriate at local scale and when systems can be narrowly defined [53]. Hence, the variability of resilience measures at lower scales (e.g. municipal level) should be considered in the decision-making process to avoid inadequately informed policies [52]. At the municipal administrative level, most of the indicator-based assessments targeted social vulnerability instead of resilience, including only socioeconomic and demographic features of resilience [54–57]. Some coping and adaptive capacity elements, such as distance-based accessibility measures, as well as infrastructure and economic resource variables, are excluded from the aforesaid indices, which are considered as the core elements of disaster resilience.

In this paper, we propose an innovative composite disaster resilience index (CDRI) at the municipal level for the whole of Italy, that builds upon research on social vulnerability conducted by Italian National Statistical Office (ISTAT) [58]. Subsequently, we perform an extensive sensitivity analysis to explore the influence of methodological choices (such as the choice of normalization and aggregation methods) and assumptions on the ensuing results. Our framework embraces features from both Tier I and Tier III approaches. The work developed in this article is structured as follows: Section 2 explains the methodological framework, the data preparation and the multivariate analysis performed to narrow down the choice of indicators for the composite index. Section 3 describes the aggregate results at the municipal scale and presents the outcomes of sensitivity and robustness analysis. Section 4 discusses the results and section 5 concludes with the main findings.

## Data and methodology

### Conceptual framework and indicators used

The framework for developing the Composite Disaster Resilience Index (CDRI) is inspired by Cutter et al. (2014, 2008) [14,28] and Parsons et al. (2016) [15], and comprises services, cohesion, economic resources, housing conditions, education, environmental status and institutions. The framework combines indicators for social vulnerability and additional ones describing accessibility, environment and institutions. The choice of underlying indicators has been driven by an extensive literature review, and is motivated below:

#### Access to services

Accessibility (or remoteness) can be interpreted both in terms of coping and adaptive capacity. Distance-decay accessibility (travel time and distance) to emergency services such as hospitals, fire & rescue stations has been considered also in previous studies [59–64]. Accessibility can also be embedded in the context of adaptive capacity and sustainable development. Access to health, education services and other assets plays a crucial role in reducing inequalities and climate resilient pathways [65–67]. In recent resilience discourse, access to information and communication technologies (ICT) has been considered a vital aspect of the adaptive cycle needed to cope with emerging threats such as climate change [68]. According to Bellini & Nesi (2018), smart technologies such as Internet of Everything (IoE) and Knowledge-Information-Data (KID) are critical resources to develop adaptive capacity components [68].

In Italy, accessibility to essential services such as education, health and mobility is a defining feature of disadvantaged (also called inner) areas [69]. In our analysis, we use two distance decay indicators to service centres, and fire and rescue units. Following the methodology employed in the Strategy for economically disadvantaged (inner) areas [69,70], these indicators are not weighted by population served by service. Figure C in S6 Appendix shows that this has no, or only minor, effect on the results of our analysis.

#### Institutions

High institutional quality and governance can ensure effective implementation of emegency planning, as well as climate change adaptation and resilience policies [28,66,71,72]. Accountability of and trust in institutions and officials is an important element of organizational resilience [28,73,74]. According to Larsen (2014), a well-functioning democracy is positively correlated with the level of social trust in the system [74]. Hooghe and Stiers (2016) argue that participation in elections as a representative element of democracy increases social and political trust regardless of who wins or loses in an election [75]. In the case of Italy, despite past diffidence, recent trends show that participation generates trust and, as a consequence, confidence in institutions is increasing significantly among the population showing higher participation rates [76]. In our study, we consider participation rates in elections as a proxy to evaluate trust in institutions.

According to the World Governance Indicator (WGI) proposed by [77] in the context of the Knowledge for Change Programme promoted by World Bank, election participation and endowment of social, economic and health facilities (translated into accessibility indicators in our study) are considered as the main constituents of the “voice and accountability” and “government effectiveness” criteria of Governance [78]. We have combined them in a single dimension: “access to services and quality of institutions”.

#### Housing conditions

Housing conditions and dwellings are referred to as infrastructure [14,15,25,28]. The quality and occupancy rate of dwellings can affect the degree of physical damage and vulnerability of the residents in time of disaster shock [55,60,79,80]. Hence, empowering the elements regarding the housing and dwellings can promote coping capacity and consequently resilience.

#### Cohesion

Cohesion increases the ability of communities to ‘bounce back’ in the aftermath of a disaster strike [81,82]. Cohesion refers to a “bond that keeps societies integrated” [74]. Cohesion comprises economic and social factors such as inclusion, membership and participation in society. Factors driving disparities reduce cohesion and consequently resilience. Cohesion may comprise demographic elements of disparity, dependencies, turnover and commuting rates [25,83–85]. We considered family structure, age dependencies, gender equality and commuting as the indicators for cohesion.

#### Education

Level of education is often used as a proxy degree of preparedness for dealing with shocks and reinforces responses [3,15,25,85,86]. Higher education levels have been considered as elements of adaptive capacity that can affect the productivity yields in R&D and innovation sectors [87–91].

#### Economic resources

Economic resources play an important role in boosting resilience and adaptive capacity [66,72,92]. Per capita income, income distribution, poverty rates and unemployment have been employed to assess economic resources [71,90,91,93,94]. In our study, we also considered land valuation, which can support emergency response, recovery and reconstruction after disaster shock [16,95,96].

#### Environment

Environmental and ecosystem aspects of resilience have been embedded in the ecological/ecosystem dimension in previous studies [14,28]. According to an IPCC report, conservation of protected areas and ecological corridors can be important for ecosystem-based climate adaptation and disaster risk reduction strategies [4]. Expansion and conservation of protected areas and ecological corridors leads to preserving ecosystem services and ecological resilience, which are the core elements of green infrastructure planning in Europe [97,98].

Table 1 shows the initial set of resilience indicators classified at individual, household and community levels. A detailed explanation of the sub-indicators can be found in the S2 Appendix.

**Table 1:** Full list of disaster resilience indicators considered for the analysis.

Category	Sub-Category	Code	Indicators	Unit	Source	Year	sub-scale	Impact on Resilience
**Access to Services and quality of institutions**	Public infrastructures and Trust in Government and authorities	ACC_1	Distance and travel time to service centers	Meters-Minutes	Inner Areas-ISTAT-Manual	2012	community level	decrease
		ACC_2	Distance and travel time to fire brigades	Meters- Minutes	Dipartimento dei Vigili del Fuocco -Manual	2009	community level	decrease
		INS_1	Election participation	%	Ministero dell'Interno	2016–2017	community level	increase
**Housing Conditions**	Housing condition and population density	HC_1	Quality rate of dwellings	%	ISTAT-Census	2011	community level	increase
		HC_2	Rate of empty dwellings over total	%	ISTAT-Census	2011	community level	increase
		HC_3	Index of overcrowded residences	%	ISTAT-8Mila	2011	household level	decrease
		HC_4	Residential buildings over total	%	ISTAT-Census	2011	community level	decrease
**Cohesion**	Family structure	COH_1	Index of single parent families	%	ISTAT-Census	2011	household level	decrease
		COH_2	Index of large families	%	ISTAT-Census	2011	household level	decrease
		COH_3	Index of small families	%	ISTAT-Census	2011	household level	decrease
	Dependencies	COH_4	Index of elderly dependence	%	ISTAT-8Mila	2011	individual	decrease
		COH_5	Old age index	%	ISTAT-8Mila	2011	individual	decrease
		COH_6	Index of minor dependence	%	ISTAT-8Mila	2011	individual	decrease
		COH_7	Share of the families with assistance need	%	ISTAT-8Mila	2011	household level	decrease
		COH_8	Participation in the labor market—female	%	ISTAT-8Mila	2011	individual	increase
	Commuters	COH_9	Commuting rate for study or work	%	ISTAT-8Mila	2011	individual	decrease
		COH_10	Containment index	%	ISTAT-Census	2011	individual	increase
		COH_11	Attraction index	%	ISTAT-Census	2011	individual	decrease
**Education**	Education	EDU_1	Illiteracy	%	ISTAT-8Mila	2011	individual	decrease
		EDU_2	Low education index	%	ISTAT-Census	2011	individual	decrease
		EDU_3	High education index	%	ISTAT-Census	2011	individual	increase
**Environment**	Environmental status/ecosystem protection	ENV_1	Share of the protected lands	%	Natura 2000 Network	2017	community level	increase
		ENV_2	Share of ecological corridors	%	Copernicus-Manual	2017	community level	increase
**Economic Resources**	Economic capacity and distribution	RE_1	Income	Euros	Ministry of finance	2011	individual	increase
		RE_2	GINI index	GINI	Manual	2011	community level	decrease
		RE_3	Unemployment rate	%	ISTAT-Census	2011	community level	decrease
		RE_4	Cadastral stock (property value)	1000 Euros	Agenzia Entrate	2013	community level	decrease
		RE_5	Share of the families with potential economic hardship	%	ISTAT-8Mila	2011	household level	decrease

### Data used

Data was collected from multiple sources, while the main data source was the national 2011 Italian census [99]. Another important data source was the 8milacensus database [100], comprising 99 indicators arranged in historical series from 1951 to 2011. Income data was obtained from the Department of Finance (2018) [101] and was used to calculate inequality in income distribution according to the GINI coefficient. We used the GiniWegNeg R package [102] that makes it possible to estimate Gini-based coefficients for cases that also include negative incomes. Land values were estimated as cadastral stock and obtained from the Agenzia Entrate database (2013) [103] at the municipal level and covering the entire Italian territory.

The distances between municipalities centroids is measured by using the TomTom MultiNet road network (2013). The travel time and the total number of commuters travelling between municipalities have been computed *from* (or *to*) a municipality by aggregating the *origin* (or at the *destination*) municipality code based upon the commuting matrices for all Italian municipalities provided by Italian National Statistical Office (ISTAT) [104]. A similar methodology has been used to estimate the matrix of total travelling time and total number of commuters travelling between municipalities and service centres but filtering the destination municipality codes that are service centres. Service centres are defined as municipalities that have: a) a full range of secondary schools; b) at least one first level DEA hospital, and; c) at least one “silver-type” railway station. Data on municipalities hosting essential services were obtained from Barca et al., 2014. The total number of commuters travelling between municipalities has been used to compute the attraction and containment indices (see S2 Appendix for a detailed description of the computation of these indices). We applied an analogous procedure to estimate distance and travel time to fire stations and rescue service units. The locations of fire stations have been obtained from Dipartimento dei Vigili del Fuoco (2009) [105].

The share of protected lands from the total area was estimated on the basis of the extension of the Special Protection Areas (SPA) and the Sites of Community Importance (SCIs) under the Natura 2000 Network [106,107]. For the ecological corridors, we used the database developed by the European Environment Agency in the framework of the EU Copernicus programme [108]. The database contains Green Linear Elements (GLE) and structural landscape elements which act as important dispersion vectors of biodiversity.

The missing data was less than 5 percent of the overall sample size (59 out of 8092 municipalities encompassed missing values). Hence, we employed the case deletion method suggested by OECD (2008). We have identified outliers based on *skewness-kurtosis* measures [109–113]. Outliers lead to heavy-tailed distributions and may distort basic descriptive statistics such as mean, standard deviation and correlation [109,114,115]. Recent studies consider indicators with absolute skewness greater than 2.25 and kurtosis greater than 3.5 as problematic [115]. The descriptive statistics can be found in S3 Appendix. Some of the indicators (listed in Table A in S3 Appendix) did not meet the skewness-kurtosis criterion and have been transformed by means of Box-Cox transformation. Transformation procedures are widely used in the literature and employed to construct the most often cited global indices such as the Environmental Performance Index (EPI) and the EU Regional competitiveness Index (RCI) conducted by Yale University (2016) and the European Commission (2017), respectively. We adopted the Box-Cox transformation to adjust for outliers, in the same way as in Annoni et al. (2017), to construct the EU Regional Competitiveness Index (RCI). Multicollinearity of the data was assessed to avoid too high intercorrelations. When multicollinearity exceeds a certain threshold, standard errors and variances are inflated, possibly biasing the overall results [109,116]. After performing the multicollinearity test, travel time indicators (ACC1_TT and ACC2_TT), old age index (COH_5) and containment index (COH_10) were excluded from the analysis. The detailed description of the Box-Cox transformation and multicollinearity test and their results can be found in the S3 Appendix.

### Analysis

The selected indicators have been normalized to make them comparable to each other [109]. In order to analyse how different normalization procedures can affect the final results, we evaluated three types of normalization methods, namely Adjusted Mazziotta-Pareto (AMP), Topsis, and z-scores standardization. Since the AMP normalization technique has been used in the social vulnerability index provided by ISTAT, it is considered as the baseline in our analysis.

In order to compare the results with the social vulnerability index, we first construct the resilience index by using the AMP method. AMP is a hybrid, non-compensatory aggregation method penalising the compensability among indicators in order to incorporate for possible trade-offs. The compensability (or compensation degree) denotes trade-offs between higher performance in some indicators and lower performance in other ones. Additive aggregators with high degree of compensation may discard significant underperformance in one or more indicators. For that reason, the compensability should be controlled and the choice of an aggregator and the degree of compensation should be made through expert judgement elicitation, taking into account the context and scope of the analysis. In the AMPI, the penalization is addressed by subtracting a component (*cv_i_*) from a non-weighted arithmetic mean [117]. However, by using AMPI, the degree of penalization is not explicit and trade-offs among the indicators cannot be clearly portrayed in terms of degree of compensation. To unequivocally display the trade-offs with respect to compensability, a spectrum of hybrid methods can be deployed, such as Fuzzy Gamma, Mean-Min function, and generalized mean, among others. Since, we are simultaneously incorporating various normalization procedures as part of the sensitivity analysis, the aggregation must be independent from the type of normalization.

To control the trade-offs during the aggregation process of the indicators, we applied the ordered weighted average (OWA) operator introduced by Yager (1988) which provides a circumstance in which the degree of compensation can be adjusted and modified [118]. The OWA operator provides a family of operators, including a maximum (1,0, 0,…,0), minimum (0,0,…,1), k-order statistics (kth weight equal to 1 and the rest zero), the arithmetic mean ($\frac{1}{n}$, $\frac{1}{n}$…,$\frac{1}{n}$) and a window type OWA, which takes the average of m components in the center [119,120]. The weights can be ordered in different ways and distributed, by using either linear or uniform patterns, as graphically depicted in Figure B in S3 Appendix [121,122]. In order to evaluate how different weights distributions can affect OWA, different combinations of weights have been simulated, following either a linear or uniform distribution. In total, 128 different weights combinations have been tested, 65 of which follow a linear function distribution, while the remaining 63 follow uniform weight distributions patterns.

In order to examine the trade-offs, Yager (1988) introduced the degree of ORNESS determining the proximity to the maximum operator for a particular set of weights [120,123]. The ORNESS index evaluates the extent to which the indicators compensate each other. The ORNESS equal to 1 shows the highest proximity to a maximum operator indicating full compensative trade-offs (optimistic approach). Contrarily, ORNESS equal to zero indicates the highest propensity to a minimum operator reflecting perfect complementary behaviour (pessimistic approach). The special case of ORNESS equal to 0.5 determines the highest proximity to an average (arithmetic mean) operator (additive approach) [124]. The ANDNESS index is a complement of the ORNESS (*ANDNESS* + *ORNESS* = 1), measuring the level of complementarity among the indicators [124–126]. The OWA operator controls the level of compensation by using a different order of weights. The order of weights corresponding to higher ORNESS levels indicates a higher degree of compensation and proximity to a maximum operator and vice versa.

We used the 128 different weight OWA combinations to perform sensitivity-robustness analysis on the CDRI for each of the three different normalization methods (i.e. AMP, Topsis, and z-score). The normalization methods are applied using various combinations of OWA weights (both linear and uniform distributions) reflecting the ORNESS in the range of [0,1]. For sensitivity analysis we also consider the original (not Box-Cox transformed) data. We employ the relative dominance measure (*ρ*) developed by Pinar et al. (2014) to identify the extent of relative dominance of the *i*th administrative unit across simulations [124]. The *ρ* measure takes into account the relationship between administrative units across the simulated combinations to explore to what extent each unit either dominates or is being dominated by other units, by taking into account the overall variability of the results. A detailed description of the normalization techniques, OWA, ORNESS, sensitivity-robustness analysis, and dominance analysis can be found in the S3 Appendix.

## Results and discussion

### Resilience at municipal scale

**Fig 1:**
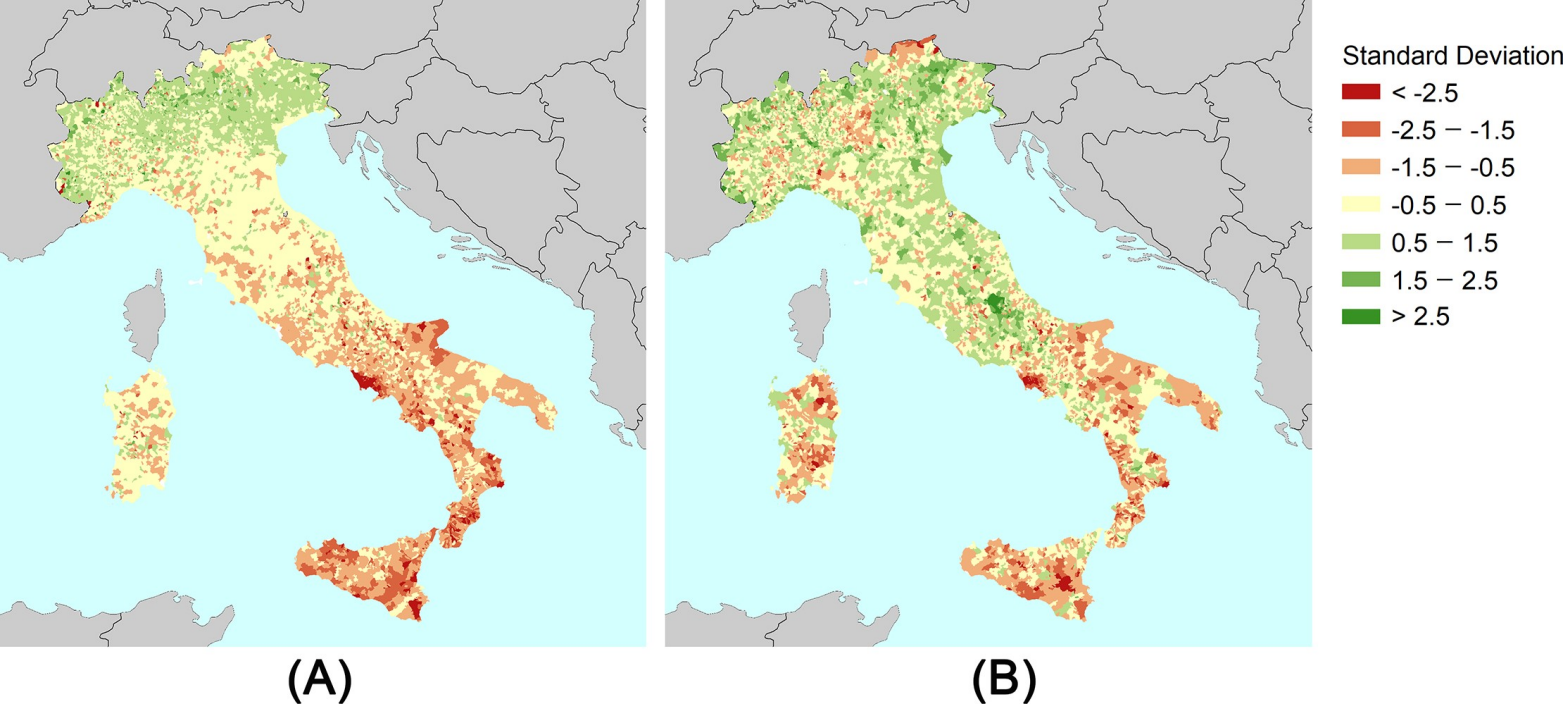
Comparisons between SVI from ISTAT and CDRI derived from AMP analysis. (A) SVI from ISTAT. (B) CDRI. SVI results are inverted (i.e. opposite signal) to facilitate the visual comparison between the results.

**Fig 2:**
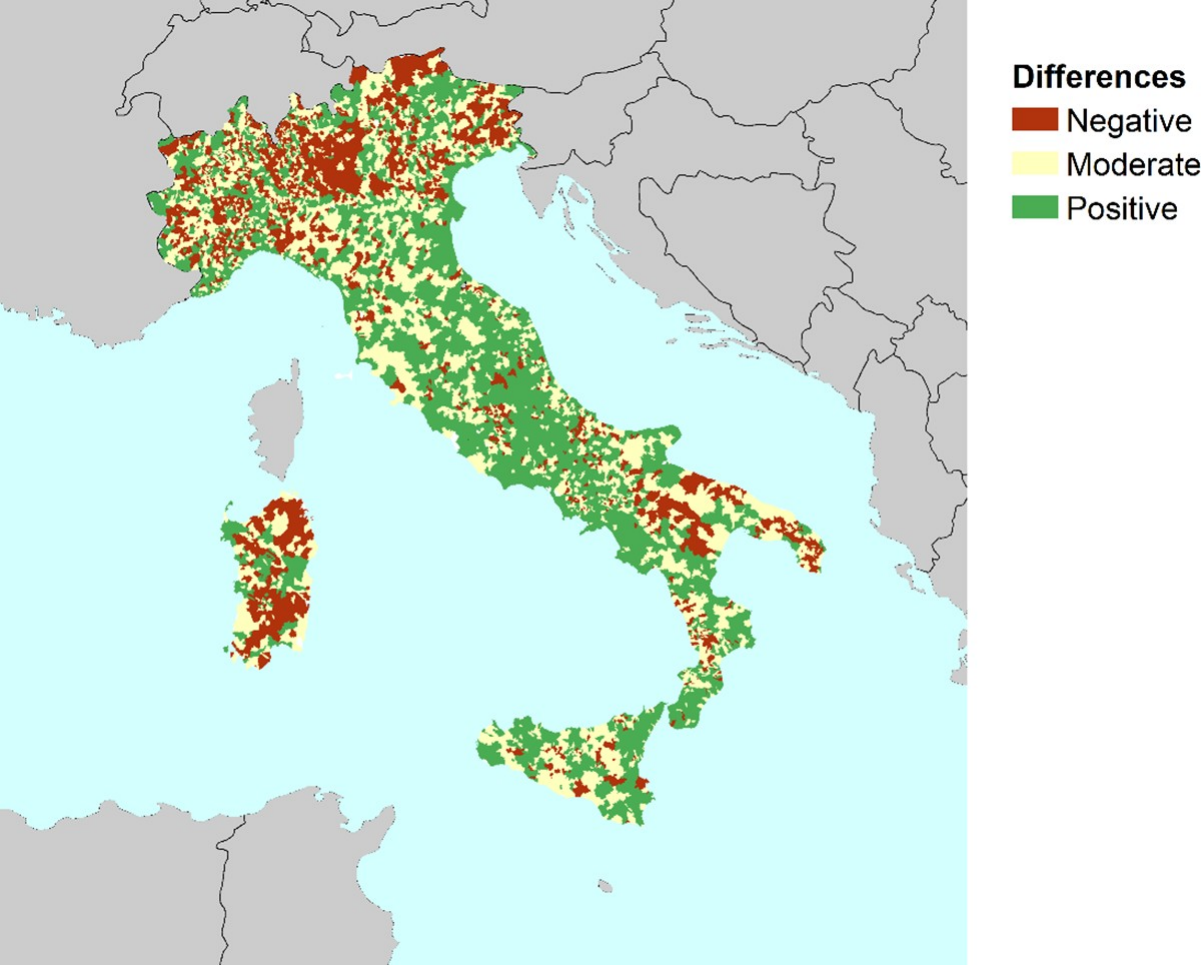
Degree of differences between SVI from ISTAT and CDRI derived from AMP analysis.

The results of the CDRI at the municipal scale are shown in Fig 1 together with the official social vulnerability index (SVI) published by ISTAT. SVI results show higher values in the north, moderate values in the centre and low values in the south of Italy. In general, the CDRI results indicate that the northern and central areas of Italy have higher resilience scores if compared to the SVI results. Fig 2 illustrates the differences among the scores between ISTAT and CDRI, derived from an AMP analysis. The differences are categorized into three groups: i) negative differences correspond to municipalities that are worse-off, shifting from social vulnerability (i.e. SVI) to resilience (i.e. CDRI); ii), moderate differences showing no significant changes, and; iii) positive differences show the areas that are better-off in terms of resilience. Some negative difference clusters can be identified in Fig 2, mostly located in the Italian regions of Lombardy, Trentino, Sardinia, Basilicata and Apulia.

**Fig 3:**
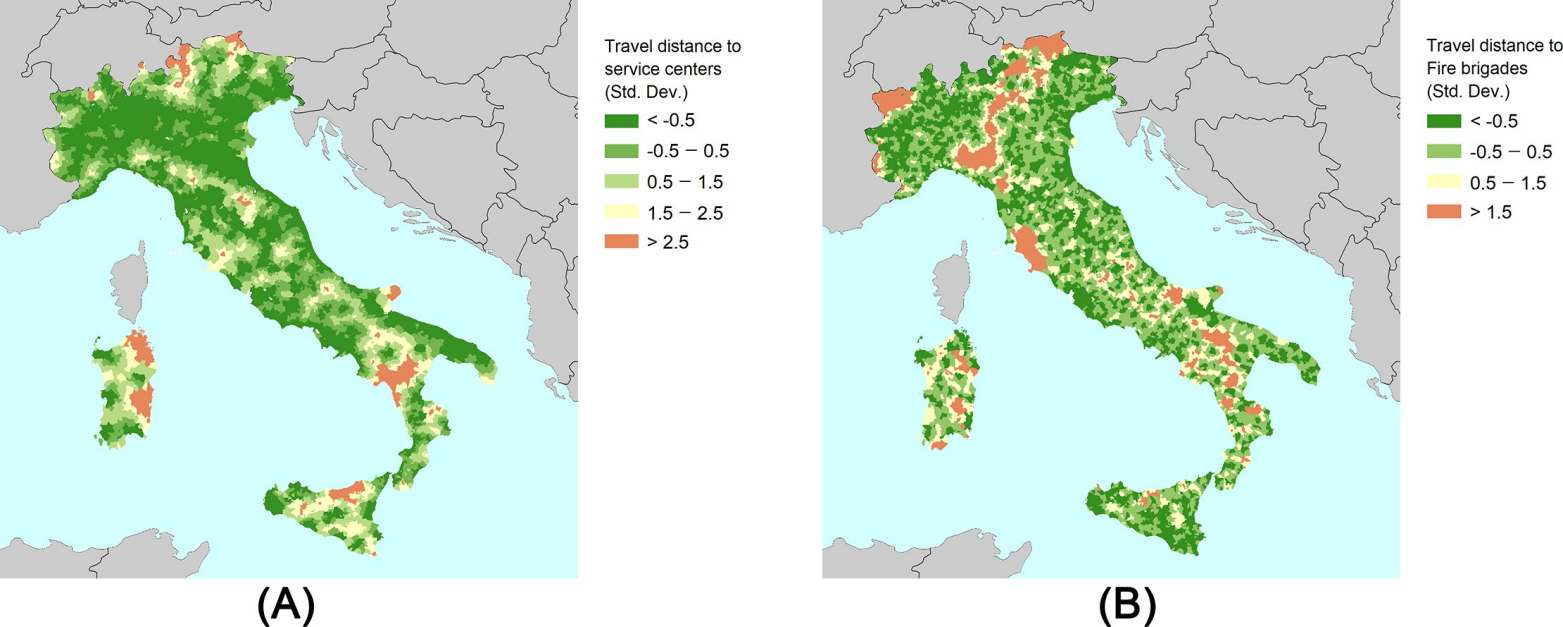
Mapping the original data regarding distance-decay-based attributes. (A) Travel distance to service centers. (B) Travel distance to fire brigades.

While some of the differences between SVI and CDRI indicators are embodied in adaptive capacity dimension, we use the Marzi et al. (2018) adaptive capacity index to interpret the results. Accordingly, despite sizeable intra-regional variabilities, the northern and central regions have higher potentials in terms of economy, infrastructures, technology, level of education and institutional quality regarding the original data (before aggregation). Hence, by adding adaptive capacity elements to the social vulnerability dimension, we can observe higher scores in central and northern Italian territories with respect to the SVI. Since the AMP is a non-compensatory approach, the level of under-performance indicators is a determining factor for the outcome of the aggregation process. To clarify the differences, we examine the indicators which may embody lower performance in the areas with higher score variabilities. To do so, we map the distance-decay-based attributes (travel distance to service centers and fire brigades) to investigate the variabilities. Fig 3 shows the mapping of the original data regarding distance-decay based attributes. Accordingly, it can be observed that the variabilities between two maps are compatible with sizeable differences in the northern territories and the Sardinia region, as illustrated in Fig 3. It can be inferred that the differences between SVI and CDRI may be more sensitive to variations in distance-decay-based attributes, as illustrated by the “travel distance to fire brigades” indicator and shown on the right side of Fig 3.

In order to investigate the correlations between CDRI and each variable, the Pearson correlation coefficient has been calculated [88,109]. The strength of correlations is in the range of “very weak” to “moderate”—classes defined by [127]–and mostly statistically significant (*p* < 0.001) (Table A in S4 Appendix). Therefore, the results are not significantly biased toward any variable, as the correlations suggest.

### Sensitivity and robustness analysis

**Fig 4:**
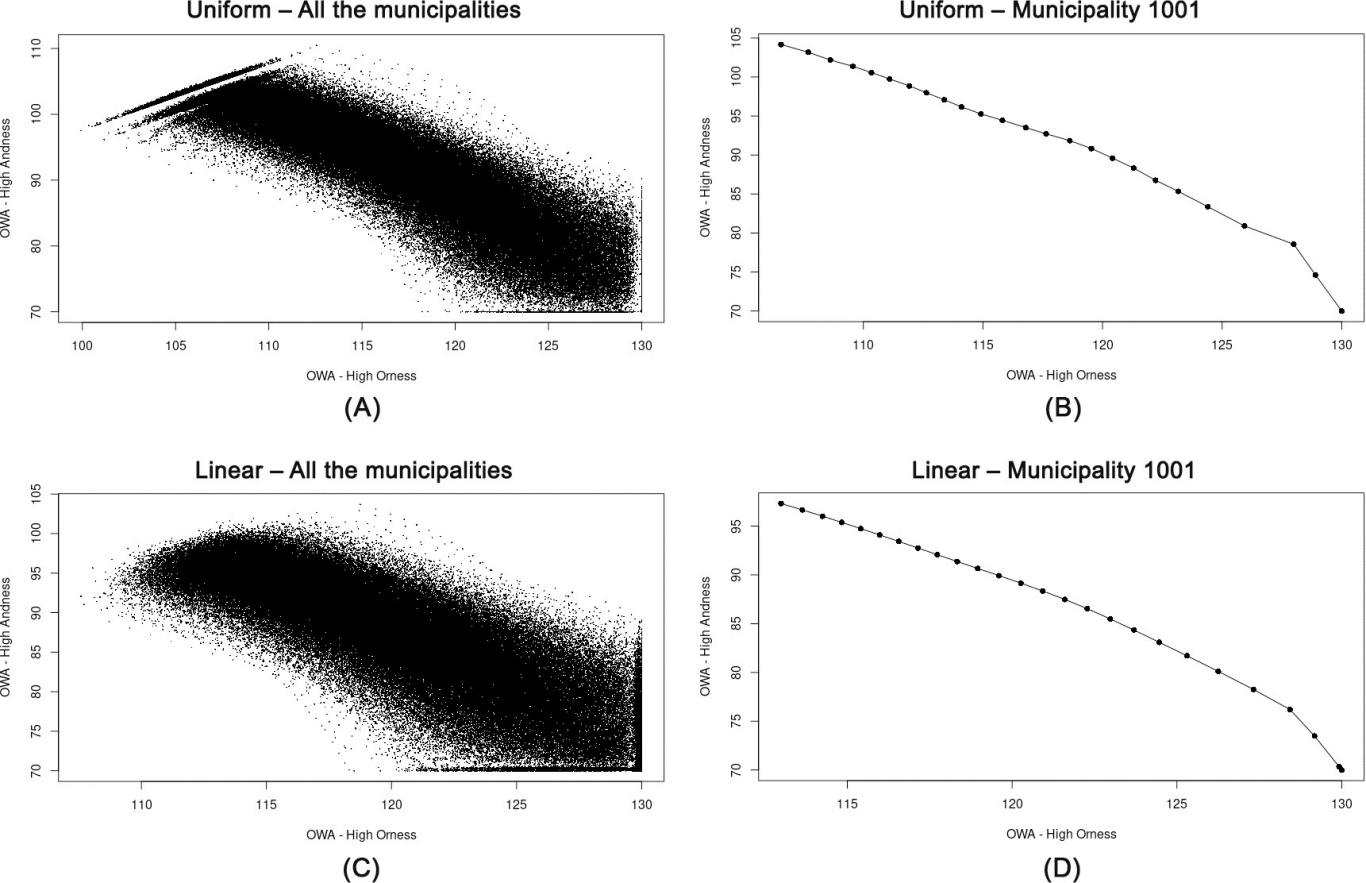
ORNESS vs ANDNESS degrees for all the municipalities by using OWA-AMP. (A) Uniform–all the municipalities. (B) Uniform–municipality 1001. (C) Linear–all the municipalities. (D) Linear–municipality 1001.

In order to test the distribution of OWA weights and the corresponding ORNESS and ANDNESS values, we plotted the scores derived from the OWA by using the transformed data normalized by means of the AMP method for all the municipalities (Fig 4). The results show approximately a linear trend from high ORNESS to high ANDNESS values for both a linear and a uniform distribution of the weights. There is a complementary trade-off between ORNESS and ANDNESS values (*ANDNESS* + *ORNESS* = 1). The first combination has the largest weight assigned to minimum value, corresponding to the largest ANDNESS (and lowest ORNESS). By shifting the proximity from a minimum to a maximum value, the ANDNESS degree diminishes while the ORNESS increases. The graphs validate the assigned spectrum of OWA weights which are employed to perform the sensitivity analysis.

**Fig 5:**
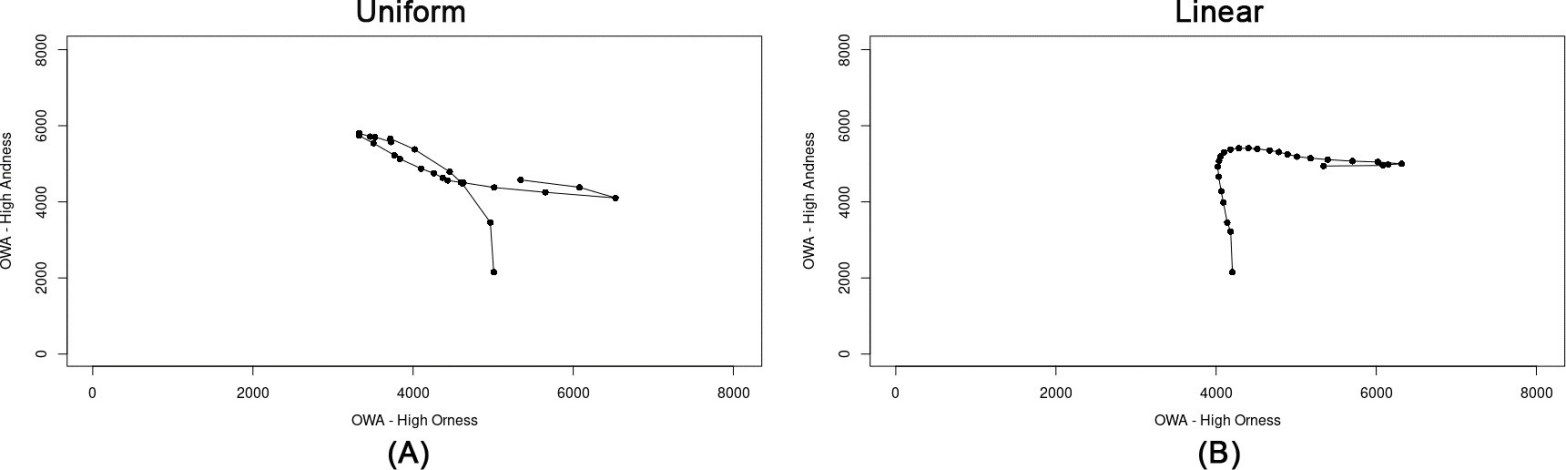
Rank reversals corresponding to ORNESS variations for various OWA weights derived from the OWA-AMP method for municipality 1001. (A) Uniform distribution. (B) Linear distribution.

Next, we applied the same procedure to examine to what extent rankings derived from the same scores plotted in Fig 4 follow the same trend. Fig 5 displays the ORNESS vs ANDNESS degrees for the rankings related to municipality 1001 (Agliè) derived from the OWA-AMP scores. The results show that the rankings follow a non-linear spiral trend which makes it difficult to interpret the trade-offs between the rankings and the degree of ORNESS, as different weight configurations are used for computing the OWA-AMP scored. These results suggest a strong variability of OWA-AMP scores with respect to weights, and thereby low robustness of the rankings.

**Fig 6:**
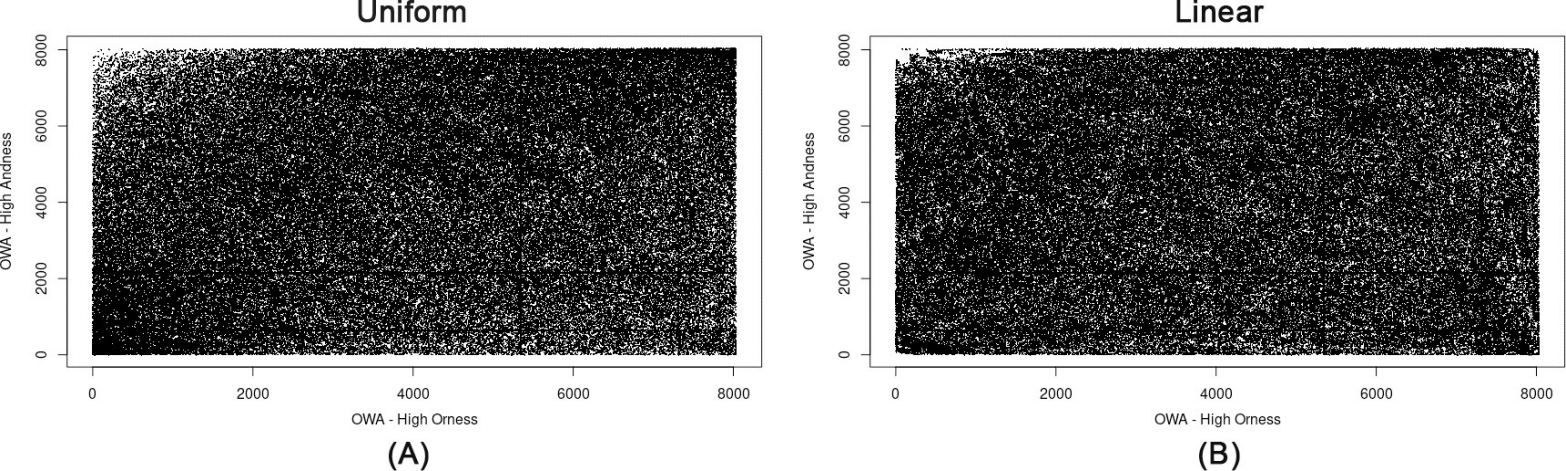
Rank reversals corresponding to ORNESS variations for various OWA weights derived from the OWA-AMP method for all municipalities. (A) Uniform distribution. (B) Linear distribution.

As already explained, by varying the proximity from minimum to maximum values, the ANDNESS values decrease, and the aggregation imposes a higher degree of compensation (additivity) between the indicators. Using additive aggregators with a high degree of compensation implies that underperformance with respect to one or more indicators may not be penalised. However, the level of imbalances plays an important role in the amount of imposed penalization. The decreasing trend observed in Fig 4 is similar for all the OWA combinations, but even a slight variation in the slope for different municipalities may result in variant rank reversals, depending on the endogenous level of imbalances among the indicators for each municipality. This complexity arises from the iterative score variations exposed to different OWA weights for different municipalities, and results in a completely chaotic trend, as shown in Fig 6. The results shown in Fig 6 confirm the strong variability of OWA-AMP scores with respect to OWA weights, and the low robustness of the rankings.

**Fig 7:**
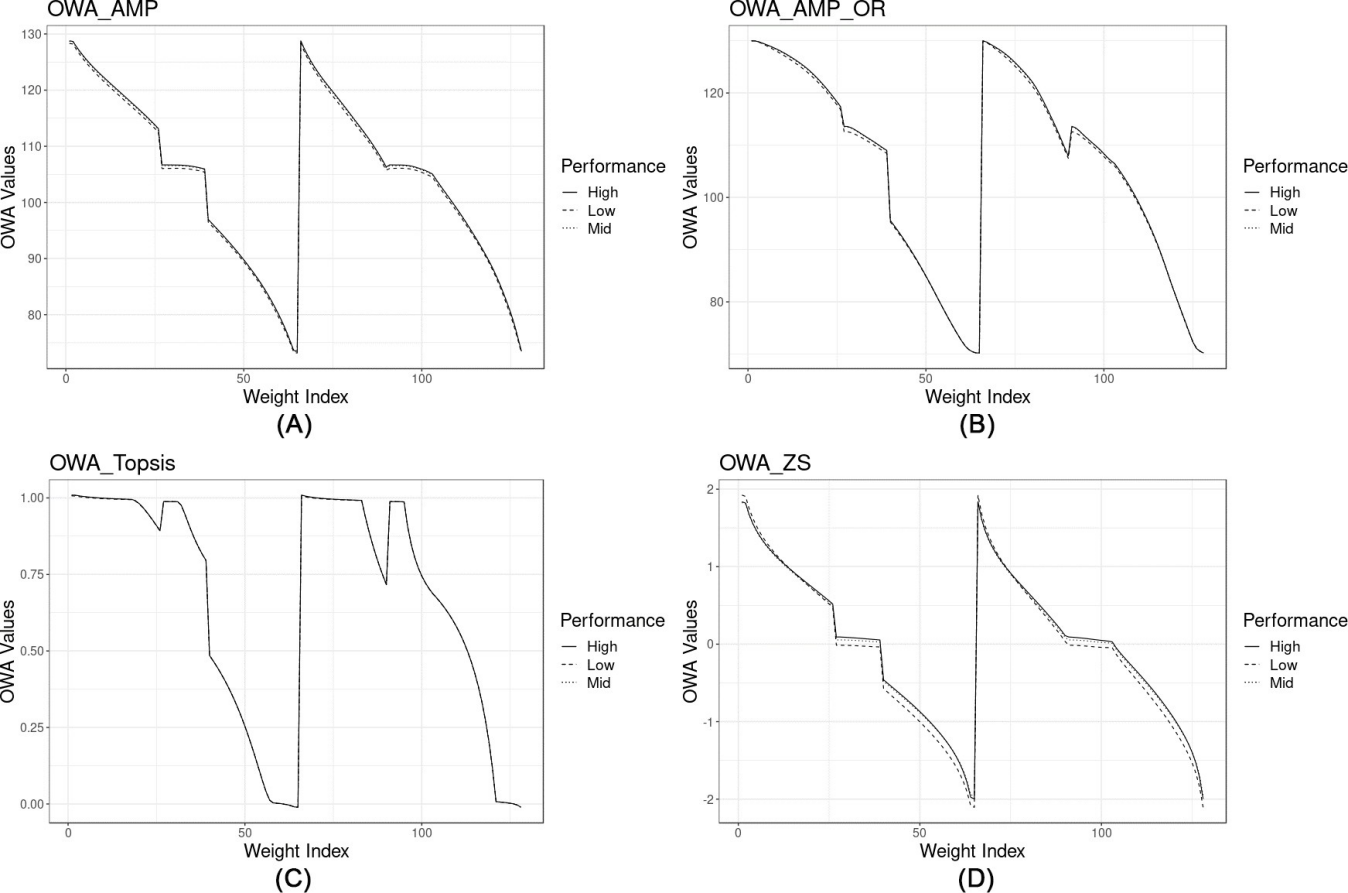
OWA scores derived from various types of normalized data for different combination of weights (ORNESS variations). (A) Box-Cox transformed data normalized using AMP. (B) Original data normalized using AMP. (C) Box-Cox transformed data normalized using Topsis. (D) Box-Cox transformed data normalized using Z-score.

In the next step, we plot the results derived from the OWA aggregation by using all the possible combinations of OWA weights (Fig 7) for various normalization methods, to analyze the sensitivity of the aggregation procedure to different normalization methods.

**Fig 8:**
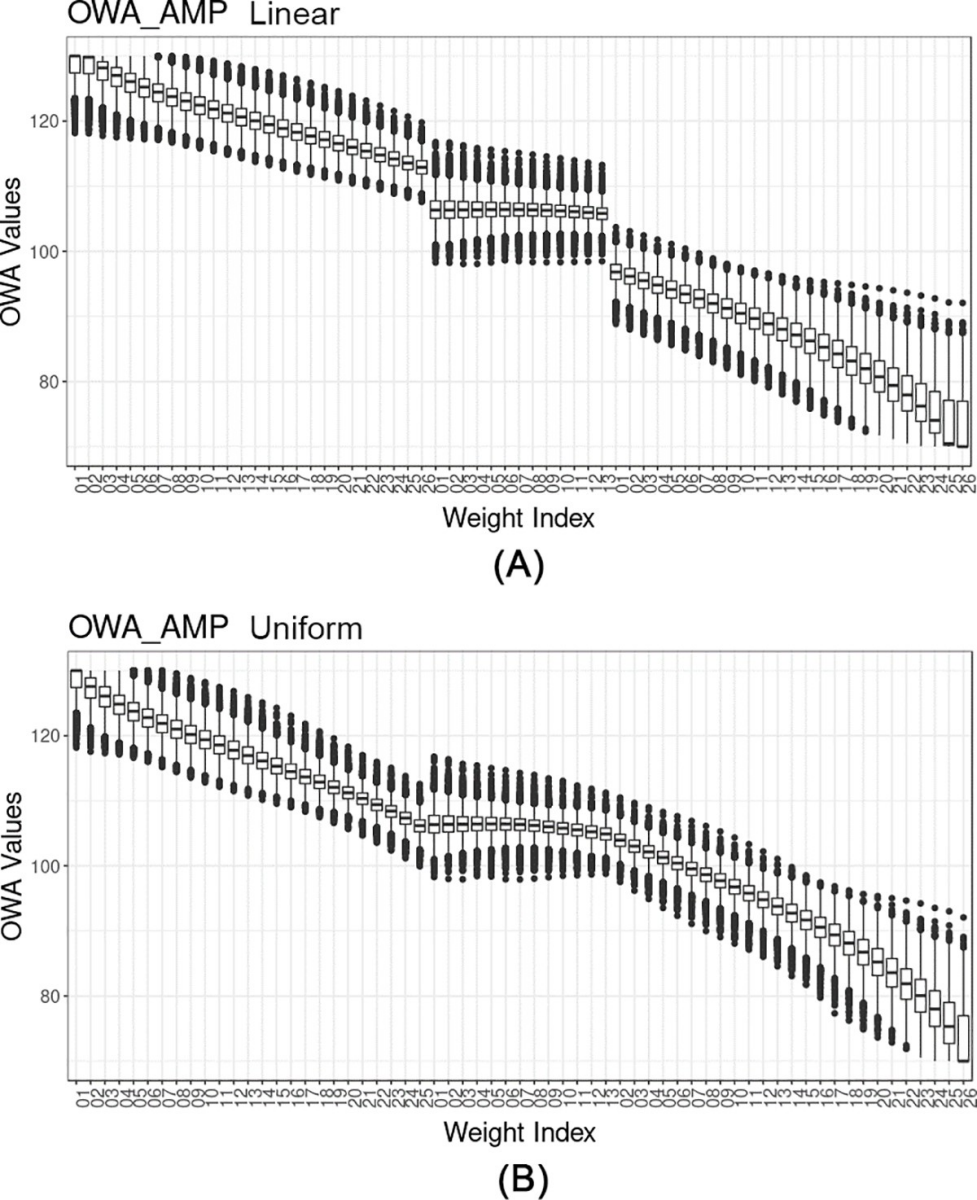
Section of OWA scores derived from AMP normalized data for a different combination of weights for all the municipalities. (A) Linear. (B) Uniform.

The high, medium and low performances represent the alternatives having scores corresponding to median values of 95^th^, 50^th^ and 5^th^ percentiles of CDRI respectively, calculated by using the AMP aggregation. In this way, we can simultaneously involve the alternative performance in the analysis. The results shown in Fig 7 indicate that the level of performance does not significantly affect the OWA values, while, the application of different normalization techniques may yield substantial alterations in the OWA values (e.g. Box-Cox transformed data normalized using Topsis and Z-score). Fig 8 displays the boxplot for only a segment of the OWA-AMP data (Fig 7A), considering the variations among all the municipalities for both linear (top plot) and uniform (bottom plot) distributions (a full set of the cross-sections from various normalizations are provided in the S4 Appendix).

According to Fig 7, applying various normalization methods and transformation yields different results. Cross-comparisons between AMP-BoxCox and AMP-original (Fig 7A and 7B) show how the transformation flattens the anomalies (jumps and sudden declines) that exist in the window type OWA section by equalizing the outliers. The OWA results derived from the AMP and z-score normalized (linear methods) data (Fig 7D) almost follow the same trend: linearly decreasing from high ORNESS to high ANDNESS (except in the range of window type OWA). Nevertheless, the z-score results show a higher variance among OWA scores between low and high-performance alternatives in comparison with both AMP and Topsis. This characteristic can be either advantageous or disadvantageous, since in some cases a lower variance among the results may be preferable. Having results with a higher variance makes it easier to explicitly present the existing differences to policy-makers. The OWA aggregation using non-linear Topsis normalized data (Fig 7C) yields a low-pass filter shape signal having constant results up to a local cut-off, with some fluctuations in the middle and decreasing more or less linearly after passing the cut-off. This property may be interesting for policy-makers in dealing with extreme cases with a high range of compensability. Topsis provides policy-makers with more precise and meaningful information on discontinuities and local minima. Nevertheless, the variance among the low, medium and high performances are very low (Figure A in S4 Appendix), makes it difficult to visually detect the variabilities.

**Fig 9:**
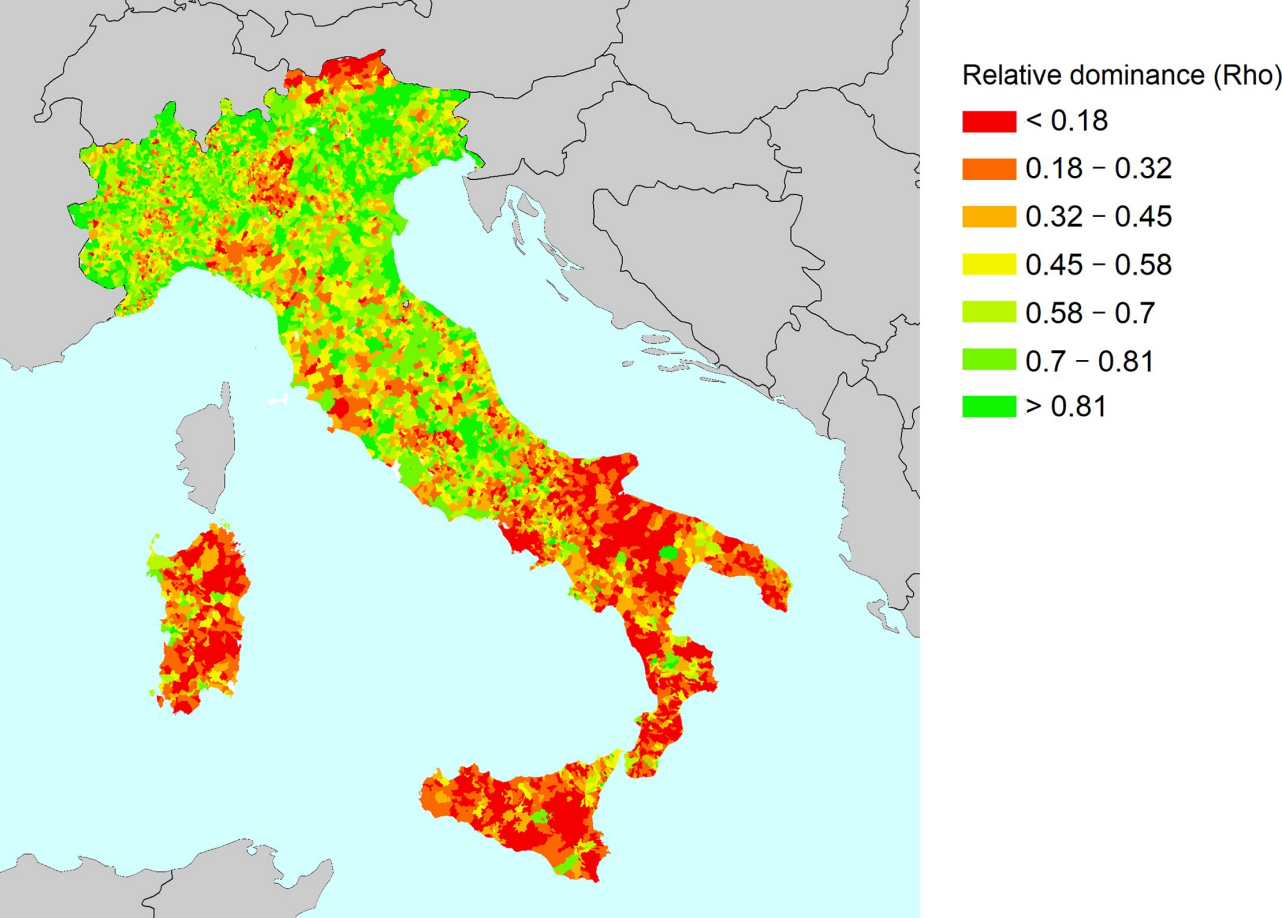
Relative dominance scores derived from 512 OWA configurations.

To sum up, the sensitivity and robustness analyses show that the coupling of the variations in normalization and aggregation methods, and different weight configurations, results in outcomes that may be significantly different, a result that pinpoints the importance of policy-makers of paying close attention to the methodology used for the developing of composite indices. Moreover, depending on the type of policy application and the interest of decision- makers, a certain set of solutions are available, which are introduced in this study. However, even if the results presented and discussed in this paper are so far interesting and promising, further investigation is needed in order to provide robust rankings of the municipalities estimated by means of OWA, if we considering the relative dominance of the municipalities across the simulation. The results of a dominance analysis could be more informative and bring additional insight to identifying relative resilience measures across the municipalities. Fig 9 illustrates the standardized relative dominance scores for Italian municipalities. While this figure considers the total variability of resilience scores, it summarizes all methodological choices addressed in our analysis into a single map. The relative dominance results show a clear north-to-south pattern; in the northern Italian territory, the relative dominance is high, indicating higher resilience against disasters, while the southern Italian territory shows the opposite results. However, some areas within the macro Italian regions present contrasting behavior; for instance, some municipalities in the provinces of Alto Adige and Brescia show low relative dominance, even if they are located in the northern part of Italy, while municipalities in the provinces of Matera and Salerno show high relative dominance values, even if located in the southern part of Italy. The analysis can be further extended by using extra models designed by different normalization, weighting and aggregation schemes.

An analogous robustness analysis can be performed by using other aggregators, such as Logic Scoring of Preference (LSP). In order to provide additional insights, we performed the same procedure by using the LSP method with variant compensation degrees discussed in detail in the S5 Appendix.

## Conclusion

Enhancing disaster resilience is a critical goal of disaster risk reduction and climate change adaptation policies that requires an in-depth understanding of the complex interactions between societies, ecosystems and hazards. Resilience is built up through multiple features, including effective preparedness, risk mitigation, recovery, transformations strengthening social and economic cohesion and trust. Quantitative indicator-based assessments are typically applied to measure resilience by combining multiple performance attributes into composite indices. We describe an original methodological framework used to develop a comprehensive composite municipal disaster resilience index for Italy, and evaluate how multiple methodological choices influence the ensuing results. Our analysis is one of the first attempts to measure resilience at municipal scale, by combining a range of social, economic and environmental features. Our analysis underscores the importance of analysing how robust the scores of a composite index are with respect to the choices of underlying indicators and the degree of compensation allowed by the aggregation methods used. The frameworks such as ours are increasingly important to monitor, report and evaluate (MRE) progress made towards achieving the objectives of the multilateral international agreements such as the Sendai Framework on DRR and the Paris Agreement on climate change [128].

Our analysis builds upon vast research on social vulnerability, and extends the index developed by the Italian National Statistical Office (ISTAT) to include additional original features related to coping and adaptive capacity. We apply advanced normalization and aggregation procedures accompanied by thorough sensitivity and robustness analysis. The choice of indicators used, and their transformations exploit the insights from a mainstream literature review on resilience and multivariate statistical analysis. We first estimate the resilience by using an analogous method to that applied by ISTAT, to be able to compare both indices. Next, we performed a sensitivity analysis by coupling various normalization schemes combined by means of OWA operators with a variant set of weights corresponding to different degrees of compensability. Finally, to measure how robust the ensuing resilience scores are, we have estimated the relative dominance of the municipal rankings over all alternative computation scheme of the composite index.

The latter part entails the most original contribution of our analysis. The ranking of how resilient the Italian municipalities are determined by dominance measures across the multiple aggregation models and configuration of weights. In doing so we reduce the uncertainty introduced through a spectrum of methodological choices. Our framework embraces some characteristics from Tier I and Tier III approaches. We demonstrate a range of statistical methods that allow decision makers to explore the territorial, social and economic disparities, and choose aggregation methods best suitable for the various policy purposes. These methods are based on linear and non-liner normalization approaches combining the OWA and LSP aggregators. Robust resilience rankings are determined by relative dominance across multiple methods. Ideally, the dominance measures can be used as a decision-making benchmark for climate change adaptation and disaster risk management strategies and plans. The proposed methodology reduces the costs and time needed to perform Monte Carlo simulations. As concluded by Bakkensen et al. (2017), it is difficult to validate measures of resilience for infrequent events where specific community and disaster conditions are never exactly the same [16]. However, it is possible to explore how sensitive are the scores of composite indices to methodological choices and assumptions, and clearly communicate the implications of these choices. Our framework.

Our results show considerable variability in the scores derived from an Adjusted Mazziotta-Pareto (AMP) analysis. The municipalities that are left worse-off when turning from social vulnerability to resilience measurements can be observed in the northern regions and Sardinia. Because these results are obtained from a non-compensatory AMP operator, the differences are mostly driven by indicators with performance close to minimum. Depending on the type of policy application and the interest of decision-makers, a certain set of solutions are introduced in this study. For instance, the OWA scores derived from z-score normalized data can better illustrate the disparities among the alternatives with different performance levels. On the other hand, The OWA scores derived from TOPSIS normalized data can provide more precise and meaningful information on discontinuities and local minima which can be more informative while dealing with extreme cases.

Any research on composite indices should indicate whether the metrics applied can be replicated in other places, or are relevant only for a given region, scale or types of hazards. Our framework is replicable, adaptable and extensible. For instance, the service centers and the distance-decay indicators we have used in our analysis may be designed differently in other countries or regions. The framework can also be further extended to include resilience matrix embracing other physical, social or knowledge- (or innovation-) subcomponents and disaster risk management stages [40], and is amendable to community participatory approaches. However, our framework suffers from limitations that are common across the research on indicator-based assessments, such as the limited consistency across geographic scales/administrative levels. Some of these limitations can be overcome methodologically, as we did by focusing on the sensitivity and robustness analyses. For instance, information on the robustness of the rankings can be estimated by means of OWA, if we consider limited (five) performance levels only (e.g. very good, good, average, bad and very bad). From among a variety of possible aggregation methods, we have used an OWA operator. Further research may focus on other aggregators, such as fuzzy t-norms and t-conorms, yielding additional insights. Indicator-based assessment using panel (time-series) data may reveal how resilience changes over time in response to major investments in disaster risk reduction.

## Supporting information

S1 Appendix Background on resilience. (DOCX)

S2 Appendix Conceptual framework and indicators used. (DOCX)

S3 Appendix Data and methodology. (DOCX)

S4 Appendix Results. (DOCX)

S5 Appendix Logic Scoring of Preference (LSP). (DOCX)

S6 Appendix Comparison between original and population corrected form of distance decay indicators and final indices. (DOCX)
