# Supplementary material for: Constructing a comprehensive disaster resilience index: The case of Italy
Source: PLoS One. 2019 Sep 16;14(9):e0221585. doi: 10.1371/journal.pone.0221585 (PMC6746365; doi:10.1371/journal.pone.0221585)
Supplement: S1 Appendix — (DOCX) [file pone.0221585.s001.docx]

**S1 Appendix: Background on resilience**

The term *resilience* has been debated as a cross-disciplinary concept and defined in different ways among different scientific disciplines (Bogardi and Fekete, 2018; Brand and Jax, 2007). Early concept of resilience comes from the ecology and engineering science, being generally understood as the behavior of a dynamic system while exposed to external disturbances (Brand and Jax, 2007; Fletcher and Sarkar, 2013). Engineering resilience refers to the time required for a system to *bounce* *back* to an equilibrium steady state in dynamics close to equilibrium and is focused on stability of the state within the systems’ attraction domain (local stability) (Gallopín, 2006; Grimm and Wissel, 1997; Holling, 1996; Pimm, 1984; Tu, 1994). On the other hand, the ecological (ecosystem) resilience has been explained in a system detached from any equilibrium state and defined as the amount of disturbance a system is able to absorb prior to being transformed to another steady state dynamics outside of the system’s attraction domain. In this case, the stability of the system within the attraction basin in the face of perturbation is not of interest (Folke, 2006; Folke et al., 2004; Gallopín, 2006; Gunderson and Holling, 2002; Gunderson and Pritchard, 2002; Walker et al., 2004).

In recent years, the ecological construct of resilience has been incorporated in the context of social sciences at broader scale with the focus on disaster risk reduction and climate change adaptation disciplines in order to capture the behavioral dynamics of the socio-ecological systems being exposed to natural hazards (Bakkensen et al., 2017; Bogardi and Fekete, 2018; Cutter et al., 2010; Folke, 2006). Resilience in the context of disaster risk reduction has been described as “the ability of a system, community or society exposed to hazards to resist, absorb, accommodate to and recover from the effects of a hazard in a timely and efficient manner” (UNISDR, 2016). In the field of climate change, the latest IPCC report defines resilience as “the capacity of social, economic and environmental systems to cope with a hazardous event or trend or disturbance, responding or reorganizing in ways that maintain their essential function, identity and structure, while also maintaining the capacity for adaptation learning and transformation” (IPCC, 2014a). The IPCC definition addresses the importance of the capacity building for post-disaster period and climate change adaptation. The necessecity of coupling capacity building and bounce back notions has been previously introduced in the context of disaster resilience as “bounce forward” ability explained as a tranformational processes within the context of newly formed dynamics after disater shock (Manyena et al., 2011; Paton and Johnston, 2006). In general terms, resilience is a comprehensive characteristic of complex systems capable of explaining a systems’ ability to deal with disturbances and the effect of stressors (Birkmann et al., 2013).

Resilience is strongly tangled with concept of vulnerability containing similar consideration for assessment design (Fekete, 2018; Parsons et al., 2016; Turner, 2010). There are Some studies considering resilience and vulnerability as counterparts belonging to the same continuum on the opposite poles (Bogardi and Fekete, 2018; Cutter et al., 2008; Manyena et al., 2011; Manyena, 2006). On the contrary, some other studies argue that resilience and vulnerability are strictly related but cannot be used interchangeably as the opposite terms. Gallopín (2006) addresses resilience as the response capacity component of vulnerability and explains fundamental differences using the behavior of dynamic systems being exposed to external perturbations. Accordingly, resilience refers to a state remaining within the considered domain of attraction which result in structural changes in overall system stability. On the other hand, vulnerability expresses the transformations which can traverse a single domain leading to temporary shift in functional state of the system and can be counted as a pre-event characteristic of a system (Adger, 2006; Bakkensen et al., 2017; Cutter et al., 2008; Gallopín, 2006). According to Fekete (2018), resilience in the context of disaster risk reduction, includes certain array of capabilities to compensate vulnerability and mainly regards to the processes experienced when a disaster strikes. Accordingly, vulnerability portrays a broader vision of the degree of susceptibility and coping capacity than resilience. In this sense, a system might be vulnerable to a range of natural hazards and climate related impacts, but it’s not necessary “to possess all types of resiliencies to tackle all those vulnerabilities”. In addition, vulnerability is considered as an internal risk factor mostly assessed through static approaches while the dynamic approaches are usually used in the resilience context (Dixon and Stringer, 2015; Miola et al., 2015). With regards to static approach, vulnerability is the measure of the potential loss at one point in time (pre-disaster) whereas the dynamic approach addresses the integral resilience across all time steps (before, during and after crisis) (Cutter et al., 2003; Linkov et al., 2014). Climate change involves in long term causative processes changing constantly in nonlinear dynamic ways with high degree of uncertainty (Rodin, 2014; Tyler and Moench, 2012; Weichselgartner and Kelman, 2015a). Therefore, resilience discourse can be more appropriate to be employed in the context of climate change than vulnerability (Meerow et al., 2016; Weichselgartner and Kelman, 2015b). It should be mentioned that IPCC (2014a) exhibits the concept of vulnerability as the social and societal construction of the climate risk and describes it as “the propensity or predisposition to be adversely affected”. Hence, vulnerability can be associated with socio-economic and demographic determinants (societal aspects of disaster) previously introduced as “social vulnerability” which influence societies’ preparedness, response and recovery (Birkmann et al., 2013; Cutter et al., 2013, 2003; Fekete, 2018; Terti et al., 2015).

**References**

Adger, W.N., 2006. Vulnerability. Glob. Environ. Chang. 16, 268–281. https://doi.org/10.1016/J.GLOENVCHA.2006.02.006

Bakkensen, L.A., Fox-Lent, C., Read, L.K., Linkov, I., 2017. Validating Resilience and Vulnerability Indices in the Context of Natural Disasters. Risk Anal. 37, 982–1004. https://doi.org/10.1111/risa.12677

Birkmann, J., Cardona, O.D., Carreño, M.L., Barbat, A.H., Pelling, M., Schneiderbauer, S., Kienberger, S., Keiler, M., Alexander, D., Zeil, P., Welle, T., 2013. Framing vulnerability, risk and societal responses: the MOVE framework. Nat. Hazards 67, 193–211. https://doi.org/10.1007/s11069-013-0558-5

Bogardi, J.J., Fekete, A., 2018. Disaster-Related Resilience as Ability and Process: A Concept Guiding the Analysis of Response Behavior before, during and after Extreme Events. Am. J. Clim. Chang. 07, 54–78. https://doi.org/10.4236/ajcc.2018.71006

Brand, F.S., Jax, K., 2007. Focusing the Meaning(s) of Resilience: Resilience as a Descriptive Concept and a Boundary Object. Ecol. Soc. 12. https://doi.org/10.2307/26267855

Cutter, S.L., Barnes, L., Berry, M., Burton, C., Evans, E., Tate, E., Webb, J., 2008. A place-based model for understanding community resilience to natural disasters. Glob. Environ. Chang. 18, 598–606. https://doi.org/10.1016/J.GLOENVCHA.2008.07.013

Cutter, S.L., Boruff, B.J., Shirley, W.L., 2003. Social Vulnerability to Environmental Hazards. Soc. Sci. Q. 84, 242–261. https://doi.org/10.1111/1540-6237.8402002

Cutter, S.L., Burton, C.G., Emrich, C.T., 2010. Disaster Resilience Indicators for Benchmarking Baseline Conditions. J. Homel. Secur. Emerg. Manag. 7. https://doi.org/10.2202/1547-7355.1732

Cutter, S.L., Emrich, C.T., Morath, D.P., Dunning, C.M., 2013. Integrating social vulnerability into federal flood risk management planning. J. Flood Risk Manag. 6, 332–344. https://doi.org/10.1111/jfr3.12018

Dixon, J., Stringer, L., 2015. Towards a Theoretical Grounding of Climate Resilience Assessments for Smallholder Farming Systems in Sub-Saharan Africa. Resources 4, 128–154. https://doi.org/10.3390/resources4010128

Fekete, A., 2018. Societal resilience indicator assessment using demographic and infrastructure data at the case of Germany in context to multiple disaster risks. Int. J. Disaster Risk Reduct. 31, 203–211. https://doi.org/10.1016/J.IJDRR.2018.05.004

Fletcher, D., Sarkar, M., 2013. Psychological resilience: A review and critique of definitions, concepts, and theory. Eur. Psychol. 18, 12.

Folke, C., 2006. Resilience: The emergence of a perspective for social–ecological systems analyses. Glob. Environ. Chang. 16, 253–267. https://doi.org/10.1016/J.GLOENVCHA.2006.04.002

Folke, C., Carpenter, S., Walker, B., Scheffer, M., Elmqvist, T., Gunderson, L., Holling, C.S., 2004. Regime Shifts, Resilience, and Biodiversity in Ecosystem Management. Annu. Rev. Ecol. Evol. Syst. 35, 557–581. https://doi.org/10.1146/annurev.ecolsys.35.021103.105711

Gallopín, G.C., 2006. Linkages between vulnerability, resilience, and adaptive capacity. Glob. Environ. Chang. 16, 293–303. https://doi.org/10.1016/J.GLOENVCHA.2006.02.004

Grimm, V., Wissel, C., 1997. Babel, or the ecological stability discussions: an inventory and analysis of terminology and a guide for avoiding confusion. Oecologia 109, 323–334. https://doi.org/10.1007/s004420050090

Gunderson, L.H., Holling, C.S. (Eds.), 2002. Panarchy: understanding transformations in human and natural systems. Island Press, Washington, D.C.

Gunderson, L.H., Pritchard, L. (Eds.), 2002. Resilience and the behaviour of large-scale systems. Island Press, Washington, D.C.

Holling, C.S., 1996. Engineering resilience versus ecological resilience. Eng. within Ecol. constraints 31, 32.

IPCC, 2014a. Annex II: Glossary, in: Mach, K.J., Planton, S., von Stechow, C. (Eds.), Cli- Mate Change 2014: Synthesis Report. Contribution of Working Groups I, II and III to the Fifth Assessment Report of the Intergovernmental Panel on Climate Change. Geneva, Switzerland, pp. 117–130.

IPCC, 2014b. Climate Change 2014: Impacts, Adaptation, and Vulnerability. Part A: Global and Sectoral Aspects. Contribution of Working Group II to the Fifth Assessment Report of the Intergovernmental Panel on Climate Change, in: Field, C.B., Barros, V.R. Dokken, D.J., Mach, K.J., Mastrandrea, M.D., Bilir, T.E Chatterjee, M., Ebi, K.L., Estrada, Y.O., Genova, R.C., Girma, B., Kissel, E.S., Levi, A.N., MacCracken, S., Mastrandrea, P.R. and White, L.L. (Eds.), . Cambridge University Press, Cambridge, United Kingdom and New York, NY, USA, p. 1132.

Linkov, I., Bridges, T., Creutzig, F., Decker, J., Fox-Lent, C., Kröger, W., Lambert, J.H., Levermann, A., Montreuil, B., Nathwani, J., Nyer, R., Renn, O., Scharte, B., Scheffler, A., Schreurs, M., Thiel-Clemen, T., 2014. Changing the resilience paradigm. Nat. Clim. Chang. 4, 407–409. https://doi.org/10.1038/nclimate2227

Manyena, B., O’Brien, G., O’Keefe, P., Rose, J., 2011. Disaster resilience: a bounce back or bounce forward ability? Local Environ. Int. J. Justice Sustain. 16, 417–424. https://doi.org/10.1080/13549839.2011.583049

Manyena, S.B., 2006. The concept of resilience revisited. Disasters 30, 434–450. https://doi.org/10.1111/j.0361-3666.2006.00331.x

Meerow, S., Newell, J.P., Stults, M., 2016. Defining urban resilience: A review. Landsc. Urban Plan. 147, 38–49. https://doi.org/10.1016/J.LANDURBPLAN.2015.11.011

Miola, A., Paccagnan, V., Papadimitriou, E., Mandrici, A., 2015. Climate resilient development index: theoretical framework, selection criteria and fit for purpose indicators. Eur. Comm. https://doi.org/10.2788/07628

Parsons, M., Glavac, S., Hastings, P., Marshall, G., McGregor, J., McNeill, J., Morley, P., Reeve, I., Stayner, R., 2016. Top-down assessment of disaster resilience: A conceptual framework using coping and adaptive capacities. Int. J. Disaster Risk Reduct. 19, 1–11. https://doi.org/10.1016/j.ijdrr.2016.07.005

Paton, D., Johnston, D.M., 2006. Disaster resilience : an integrated approach. Charles C Thomas, Illinois, U.S.A.

Pimm, S.L., 1984. The complexity and stability of ecosystems. Nature 307, 321–326. https://doi.org/10.1038/307321a0

Rodin, J., 2014. The resilience dividend: being strong in a world where things go wrong, Public Affairs. New York, NY .

Terti, G., Ruin, I., Anquetin, S., Gourley, J.J., 2015. Dynamic vulnerability factors for impact-based flash flood prediction. Nat. Hazards 79, 1481–1497. https://doi.org/10.1007/s11069-015-1910-8

Tu, P.N.V., 1994. Dynamical Systems--an Introduction with Applications in Economics and Biology, second. ed, Springer. Berlin.

Turner, B.L., 2010. Vulnerability and resilience: Coalescing or paralleling approaches for sustainability science? Glob. Environ. Chang. 20, 570–576. https://doi.org/10.1016/J.GLOENVCHA.2010.07.003

Tyler, S., Moench, M., 2012. A framework for urban climate resilience. Clim. Dev. 4, 311–326. https://doi.org/10.1080/17565529.2012.745389

UNISDR, 2016. Report of the open-ended intergovernmental expert working group on indicators and terminology relating to disaster risk reduction.

Walker, B., Holling, C.S., Carpenter, S.R., Kinzig, A., 2004. Resilience, adaptability and transformability in social–ecological systems. Ecol. Soc. 9.

Weichselgartner, J., Kelman, I., 2015a. Geographies of resilience. Prog. Hum. Geogr. 39, 249–267. https://doi.org/10.1177/0309132513518834

Weichselgartner, J., Kelman, I., 2015b. Geographies of resilience. Prog. Hum. Geogr. 39, 249–267. https://doi.org/10.1177/0309132513518834
