# Supplementary material for: Constructing a comprehensive disaster resilience index: The case of Italy
Source: PLoS One. 2019 Sep 16;14(9):e0221585. doi: 10.1371/journal.pone.0221585 (PMC6746365; doi:10.1371/journal.pone.0221585)
Supplement: S2 Appendix — (DOCX) [file pone.0221585.s002.docx]

**S2 Appendix: Conceptual framework and indicators used**

***Access to services****:* Distance-decay accessibility (travel time and distance) to emergency services such as hospitals, fire & rescue stations has been extensively considered in previous studies (1–6). Access to health, education services and other assets plays a crucial role in reducing inequalities and climate resilient pathways (7–9). In Italy, accessibility to essential services such as education, health and mobility is a defining feature of disadvantaged (also called inner) areas (10). Distance and travel time to service centres has been calculated by using the commuting matrices of distance and travel time between all Italian municipalities provided by ISTAT (11). Service centres are defined as municipalities that have: a) a full range of secondary schools; b) at least one first level DEA hospital, and; c) at least one “silver-type” railway station. Data on municipalities hosting essential services were obtained from Barca et al., 2014. The distance between municipalities is measured by municipality centroids (2013), while travel time is estimated by using the TomTom MultiNet road network (2013).

***Institutions:*** Accountability of and trust in institutions and officials has been mentioned as an important element of organizational resilience, which can empower the risk perception of a society and boost social cohesion (12–14). According to Larsen (2014), a well-functioning democracy is positively correlated with the level of social trust in the system. Hooghe and Stiers (2016) argue that participation in elections as a representative element of democracy increases social and political trust regardless of who wins or loses in an election. In the case of Italy, despite past diffidence, recent trends show that participation generates trust and, as a consequence, confidence in institutions is increasing significantly among the population showing higher participation rates (16). In our study, we consider participation rates in elections as a proxy to evaluate trust in institutions. The data for elections was obtained from the Ministero dell'Interno. We computed the average participation rates in 2016 constitutional referendum (17) and 2017 administrative election (18) in Italy.

***Housing conditions:*** Housing conditions and dwellings are referred to as infrastructure (12,19–21). The quality and occupancy rate of dwellings can affect the degree of physical damage and vulnerability of the residents in time of disaster shock (2,22–24). Hence, empowering the elements regarding the housing and dwellings can promote coping capacity and consequently resilience. Disaster risk management studies focus mainly on residential buildings and dwellings. “Residential housing stock and age” has been previously used by Cutter et al. (2014) and Frigerio et al. (2016) to assess social vulnerability and resilience. In addition, the percentage of “mean age of household construction units” and “residential housing units built prior to 1970’s” have been mentioned in the studies conducted by Aroca-Jimenez et al. (2017) and Cutter et al (2014). According to Besagni & Borgarello (2018), the number of residential housing units constructed before 1970’s has the highest share of the total number of residential units in Italy. The combination of this indicator with the conservation status of the residential buildings can provide an estimate of the overall quality of the residential housing units in Italy. To do so, we used quality rate of dwellings, rate of empty dwellings and index of overcrowded residences. The quality rate of the dwellings was calculated as a ratio between the dwellings with excellent and good conservation status, and the dwellings with mediocre and poor conservation status. The index of the overcrowded residences is defined as a percentage ratio between the population living in dwellings with a surface of less than 40 square meters and more than 4 occupants or in 40-59 square meters and more than 5 occupants or in 60-79 square meters and more than 6 occupants, and the total resident population (25,26).

***Cohesion:***  cohesion refers to a “bond that keeps societies integrated” (14). refers to a “bond that keeps societies integrated” and comprises elements of family structure, dependencies and commuting rates. *Family structure* has been widely used in literature as one of the core components of resilience and social vulnerability assessments. According to Cutter et al. (2003), Parsons et al. (2016) and Frigerio & De Amicis (2016), families with large numbers of dependents or single-parent households often have limited ﬁnances to outsource care for dependents, and thus must balance work responsibilities and care for family members. Family size may also affect the evacuation ability and resilience to natural hazards. In our study, we considered indices of single parent, large and small families (COH_1, COH_2 and COH_3 respectively).

*Dependency* dimension of the cohesion focus on the population who are economically and socially marginalized and require additional support in the post-disaster period (27). The aforesaid population include extremes of the age spectrum, woman and families need assistance (20,25,26,28). We chose index for elderly and minor dependence an old age index to address the extremes of age spectrum. Index for elderly dependence is a percentage ratio between the population aged 65 and over, and the population aged 15 to 64 (25,26). Index for minor dependence is calculated as a percentage ratio between the population below 15, and the population aged 15 to 64 (25,26). The old age index is a percentage ratio of the population aged 65 and more over 0-14 years age group (25,26). The index of the families with assistance need is defined as a percentage ratio between the number of families with at least two members, without cohabitants, with all components aged 65 and over and with the presence of at least one member of 80 years and over, and the total of households (25,26). According to Cutter et al (2003), women can have a more difﬁcult time during recovery than men, often due to sector-speciﬁc employment, lower wages, and family care responsibilities. To address this issue, we used the women’s participation in the labor market indicator. This indicator is defined as a percentage ratio between the active female resident population and the female resident population of the same age group (25,26).

We considered commuting rate for study or work, containment and attraction indices to assess the *commuting rate* component of the cohesion. Containment index (COH_10) is a measure of the number of people who commute within their residence municipality, and the Attraction Index (COH_11) is defined as the capability of municipalities to appeal to commuters. Such indices have been previously employed by Fekete (2009) and Frigerio (2016) to assess the social vulnerability. In contrary to containment ratio, the commuting and attraction ratios may decrease the social engagement and integrity, and subsequently the cohesion in a society. Cutter et al. (2008) identified “residential mobility with more people moving into areas where they were unfamiliar with the local hazards and ways of coping with them” as one of the drivers of rising potential loss and vulnerability to natural hazards. The total number of individuals traveling amongst municipalities has been utilized to calculate the containment and attraction indices. This number has been calculated using the commuting matrices of distance and travel time between all Italian municipalities provided by Italian National Statistic Office.

*Education:* Level of education is often used as a proxy degree of preparedness for dealing with shocks and reinforces responses (19,21,29–31). Higher education levels have been considered as elements of adaptive capacity that can affect the productivity yields in R&D and innovation sectors (32–36).According to Cutter et al. (2003), education is linked to socioeconomic status, with higher educational attainment resulting in greater lifetime earnings. Lower education constrains the ability to understand warning information and access to recovery information. We consider illiteracy rate, high and low education indices to evaluate the education component of resilience. The low education index is calculated as the percentage of residents with elementary license (5th grade), while the high education index is defined as the percentage of residents having graduate degrees.

*Economic resources:* Economic resources play an important role in boosting resilience and adaptive capacity (8,37,38). Economic resources enable communities to absorb and recover from losses more quickly due to insurance, social safety nets, and entitlement programs (27). Per capita income, income distribution, poverty rates and unemployment have been employed to assess economic resources (35,36,39–41). In our study, we also considered land valuation, which can support emergency response, recovery and reconstruction after disaster shock (42–44). Income, Gini, unemployment and share of families with potential economic hardship. For income distribution we used GINI coefficient which measures the extent to which the distribution of income within a country deviates from a perfectly equal distribution. A coefficient of 0 expresses perfect equality where everyone has the same income, while a coefficient of 100 expresses full inequality where only one person has all the income. Income data was obtained from the Department of Finance (2018) and was used to calculate the GINI coefficient. In particular, we used the GiniWegNeg R package (46), which makes it possible to estimate Gini-based coefficients for cases that also include negative incomes. Share of families with potential economic hardship is defined as a percentage ratio between the number of families with children with the reference person aged up to 64 in which no employee is occupied or withdrawn from work, and the total household (25,26). Land values were estimated as cadastral stock and obtained from the Agenzia Entrate database (2013) at the municipal level and covering the entire Italian territory.

*Environment:* Environmental and ecosystem aspects of resilience have been embedded in the ecological/ecosystem dimension in previous studies (12,20). According to an IPCC report, conservation of protected areas and ecological corridors can be important for ecosystem-based climate adaptation and disaster risk reduction strategies (48). Expansion and conservation of protected areas and ecological corridors leads to preserving ecosystem services and ecological resilience, which are the core elements of green infrastructure planning in Europe (49,50). The share of protected lands from the total area was estimated on the basis of the extension of the Special Protection Areas (SPA) and the Sites of Community Importance (SCIs) under the Natura 2000 Network (51,52). For the ecological corridors, we used the database developed by the European Environment Agency in the framework of the EU Copernicus programme (53). The database contains Green Linear Elements (GLE) and structural landscape elements which act as important dispersion vectors of biodiversity.

**References**

1. Fernandez P, Mourato S, Moreira M. Social vulnerability assessment of flood risk using GIS-based multicriteria decision analysis. A case study of Vila Nova de Gaia (Portugal). Geomatics, Nat Hazards Risk [Internet]. 2016 Jul 3 [cited 2018 Aug 17];7(4):1367–89. Available from: http://www.tandfonline.com/doi/full/10.1080/19475705.2015.1052021

2. Aroca-Jimenez E, Bodoque J, Garcia J. Construction of an Integrated Social Vulnerability Index in urban areas prone to flash flooding. Nat Hazards Earth Syst Sci [Internet]. 2017 [cited 2017 Jul 15];17(9):1541. Available from: http://www.nat-hazards-earth-syst-sci-discuss.net/nhess-2016-408/nhess-2016-408.pdf

3. Fekete A. Validation of a social vulnerability index in context to river-floods in Germany. Nat Hazards Earth Syst Sci [Internet]. 2009 Mar 19 [cited 2018 Jan 13];9(2):393–403. Available from: http://www.nat-hazards-earth-syst-sci.net/9/393/2009/

4. Kienberger S, Contreras D, Zeil P. Spatial and Holistic Assessment of Social, Economic, and Environmental Vulnerability to Floods—Lessons from the Salzach River Basin, Austria. Assess Vulnerability to Nat Hazards [Internet]. Elsevier; 2014 Jan 1 [cited 2018 Aug 17];53–73. Available from: https://www.sciencedirect.com/science/article/pii/B9780124105287000035

5. Carreño M-L, Cardona OD, Barbat AH. Urban Seismic Risk Evaluation: A Holistic Approach. Nat Hazards [Internet]. Springer Netherlands; 2007 Jan 9 [cited 2018 Aug 17];40(1):137–72. Available from: http://link.springer.com/10.1007/s11069-006-0008-8

6. Haddow GD, Bullock JA, Coppola DP. Introduction to Emergency Management. Butterworth-Heinemann. Burlington, MA; 2011.

7. UNDP. Sustainable Development Goals [Internet]. 2017 [cited 2017 Apr 10]. Available from: http://www.undp.org/content/undp/en/home/sustainable-development-goals/goal-16-peace-justice-and-strong-institutions/targets/

8. ESPON. ESPON CLIMATE-Climate Change and Territorial Effects on Regions and Local Economies. Luxembourg.; 2011;

9. Denton F, Wilbanks TJ, Abeysinghe AC, Burton I, Gao Q, Lemos MC, et al. Climate-resilient pathways: adaptation, mitigation, and sustainable developmen. In: Field CB, Barros VR, Dokken DJ, Mach KJ, Mastrandrea, M.D. Bilir TE, Chatterjee M, et al., editors. Climate Change 2014: Impacts, Adaptation, and Vulnerability Part A: Global and Sectoral Aspects Contribution of Working Group II to the Fifth Assessment Report of the Intergovernmental Panel on Climate Change [Internet]. Cambridge, United Kingdom and New York, NY, USA: Cambridge University Press ; 2014 [cited 2018 Aug 17]. p. 1101–13. Available from: https://www.ipcc.ch/pdf/assessment-report/ar5/wg2/WGIIAR5-Chap20_FINAL.pdf

10. Barca F, Casavola P, Lucatelli S. A strategy for Inner Areas in Italy: definition, objectives, tools and governance. Mater Uval Ser [Internet]. 2014;31. Available from: http://www.agenziacoesione.gov.it/opencms/export/sites/dps/it/documentazione/servizi/materiali_uval/Documenti/MUVAL_31_Aree_interne_ENG.pdf

11. ISTAT. Matrici di contiguità, distanza e pendolarismo [Internet]. 2013 [cited 2018 Aug 21]. Available from: https://www.istat.it/it/archivio/157423

12. Cutter SL, Barnes L, Berry M, Burton C, Evans E, Tate E, et al. A place-based model for understanding community resilience to natural disasters. Glob Environ Chang [Internet]. Pergamon; 2008 Oct 1 [cited 2018 Aug 9];18(4):598–606. Available from: https://www.sciencedirect.com/science/article/pii/S0959378008000666

13. Rufat S, Tate E, Burton CG, Maroof AS. Social vulnerability to floods: Review of case studies and implications for measurement. Int J Disaster Risk Reduct [Internet]. Elsevier; 2015 Dec 1 [cited 2018 Aug 20];14:470–86. Available from: https://www.sciencedirect.com/science/article/pii/S2212420915300935

14. Larsen CA. Social cohesion: Definition, measurement and developments. Inst Statskundskab, Aalborg Univ [Internet]. Institut for Statskundskab, Aalborg Universitet; 2014 [cited 2018 Aug 17]; Available from: http://www.forskningsdatabasen.dk/en/catalog/2262055869

15. Hooghe M, Stiers D. Elections as a democratic linkage mechanism: How elections boost political trust in a proportional system. Elect Stud [Internet]. Pergamon; 2016 Dec 1 [cited 2018 Aug 20];44:46–55. Available from: https://www.sciencedirect.com/science/article/pii/S0261379416300877

16. Diamanti I. Rapporto gli italiani e lo stato [Internet]. 2017 [cited 2018 Aug 20]. Available from: http://www.demos.it/rapporto.php

17. Ministero dell’Interno. Dati refendum 4 dicembre 2016 [Internet]. Dipartimento per gli affari interni e territoriali. 2016 [cited 2019 Jun 28]. Available from: https://dait.interno.gov.it/elezioni/open-data/dati-refendum-4-dicembre-2016

18. Ministero dell’Interno. Dati elezioni amministrative 11 giugno 2017 [Internet]. Dipartimento per gli affari interni e territoriali. 2017 [cited 2019 Jun 28]. Available from: https://dait.interno.gov.it/elezioni/open-data/dati-elezioni-amministrative-11-giugno-2017

19. Parsons M, Glavac S, Hastings P, Marshall G, McGregor J, McNeill J, et al. Top-down assessment of disaster resilience: A conceptual framework using coping and adaptive capacities. Int J Disaster Risk Reduct. 2016;19:1–11.

20. Cutter SL, Ash KD, Emrich CT. The geographies of community disaster resilience. Glob Environ Chang [Internet]. Pergamon; 2014 Nov 1 [cited 2018 Aug 12];29:65–77. Available from: https://www.sciencedirect.com/science/article/pii/S0959378014001459

21. Beccari B. A Comparative Analysis of Disaster Risk, Vulnerability and Resilience Composite Indicators. PLoS Curr [Internet]. Public Library of Science; 2016 [cited 2018 Aug 13]; Available from: http://currents.plos.org/disasters/?p=26273

22. Frigerio I, Carnelli F, Cabinio M, De Amicis M. Spatiotemporal Pattern of Social Vulnerability in Italy. Int J Disaster Risk Sci [Internet]. Beijing Normal University Press; 2018 Apr 30 [cited 2018 May 25];1–14. Available from: http://link.springer.com/10.1007/s13753-018-0168-7

23. Ludy J, Kondolf GM. Flood risk perception in lands “protected” by 100-year levees. Nat Hazards [Internet]. Springer Netherlands; 2012 Mar 4 [cited 2018 Aug 19];61(2):829–42. Available from: http://link.springer.com/10.1007/s11069-011-0072-6

24. Flanagan BE, Gregory EW, Hallisey EJ, Heitgerd JL, Lewis B. A Social Vulnerability Index for Disaster Management. J Homel Secur Emerg Manag [Internet]. De Gruyter; 2011 Jan 5 [cited 2018 Aug 19];8(1). Available from: https://www.degruyter.com/view/j/jhsem.2011.8.issue-1/jhsem.2011.8.1.1792/jhsem.2011.8.1.1792.xml

25. ISTAT. L ’indice di vulnerabilità sociale e materiale [Internet]. 2017 [cited 2018 May 25]. Available from: http://ottomilacensus.istat.it/fileadmin/download/Indice_di_vulnerabilità_sociale_e_materiale.pdf

26. ISTAT. 8milaCensus [Internet]. 2015 [cited 2018 Aug 20]. Available from: https://www.istat.it/it/archivio/160823

27. Cutter SL, Boruff BJ, Shirley WL. Social Vulnerability to Environmental Hazards. Soc Sci Q [Internet]. 2003 [cited 2017 Mar 22];84(2):242–61. Available from: http://doi.wiley.com/10.1111/1540-6237.8402002

28. Parsons M, Glavac S, Hastings P, Marshall G, McGregor J, McNeill J, et al. Top-down assessment of disaster resilience: A conceptual framework using coping and adaptive capacities. Int J Disaster Risk Reduct [Internet]. Elsevier; 2016 Oct 1 [cited 2018 May 13];19:1–11. Available from: https://www.sciencedirect.com/science/article/pii/S2212420916300887

29. Frigerio I, De Amicis M. Mapping social vulnerability to natural hazards in Italy: A suitable tool for risk mitigation strategies. Environ Sci Policy [Internet]. Elsevier; 2016 Sep 1 [cited 2017 Dec 3];63:187–96. Available from: http://www.sciencedirect.com/science/article/pii/S1462901116302702

30. Thomas DSK, Phillips BD, Lovekamp WE, Fothergill A. Social Vulnerability to Disasters [Internet]. 2nd ed. Boca Raton: CRC Press; 2013 [cited 2018 Aug 18]. 514 p. Available from: https://www.taylorfrancis.com/books/9781466516380

31. Poljanšek K, Marin Ferrer M, De Groeve T, Clark I. Science for disaster risk management 2017: knowing better and losing less. EUR 28034 EN, Publ Off Eur Union [Internet]. 2017 [cited 2017 Sep 18]; Available from: https://reliefweb.int/sites/reliefweb.int/files/resources/Science for DRM 2017_version 17 May 2017-compressed.pdf

32. Araya-Muñoz D, Metzger MJ, Stuart N, Wilson AMW, Alvarez L. Assessing urban adaptive capacity to climate change. J Environ Manage. 2016;183:314–24.

33. De Groeve T, Poljansek K, Vernaccini L. Index for Risk Management - INFORM. JRC Sci Policy Reports - Eur Comm [Internet]. 2015;96. Available from: https://www.google.it/webhp?sourceid=chrome-instant&ion=1&espv=2&ie=UTF-8#q=index+for+risk+management+INFORM+2015

34. Juhola S, Kruse S. A framework for analysing regional adaptive capacity assessments: challenges for methodology and policy making. Mitig Adapt Strateg Glob Chang [Internet]. 2015 [cited 2016 Nov 28];20(1):99–120. Available from: http://link.springer.com/article/10.1007/s11027-013-9481-z

35. Annoni P, Dijkstra L, Gargano N. The EU Regional Competitiveness Index 2016. 2017.

36. World Economic Forum. The Global Competitiveness Report 2017-2018 [Internet]. Geneva; 2017 [cited 2018 May 13]. Available from: https://www.weforum.org/reports/the-global-competitiveness-report-2017-2018

37. Sietchiping R. Applying an index of adaptive capacity to climate change in north-western Victoria, Australia. Appl GIS [Internet]. 2006 [cited 2016 Nov 28];2(3):1–16. Available from: http://www.epress.monash.edu/ag/ag060016.pdf

38. Bowen A, Cochrane S, Fankhauser S. Climate change, adaptation and economic growth. Clim Change [Internet]. Springer Netherlands; 2012 Jul 23 [cited 2018 May 15];113(2):95–106. Available from: http://link.springer.com/10.1007/s10584-011-0346-8

39. Tol RSJ, Yohe GW. The weakest link hypothesis for adaptive capacity: An empirical test. Glob Environ Chang [Internet]. Pergamon; 2007 May 1 [cited 2017 Sep 21];17(2):218–27. Available from: http://www.sciencedirect.com/science/article/pii/S0959378006000689

40. Barr R, Fankhauser S, Hamilton K. Adaptation investments: a resource allocation framework. Mitig Adapt Strateg Glob Chang [Internet]. Springer Netherlands; 2010 Dec 24 [cited 2018 May 17];15(8):843–58. Available from: http://link.springer.com/10.1007/s11027-010-9242-1

41. Smit B, Pilifosova O. Adaptation to climate change in the context of sustainable development and equity. Sustain Dev [Internet]. 2003 [cited 2017 Mar 21];8(9). Available from: http://www.start.org/Program/advanced_institute3_web/download/Smit_etal_IPCCwg2_ch18.pdf

42. Mitchell D, Myers M, Grant D. Land valuation: a key tool for disaster risk management. L Tenure J . 2014;1.

43. Roy F, Ferland Y. Land-use planning for disaster risk management. L tenure J. 2015;1.

44. Bakkensen LA, Fox-Lent C, Read LK, Linkov I. Validating Resilience and Vulnerability Indices in the Context of Natural Disasters. Risk Anal [Internet]. Wiley/Blackwell (10.1111); 2017 May [cited 2018 May 25];37(5):982–1004. Available from: http://doi.wiley.com/10.1111/risa.12677

45. Dipartimento delle Finanze. Statistiche sulle dichiarazioni [Internet]. 2018 [cited 2018 Aug 20]. Available from: http://www1.finanze.gov.it/finanze3/analisi_stat/index.php?search_class%5B0%5D=cCOMUNE&opendata=yes

46. Raffinetti E, Aimar F. GiniWegNeg: Computing the Gini-Based Coefficients for Weighted and Negative Attributes. R Packag version 101 [Internet]. 2016; Available from: https://cran.r-project.org/package=GiniWegNeg

47. Agenzia Entrate. Stock catastale [Internet]. 2013 [cited 2018 Aug 21]. Available from: https://www.agenziaentrate.gov.it/wps/content/Nsilib/Nsi/Schede/FabbricatiTerreni/omi/Banche+dati/Stock+catastale/?page=fabbricatiterreniimp

48. IPCC. Climate Change 2014: Impacts, Adaptation, and Vulnerability. Part A: Global and Sectoral Aspects. Contribution of Working Group II to the Fifth Assessment Report of the Intergovernmental Panel on Climate Change. In: Field CB, Barros, V.R. Dokken DJ, Mach KJ, Mastrandrea MD, Bilir, T.E Chatterjee M, Ebi KL, et al., editors. Cambridge, United Kingdom and New York, NY, USA: Cambridge University Press; 2014 [cited 2018 May 13]. p. 1132. Available from: https://www.google.com/search?q=Water+use+from+the+public+water+supply+as+a+share+of+the+water+input+to+a+distribution+network&ie=utf-8&oe=utf-8&client=firefox-b

49. Vallecillo S, Polce C, Barbosa A, Perpiña Castillo C, Vandecasteele I, Rusch GM, et al. Spatial alternatives for Green Infrastructure planning across the EU: An ecosystem service perspective. Landsc Urban Plan [Internet]. Elsevier; 2018 Jun 1 [cited 2018 May 13];174:41–54. Available from: https://www.sciencedirect.com/science/article/pii/S0169204618300707

50. Suckall N, Tompkins EL, Nicholls RJ, Kebede AS, Lázár AN, Hutton C, et al. A framework for identifying and selecting long term adaptation policy directions for deltas. Sci Total Environ [Internet]. Elsevier; 2018 Aug 15 [cited 2018 Aug 19];633:946–57. Available from: https://www.sciencedirect.com/science/article/pii/S0048969718309938

51. EEA. Nationally designated areas (CDDA) [Internet]. European Environment Agency. 2017 [cited 2018 Apr 8]. Available from: https://www.eea.europa.eu/data-and-maps/data/nationally-designated-areas-national-cdda-12

52. EEA. Natura 2000 data - the European network of protected sites [Internet]. European Environment Agency. 2017 [cited 2018 Apr 8]. Available from: https://www.eea.europa.eu/data-and-maps/data/natura-9

53. Copernicus. Green Linear Elements [Internet]. 2018 [cited 2018 Aug 20]. Available from: https://land.copernicus.eu/local/riparian-zones/green-linear-elements-gle-image?tab=mapview
