# Supplementary material for: Constructing a comprehensive disaster resilience index: The case of Italy
Source: PLoS One. 2019 Sep 16;14(9):e0221585. doi: 10.1371/journal.pone.0221585 (PMC6746365; doi:10.1371/journal.pone.0221585)
Supplement: S3 Appendix — (DOCX) [file pone.0221585.s003.docx]

**S3 Appendix: Data and Methodology**

**1. Data used and Statistical analysis**

## 1.1 Box-Cox Transformation

We have identified outliers based on skewness-kurtosis measures. Recent studies consider indicators with absolute skewness greater than 2.25 and kurtosis greater than 3.5 as problematic (Saisana et al., 2018). We adopted the Box-Cox transformation to adjust for outliers. Box-Cox transformations are continuous and are monotonously increasing generalized power transformations (including a logarithmic one as a special case), which were originally proposed by Box and Cox (1964) and defined as follows:

$x^{(\lambda)}= \left\{ \begin{aligned} \frac{x^{\lambda}-1}{\lambda} if \lambda\neq0 \\ \log\left( x \right) if \lambda=0 \end{aligned} \right.$ (Equation 1)

Depending on the power parameter $\lambda$ usually defined in the interval of [0,1], the transformation is the identity for $\lambda=1$, the logarithmic for $\lambda=0$ and the square root for $\lambda=0.5$ (Bergmeir et al., 2016; Bicego and Baldo, 2016; Lai, 2010; Proietti and Lütkepohl, 2013). Estimation of the optimal $\lambda$ has been argued in various statistical literature (e.g. Box and Cox (1964) and Sakia (1992)). We adopted the Box-Cox function based on maximization of the rank correlations proposed by Han (1987). We chose a $\lambda$ value corresponding to the maximum correlation coefficient of the transformed normal probability plot for each indicator (Han, 1987; NIST/SEMATECH, 2013). The analysis was conducted by using the R “ppcc” package (Pohlert, 2017). Data that have not been transformed due to out-of-range $\lambda$ values should either trimmed or winsorized. The indicators that have been transformed are listed in Table A, with the corresponding λ value used for transformation process. In all the cases listed in Table A, the transformation was successful in adjusting the skewness-kurtosis values and no subsequent trimming or winsorization were performed.

**Table A: Indicators transformed to correct the outliers**

| Indicators | Code | Transformation | $\boldsymbol{\lambda}$ |
| --- | --- | --- | --- |
| Quality rate of dwellings | HC_1 | Box-Cox | 0.2 |
| Rate of empty dwellings over total | HC_2 | Box-Cox | 0.1 |
| Index of overcrowded residences | HC_3 | Box-Cox | 0.6 |
| Index of old single parent families | COH_1 | Box-Cox | 0.2 |
| Index of elderly dependence | COH_4 | Box-Cox | 0.1 |
| Old age index | COH_5 | Box-Cox | 0.2 |
| Attraction index | COH_11 | Box-Cox | 0.2 |
| Illiteracy | EDU_1 | Box-Cox | 0.4 |
| Share of ecological corridors | ENV_2 | Box-Cox | 0.3 |
| Cadastral stock (property value) | RE_4 | Box-Cox | 0.1 |
| Share of families with potential economic hardship | RE_5 | Box-Cox | 0.4 |

The descriptive statistics of the final set of indictors containing the original and transformed ones, and density plots portraying the original and transformed Table A indicators are shown in Table B, Table C and Figure A.

**Table B. Descriptive statistics of the original variables**

| INDICATOR | MEAN | MEDIAN | MODE | STD. | VARIANCE | SKEWNESS | KURTOSIS | RANGE | MINIMUM | MAXIMUM |
| --- | --- | --- | --- | --- | --- | --- | --- | --- | --- | --- |
| ACC1_TT | 10.976 | 4.810 | 0.000 | 14.398 | 207.308 | 1.462 | 1.953 | 88.580 | 0.000 | 88.580 |
| ACC1_D | 12089.885 | 4154.340 | 0.000 | 17088.257 | 292008543.600 | 1.815 | 3.702 | 117902.050 | 0.000 | 117902.050 |
| ACC2_TT | 15.010 | 13.300 | 0.000 | 10.087 | 101.742 | 1.135 | 2.128 | 82.890 | 0.000 | 82.890 |
| ACC2_D | 15657.810 | 12873.450 | 0.000 | 12530.066 | 157002563.546 | 1.923 | 6.560 | 111083.730 | 0.000 | 111083.730 |
| INS_1 | 0.686 | 0.710 | 0.730 | 0.088 | 0.008 | -1.041 | 2.104 | 0.950 | 0.000 | 0.950 |
| HC_1 | 23.791 | 17.188 | 0.000 | 26.549 | 704.844 | 7.584 | 122.527 | 657.265 | 0.000 | 657.265 |
| HC_2 | 70.108 | 33.500 | 15.600 | 118.311 | 13997.435 | 6.430 | 72.850 | 2630.000 | 0.000 | 2630.000 |
| HC_3 | 1.003 | 0.800 | 0.000 | 0.989 | 0.978 | 2.684 | 17.245 | 16.700 | 0.000 | 16.700 |
| HC_4 | 85.568 | 86.996 | 100.000 | 8.021 | 64.331 | -1.525 | 4.545 | 76.874 | 23.126 | 100.000 |
| COH_1 | 10.210 | 9.731 | 9.091 | 7.762 | 60.246 | 27.783 | 992.263 | 354.839 | 0.000 | 354.839 |
| COH_2 | 8.434 | 7.250 | 0.000 | 6.125 | 37.516 | 2.041 | 10.228 | 76.800 | 0.000 | 76.800 |
| COH_3 | 33.014 | 31.157 | 33.333 | 8.979 | 80.626 | 1.222 | 2.304 | 74.310 | 11.232 | 85.542 |
| COH_4 | 36.012 | 33.800 | 31.000 | 12.281 | 150.816 | 2.375 | 13.834 | 170.600 | 7.500 | 178.100 |
| COH_5 | 195.672 | 166.667 | 200.000 | 141.870 | 20127.143 | 7.085 | 86.577 | 2850.000 | 0.000 | 2850.000 |
| COH_6 | 20.366 | 20.587 | 16.667 | 3.852 | 14.835 | -0.374 | 1.200 | 38.725 | 0.000 | 38.725 |
| COH_7 | 3.219 | 3.000 | 2.800 | 1.325 | 1.756 | 1.113 | 3.054 | 13.800 | 0.000 | 13.800 |
| COH_8 | 40.514 | 41.200 | 44.800 | 7.601 | 57.776 | -0.251 | -0.180 | 61.300 | 10.400 | 71.700 |
| COH_9 | 35.219 | 36.100 | 42.500 | 12.557 | 157.684 | -0.187 | -0.684 | 71.700 | 1.500 | 73.200 |
| COH_10 | 19.127 | 18.644 | 20.000 | 7.805 | 60.918 | 0.448 | 0.354 | 56.961 | 0.000 | 56.961 |
| COH_11 | 34.501 | 31.613 | 28.989 | 17.678 | 312.517 | 2.930 | 32.328 | 374.795 | 1.205 | 376.000 |
| EDU_1 | 1.197 | 0.600 | 0.300 | 1.427 | 2.036 | 2.587 | 9.241 | 15.000 | 0.000 | 15.000 |
| EDU_2 | 22.369 | 21.689 | 22.222 | 4.757 | 22.630 | 1.332 | 4.061 | 49.857 | 7.143 | 57.000 |
| EDU_3 | 7.274 | 6.910 | 10.300 | 2.702 | 7.300 | 1.301 | 4.013 | 27.500 | 0.000 | 27.500 |
| ENV_1 | 25.907 | 11.793 | 0.000 | 31.043 | 963.677 | 1.012 | -0.240 | 100.000 | 0.000 | 100.000 |
| ENV_2 | 0.078 | 0.021 | 0.000 | 0.181 | 0.033 | 8.902 | 139.312 | 4.247 | 0.000 | 4.247 |
| RE_1 | 10752.159 | 11177.612 | 7682.320 | 3185.622 | 10148185.172 | 0.102 | 0.054 | 30508.068 | 2076.525 | 32584.593 |
| RE_2 | 0.266 | 0.305 | 0.315 | 0.083 | 0.007 | -1.050 | -0.162 | 0.431 | 0.015 | 0.446 |
| RE_3 | 10.127 | 7.700 | 6.200 | 6.306 | 39.765 | 1.211 | 1.023 | 42.200 | 0.000 | 42.200 |
| RE_4 | 4573.495 | 1274.910 | 67.160 | 40204.088 | 1616368669.666 | 62.223 | 4672.308 | 3142883.140 | 25.110 | 3142908.250 |
| RE_5 | 2.012 | 1.300 | 1.000 | 1.862 | 3.467 | 2.429 | 9.228 | 17.900 | 0.000 | 17.900 |

**Table C. Descriptive statistics of the data set containing transformed variables (Box-Cox)**

| INDICATOR | MEAN | MEDIAN | MODE | STD. | VARIANCE | SKEWNESS | KURTOSIS | RANGE | MINIMUM | MAXIMUM |
| --- | --- | --- | --- | --- | --- | --- | --- | --- | --- | --- |
| ACC1_TT | 10.976 | 4.810 | 0.000 | 14.398 | 207.308 | 1.462 | 1.953 | 88.580 | 0.000 | 88.580 |
| ACC1_D | 12089.885 | 4154.340 | 0.000 | 17088.257 | 292008543.600 | 1.815 | 3.702 | 117902.050 | 0.000 | 117902.050 |
| ACC2_TT | 15.011 | 13.300 | 0.000 | 10.087 | 101.742 | 1.135 | 2.128 | 82.890 | 0.000 | 82.890 |
| ACC2_D | 15657.810 | 12873.450 | 0.000 | 12530.066 | 157002563.546 | 1.923 | 6.560 | 111083.730 | 0.000 | 111083.730 |
| INS_1 | 0.686 | 0.710 | 0.730 | 0.088 | 0.008 | -1.041 | 2.104 | 0.950 | 0.000 | 0.950 |
| HC_1_t | 3.897 | 3.831 | -5.000 | 1.559 | 2.432 | -0.034 | 3.305 | 18.303 | -5.000 | 13.303 |
| HC_2_t | 4.387 | 4.207 | 3.162 | 1.668 | 2.783 | 0.261 | 2.358 | 21.978 | -10.000 | 11.978 |
| HC_3_t | -0.209 | -0.209 | -1.667 | 0.973 | 0.947 | 0.507 | 1.311 | 9.026 | -1.667 | 7.359 |
| HC_4 | 85.568 | 86.996 | 100.000 | 8.021 | 64.331 | -1.525 | 4.545 | 76.874 | 23.126 | 100.000 |
| COH_1_t | 2.882 | 2.881 | 2.775 | 0.492 | 0.242 | 2.023 | 57.363 | 16.180 | -5.000 | 11.180 |
| COH_2 | 8.434 | 7.250 | 0.000 | 6.125 | 37.516 | 2.041 | 10.228 | 76.800 | 0.000 | 76.800 |
| COH_3 | 33.014 | 31.157 | 33.333 | 8.979 | 80.626 | 1.222 | 2.304 | 74.310 | 11.232 | 85.542 |
| COH_4_t | 4.248 | 4.220 | 4.097 | 0.435 | 0.189 | 0.495 | 1.532 | 4.558 | 2.232 | 6.791 |
| COH_5_t | 9.059 | 8.910 | 9.427 | 1.384 | 1.914 | 1.225 | 6.398 | 24.544 | -5.000 | 19.544 |
| COH_6 | 20.366 | 20.587 | 16.667 | 3.852 | 14.835 | -0.374 | 1.200 | 38.725 | 0.000 | 38.725 |
| COH_7 | 3.219 | 3.000 | 2.800 | 1.325 | 1.756 | 1.113 | 3.054 | 13.800 | 0.000 | 13.800 |
| COH_8 | 40.514 | 41.200 | 44.800 | 7.601 | 57.776 | -0.251 | -0.180 | 61.300 | 10.400 | 71.700 |
| COH_9 | 35.219 | 36.100 | 42.500 | 12.557 | 157.684 | -0.187 | -0.684 | 71.700 | 1.500 | 73.200 |
| COH_10 | 19.128 | 18.644 | 20.000 | 7.805 | 60.918 | 0.448 | 0.354 | 56.961 | 0.000 | 56.961 |
| COH_11_t | 4.963 | 4.976 | 4.804 | 0.981 | 0.963 | -0.066 | 1.080 | 11.178 | 0.190 | 11.368 |
| EDU_1_t | -0.188 | -0.462 | -0.955 | 1.127 | 1.270 | 0.515 | 0.674 | 7.385 | -2.500 | 4.885 |
| EDU_2 | 22.369 | 21.689 | 22.222 | 4.757 | 22.630 | 1.332 | 4.061 | 49.857 | 7.143 | 57.000 |
| EDU_3 | 7.274 | 6.910 | 10.300 | 2.702 | 7.300 | 1.301 | 4.013 | 27.500 | 0.000 | 27.500 |
| ENV_1 | 25.907 | 11.793 | 0.000 | 31.043 | 963.677 | 1.012 | -0.240 | 100.000 | 0.000 | 100.000 |
| ENV_2_t | -2.349 | -2.287 | -3.333 | 0.851 | 0.725 | 0.410 | -0.376 | 5.144 | -3.333 | 1.811 |
| RE_1 | 10752.159 | 11177.612 | 7682.320 | 3185.622 | 10148185.172 | 0.102 | 0.054 | 30508.068 | 2076.525 | 32584.593 |
| RE_2 | 0.266 | 0.305 | 0.315 | 0.083 | 0.007 | -1.050 | -0.162 | 0.431 | 0.015 | 0.446 |
| RE_3 | 10.127 | 7.700 | 6.200 | 6.306 | 39.765 | 1.211 | 1.023 | 42.200 | 0.000 | 42.200 |
| RE_4_t | 10.758 | 10.443 | 5.230 | 2.907 | 8.450 | 0.860 | 2.042 | 30.838 | 3.803 | 34.641 |
| RE_5_t | 0.510 | 0.277 | 0.000 | 1.132 | 1.281 | 0.212 | 1.317 | 7.926 | -2.500 | 5.426 |

**Figure A. Density graphs (standardized) for original and transformed variables**

| **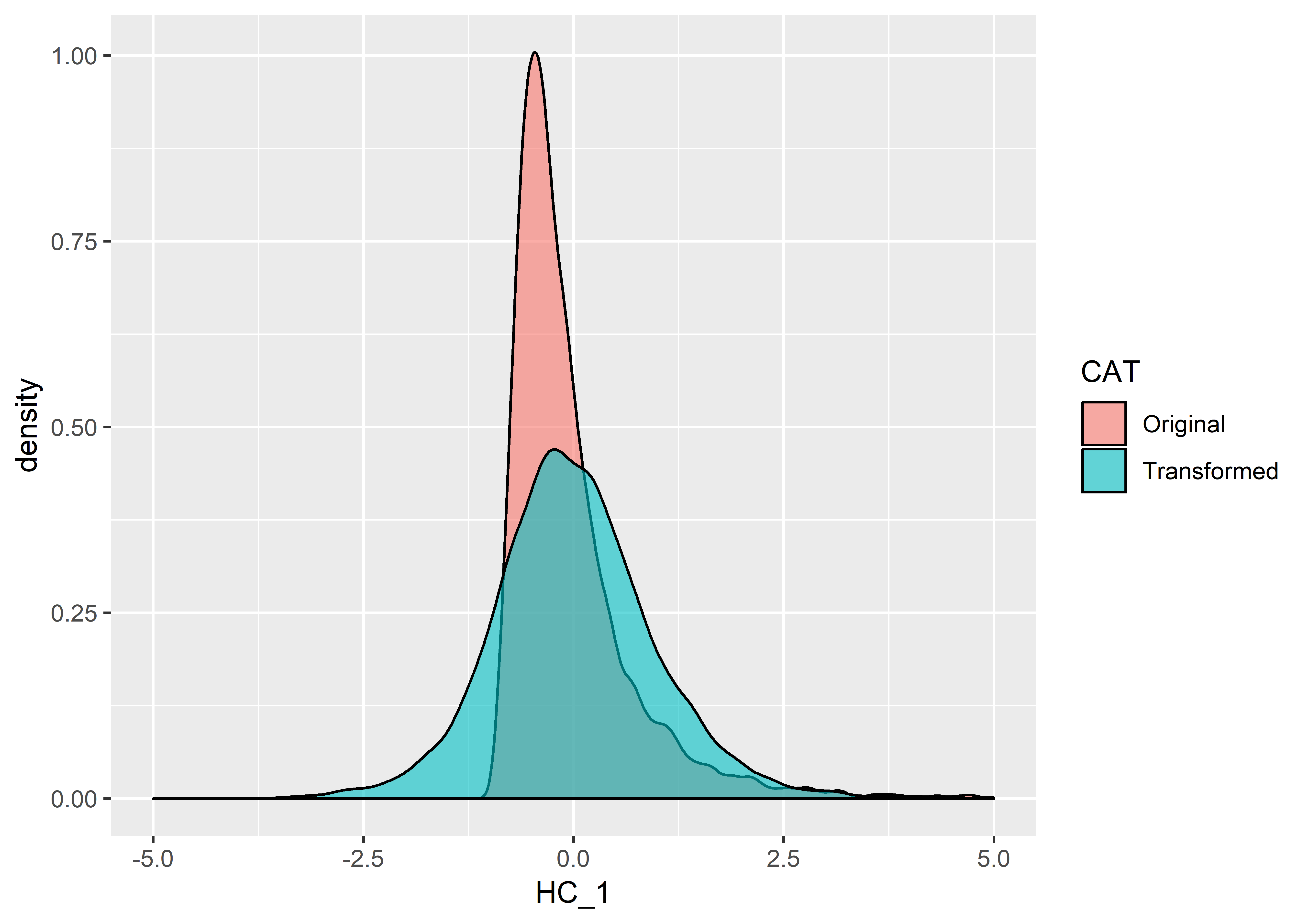** | **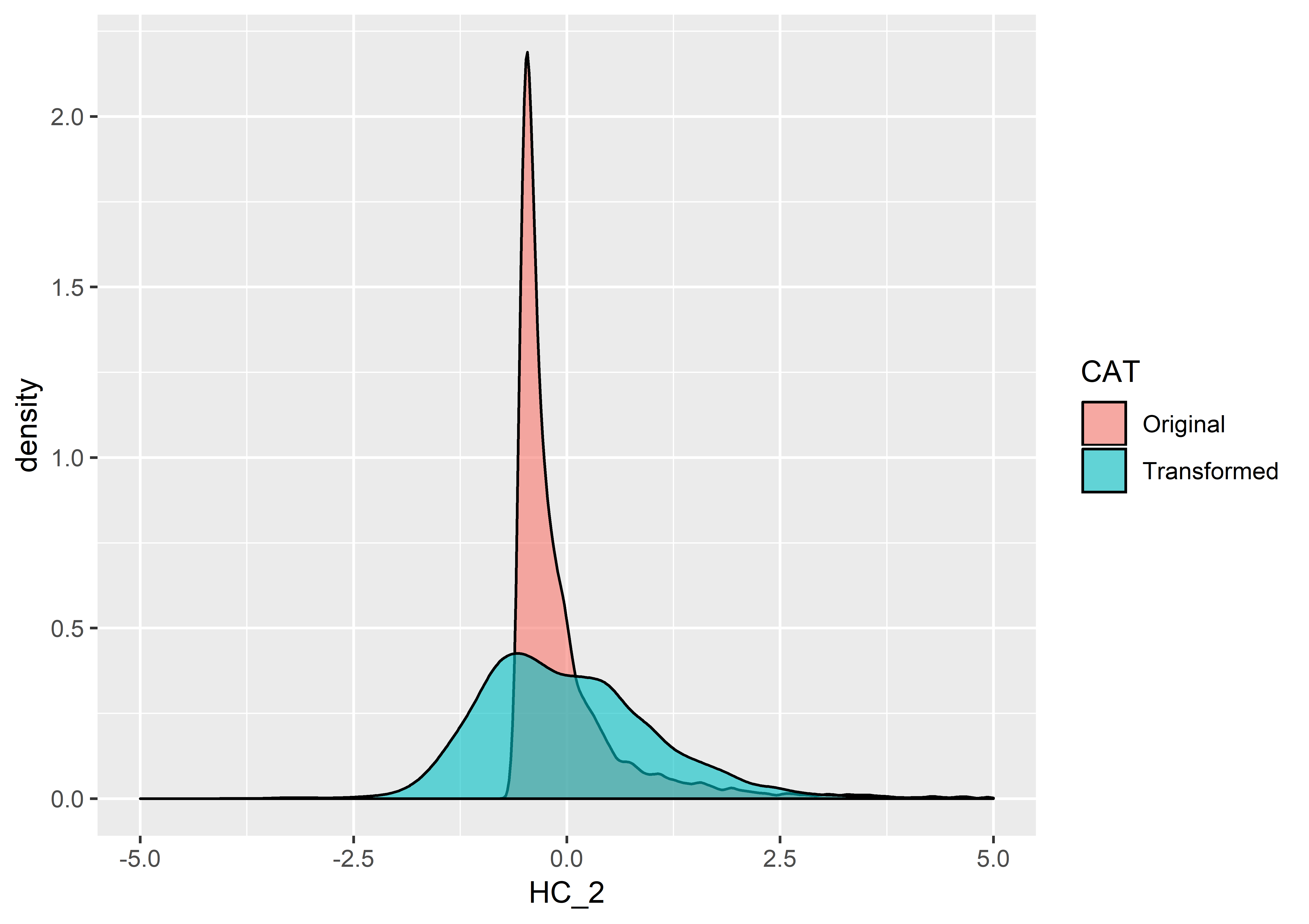** |
| --- | --- |
| **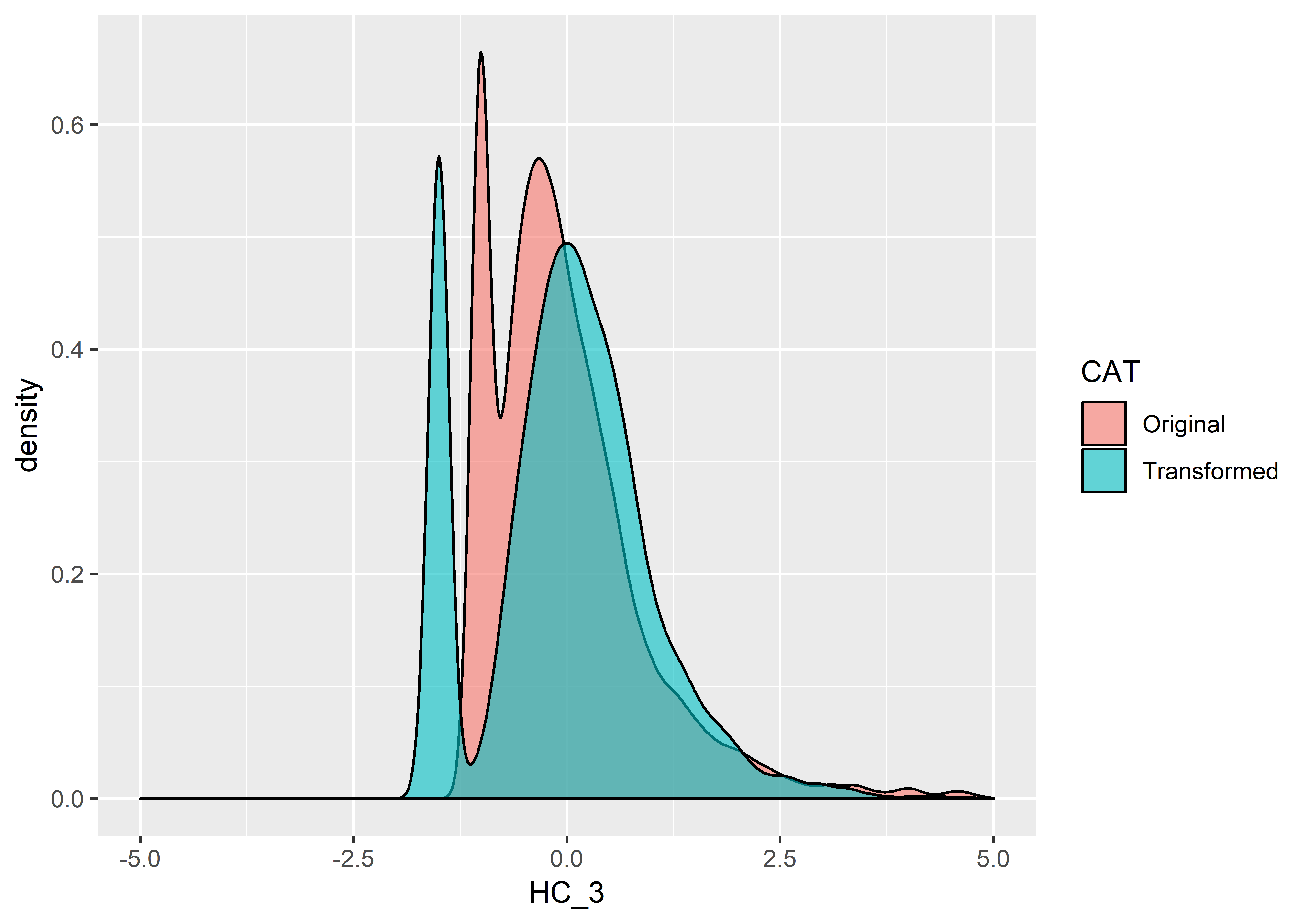** | **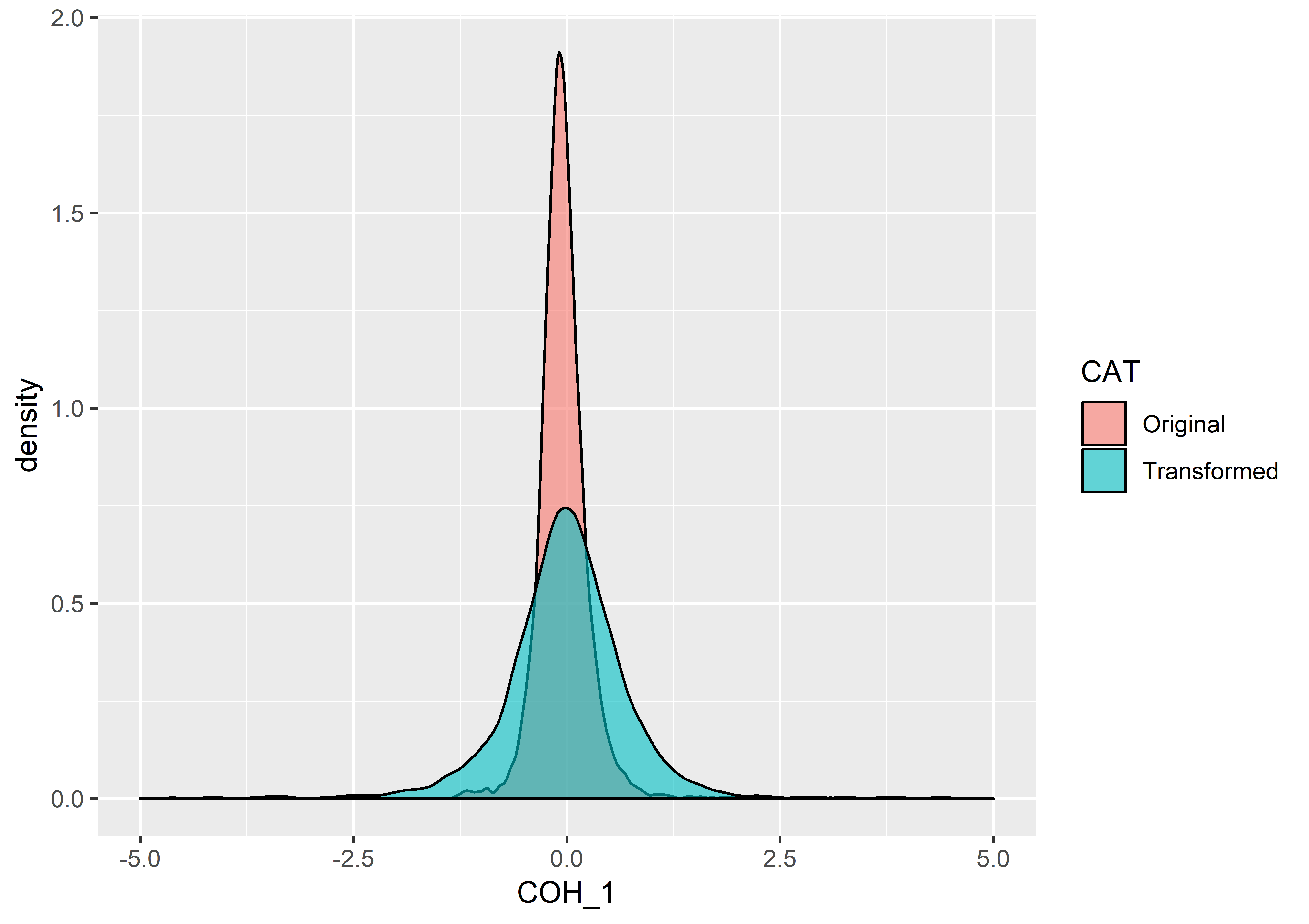** |
| **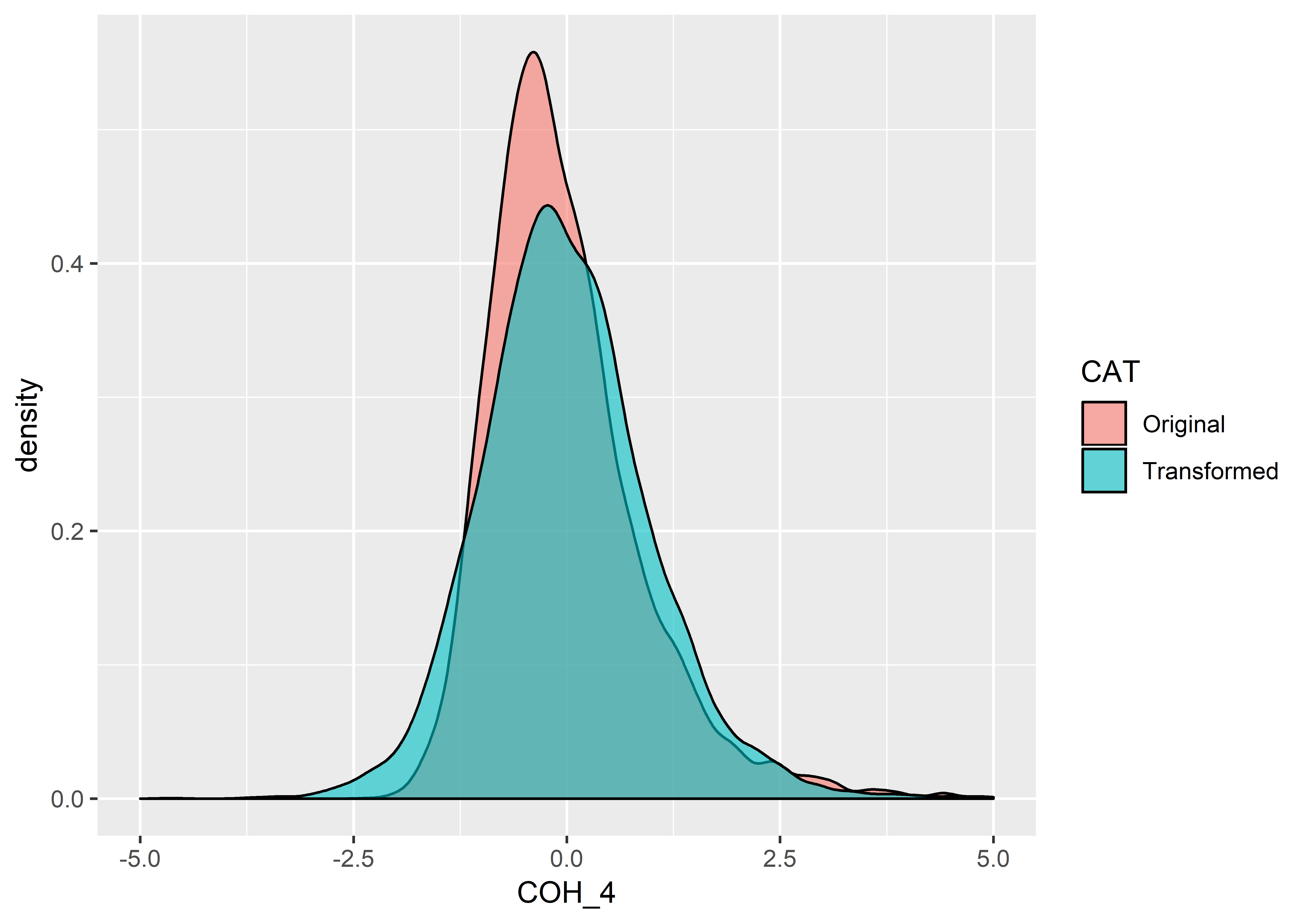** | **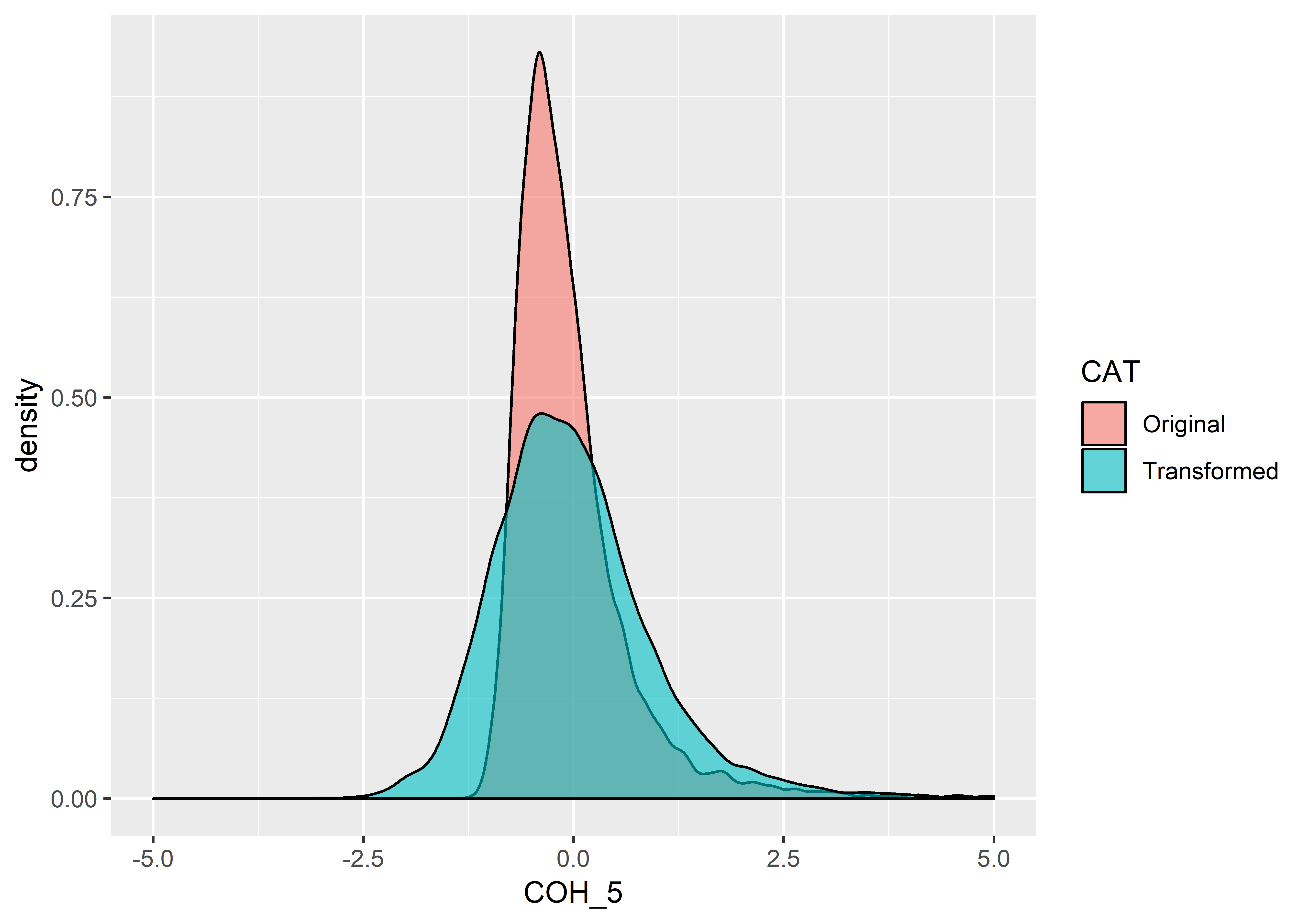** |
| **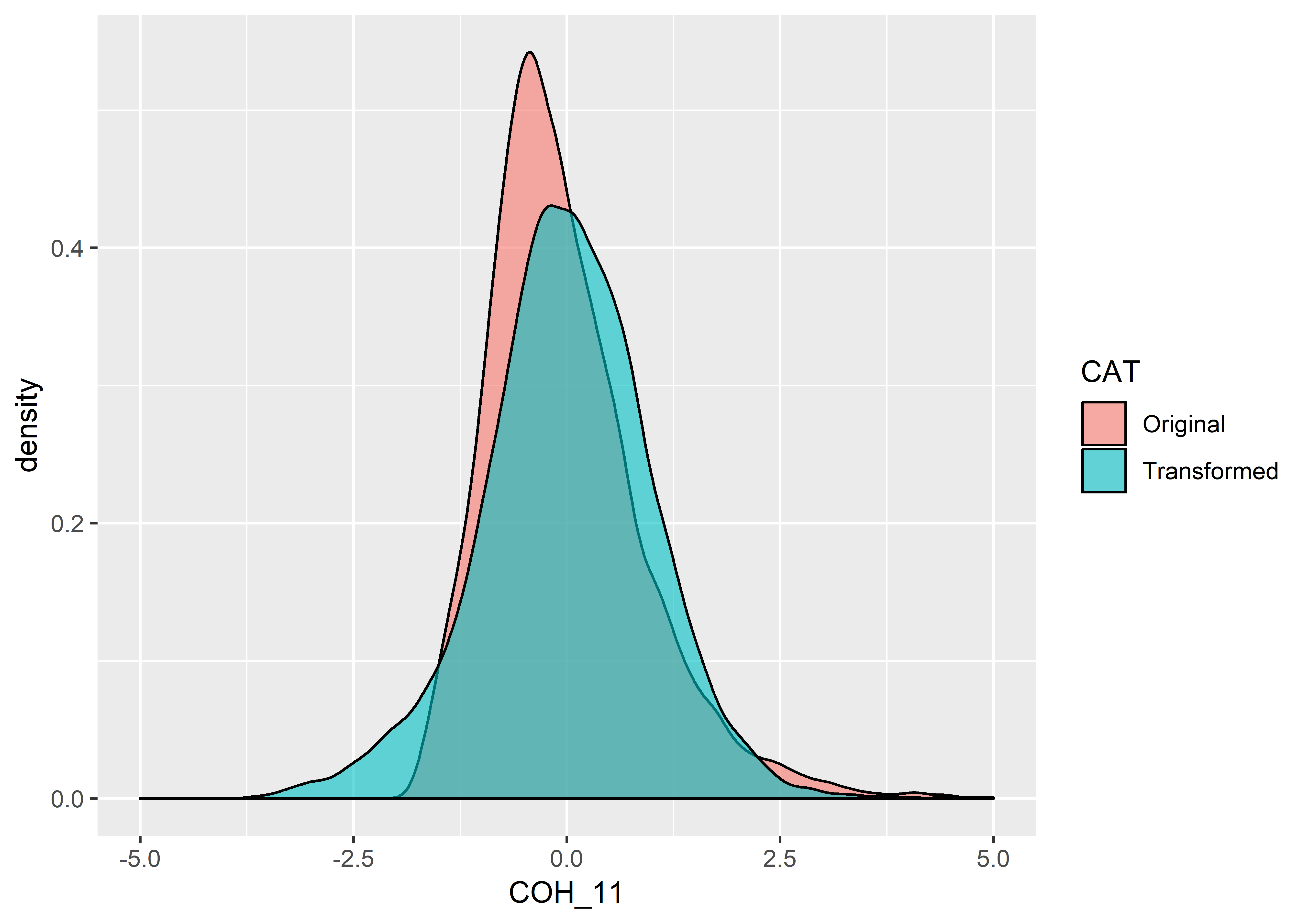** | **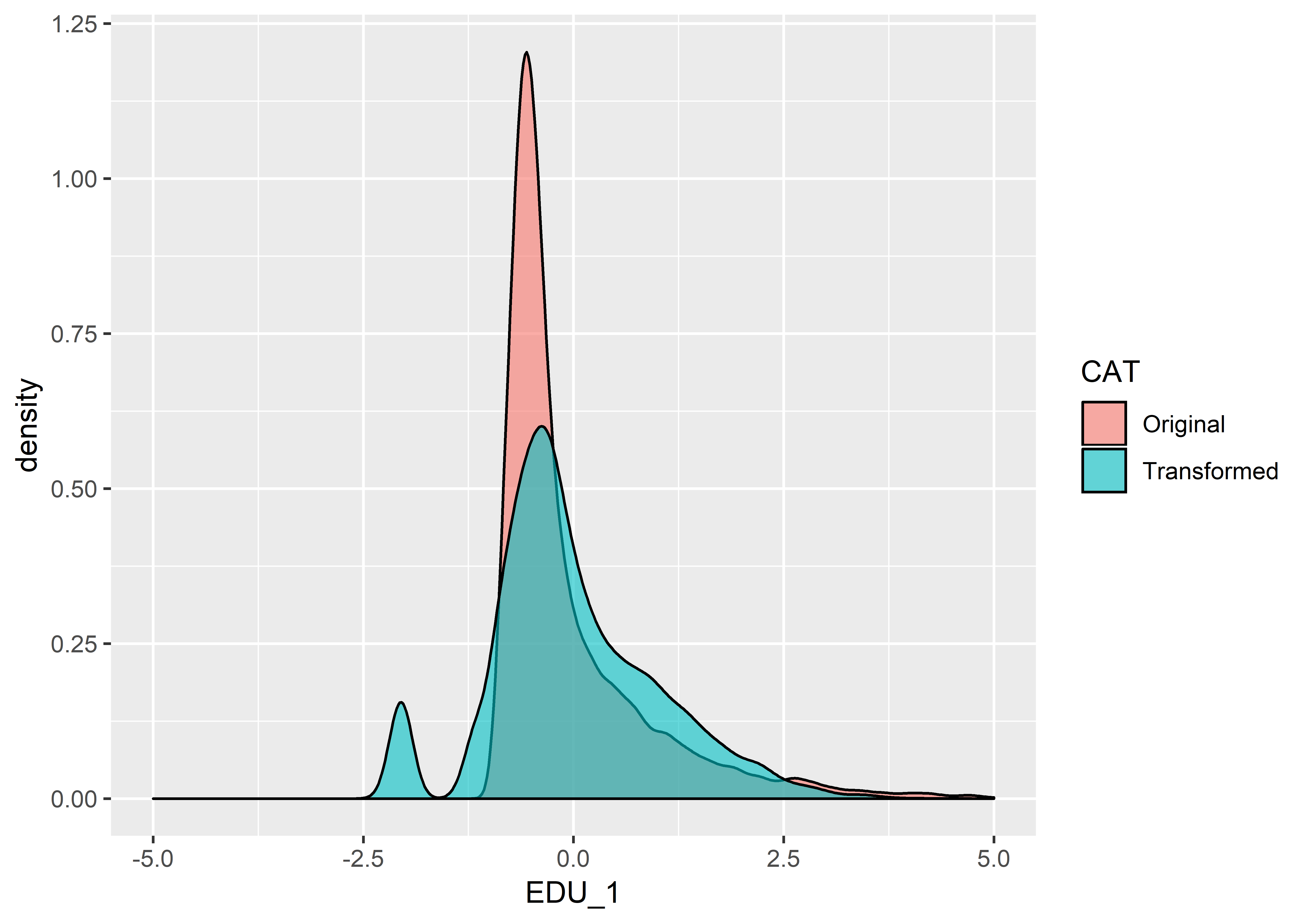** |
| **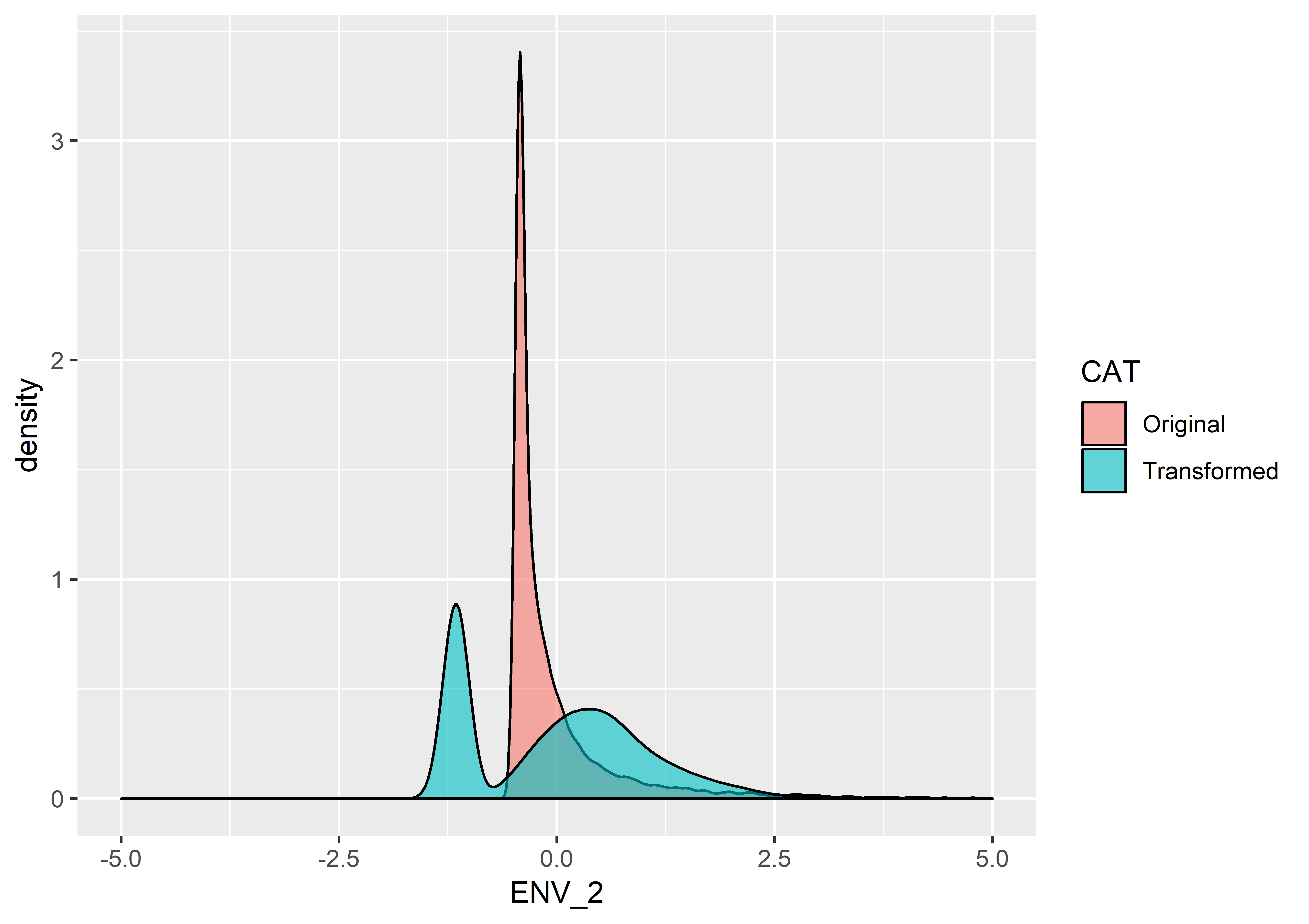** | **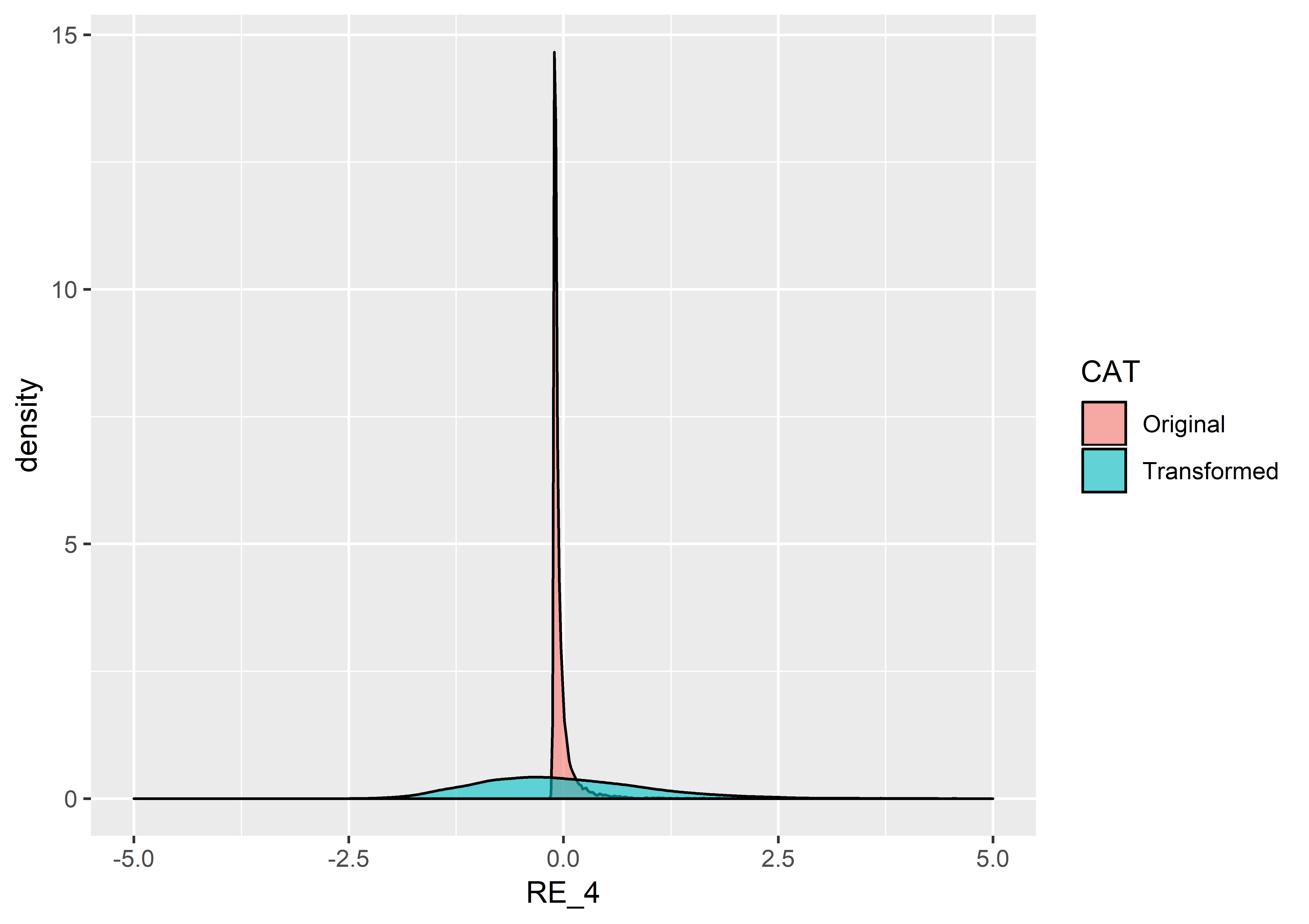** |
| **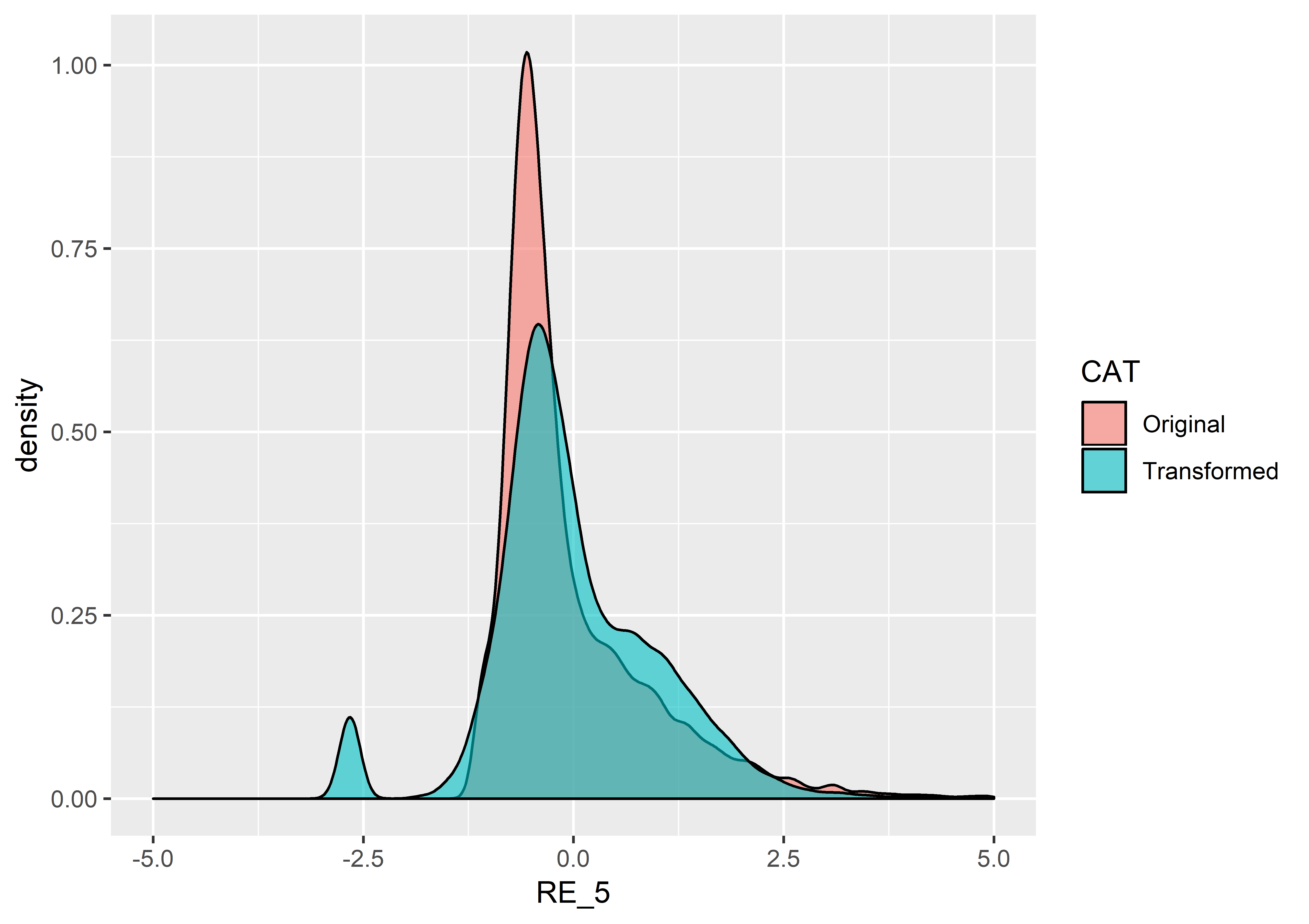** |  |

## 1.2 Multicollinearity test

Multicollinearity of the data was performed to avoid high intercorrelation. When multicollinearity exceeds a certain threshold, standard errors and variances are inflated, possibly biasing the overall results (Avkiran and Ringle, 2018; OECD, 2008). In order to detect multicollinearity among the variables, we calculated the Variance Inflation Factor (VIF), defined as:

${VIF}_{i}=\frac{1}{(1-R_{i}^{2})}$ (Equation 2)

where $R_{i}^{2}$ is the proportion of variance of indicator *i* associated with the other indicators in the data set (Avkiran and Ringle, 2018; Makoka, 2008). Various VIF threshold values have been considered for the collinearity test (Hagenlocher et al., 2016; KC et al., 2015; Makoka, 2008; OECD, 2008). Researchers working on risk, vulnerability and resilience have been considering VIF = 10 as the cut-off value (Frigerio and De Amicis, 2016; Makoka, 2008). We calculated the VIF values using the “olsrr” package in R (Hebbali, 2018) in an iterative process considering each indicator as a dependent variable and the rest as independent variables. After performing the multicollinearity test, travel time indicators (ACC1_TT and ACC2_TT), old age index (COH_5) and containment index (COH_10) were excluded from the analysis. The choice of excluding COH_10 instead of COH_9 (commuting rate) has been made with regard to previously conducted indices available in the literature. Table D displays the VIF values for the first analysis cycle before and after elimination of the problematic indicators.

**Table D. Multicollinearity statistics**

| INDICATOR | CODE | VIF (before) | VIF (after) |
| --- | --- | --- | --- |
| Travel time to service centers | ACC1_TT | 1.881 | excluded |
| Travel time to fire brigades | ACC2_TT | 16.081 | excluded |
| Distance to fire brigades | ACC2_D | 15.468 | 1.262 |
| electection participation | INS_1 | 3.487 | 3.416 |
| Quality rate of dwellings | HC_1_t | 1.158 | 1.154 |
| rate of empty dwellings over total | HC_2_t | 2.59 | 2.533 |
| Index of overcrowded residences | HC_3_t | 1.368 | 1.349 |
| residential buildings over total | HC_4 | 1.088 | 1.08 |
| Index of single parent families | COH_1_t | 1.098 | 1.092 |
| Index of large families | COH_2 | 1.743 | 1.68 |
| Index of small families | COH_3 | 4.763 | 4.431 |
| Index of elderly dependence | COH_4_t | 13.641 | 6.735 |
| Old age index | COH_5_t | 21.322 | excluded |
| Index of minor dependence | COH_6 | 6.156 | 2.25 |
| Share of the families with assistance need | COH_7 | 2.473 | 2.467 |
| Participation in the labor market - female | COH_8 | 5.054 | 4.248 |
| commuting rate for study or work | COH_9 | 16.011 | 4.43 |
| containment index | COH_10 | 12.03 | excluded |
| attraction index | COH_11_t | 2.745 | 2.433 |
| illiteracy | EDU_1_t | 3.135 | 3.132 |
| Low education index | EDU_2 | 3.892 | 3.814 |
| High education index | EDU_3 | 2.399 | 2.348 |
| Share of the protected lands | ENV_1 | 1.144 | 1.137 |
| Share of ecological coridors | ENV_2_t | 1.132 | 1.123 |
| income | RE_1 | 7.064 | 7.01 |
| GINI index | RE_2 | 4.248 | 4.166 |
| Unemployment rate | RE_3 | 4.051 | 3.72 |
| cadastral stock (property value) | RE_4_t | 2.828 | 2.637 |
| Share of the families with potential economic hardship | RE_5_t | 3.707 | 3.668 |
| Dependent Variable: ACC1_D |  |  |  |

## 2 Analysis

To allow for the intercomparing of different indicators, the selected indicators are (OECD, 2008). In order to analyse how different normalization procedures can affect the final results of index composition, we evaluated three types of normalization methods, namely Adjusted Mazziotta-Pareto (AMP), Topsis, and z-score standardization. Since the AMP normalization technique has been used in the social vulnerability index provided by ISTAT, it is considered as the baseline in our analysis. The three types of normalization techniques are described below:

The AMP normalization is given by:

$r_{ij}=\frac{{(x}_{ij}-{Min}_{xj})}{{(Max}_{xj}-{Min}_{xj})}60+70$ (Equation 3)

where $x_{ij}$ is the value of the indicator j for the municipality *i* and ${Min}_{xj}$ and ${Max}_{xj}$ are the goalposts for the indicator j. To revert the scales of indicators with negative polarity, the complement of Equation 3 with respect to 200 has been calculated (Lucarelli et al., 2014; Mazziotta and Pareto, 2014). The ‘polarity’ of an individual indicator is the sign of the relation between the indicator and the phenomenon to be measured (in our case resilience). Some indicators may be positively correlated with the phenomenon to be measured (positive ‘polarity’), whereas others may be negatively correlated with it (negative ‘polarity’). We wanted to normalize the indicators so that an increase in the normalized indicators corresponds to increase in the composite resilience index. Before aggregation, the indicators correspond to decrease in resilience (negative polarity) should be somehow reverted to positive polarity.

The Topsis normalization takes into account the shortest distance from the positive benchmark for the best alternative the farthest distance from the negative benchmark. Given the matrix $X=\left\{ x_{\mathrm{ij}} \right\}$ with m rows (municipalities) and n columns (indicators), the normalized matrix can be computed as follows:

$r_{ij}=\frac{x_{ij}}{\sqrt{\sum_{i=1}^{m} {r_{ij}}^{2}}} ;j=1,2,\ldots, n;i=1,2,\ldots., m$ (Equation 4)

The scales were reverted by using the complement of the Equation 4 (1- $r_{ij}$) for the indicators with negative polarity (Biswas et al., 2016; Shanian and Savadogo, 2006).

Finally, z-scores are one of the most common standardization methods and preserve range (maximum and minimum) and introduce the dispersion of the series (standard deviation / variance). The formula is:

$r_{ij}=\frac{x_{ij}-M_{xj}}{S_{xj}}$ (Equation 5)

where the $M_{xj}$ and $S_{xj}$ are the mean and standard deviation of indicator *j*. To revert the scales of indicators with negative polarity, Equation 1 has been multiplied by -1 (Maggino, 2017).

In order to compare the results with the social vulnerability index, we first construct the resilience index by using the Adjusted Mazziotta-Pareto method. Denoting with $M_{r_{i}}$ and $S_{r_{i}}$, respectively, the mean and the standard deviation of the normalized values of unit *i*, the generalized form of the adjusted MPI can be computed by using the following formula:

${AMPI}^{+/-}=M_{r_{i}}\pm S_{r_{i}}{cv}_{i}$ (Equation 6) where ${cv}_{i}=\frac{S_{r_{i}}}{M_{r_{i}}}$ is the variation coefficient of unit i and the ± sign depends on the kind of phenomenon to be measured (Lucarelli et al., 2014; Mazziotta and Pareto, 2014). Since the increasing values of the index correspond to an improvement of resilience, a downward penalization has been used (${AMPI}^{-})$ (Maggino, 2017).

AMPI is a hybrid, non-compensatory aggregation method penalising the compensability among indicators in order to incorporate the possible trade-offs between higher performance in some indicators and lower performance in other ones. Using additive aggregators with high degree of compensation implies that underperformance with respect to one or more indicators may not receive the adequate attention. Hence, the compensability can be penalised to achieve an optimum estimation of the aggregated index. Nevertheless, the choice of aggregator with intended degree of compensation should be made with respect to the context and scope of the analysis and expert judgements (Aggarwal, 2015; Fernandez et al., 2017; Langhans et al., 2014; Liu et al., 2014). In the AMPI, the penalization is addressed by subtracting a component (${cv}_{i}$) from a non-weighted arithmetic mean (subtraction in the case of the resilience index) (Greco et al., 2018). Nevertheless, by using AMPI, the degree of penalization is not explicit and trade-offs among the indicators cannot be clearly portrayed in terms of degree of compensation. To unequivocally display the trade-offs with respect to compensability, a spectrum of hybrid methods can be deployed, such as Fuzzy Gamma, Mean-Min function, generalized mean, etc. Since, we are simultaneously incorporating various normalization procedures as part of the sensitivity analysis, the aggregation must be independent from the type of normalization. To control the trade-offs during the aggregation, we applied the ordered weighted average (OWA) operator introduced by Yager (1988) which provides a circumstance in which the degree of compensation can be adjusted and modified. The OWA operator is defined as follows:

$OWA\left( x_{1},\ldots,x_{n} \right)=\sum_{i=1}^{n} w_{i}.x_{\sigma(i)}$ (Equation 7)

where $\sigma$ is a permutation ordering the elements as $x_{\sigma(1)}\leq\ldots\leq x_{\sigma(n)}$, with associated non-negative weights in the range of [0,1] summing up to one ($\sum_{i=1}^{n} w_{i}=1$ ) (Jin et al., 2017; Yager, 1988; Zabeo, 2011). The OWA operator provides a family of operators, including a maximum (1,0, 0,…,0), minimum (0,0,…,1), k-order statistics (kth weight equal to 1 and the rest zero), the arithmetic mean ($\frac{1}{n}$,$\frac{1}{n}$…,$\frac{1}{n}$) and a window type OWA, which takes the average of m components in the center (Fullér, 1996; Zabeo, 2011).

The weights can be ordered in different ways and distributed, by using either linear or uniform patterns, as graphically depicted in Figure B (Jin et al., 2017; Mysiak et al., 2018). In order to evaluate how different weight distributions can affect OWA, different combinations of weights have been simulated, following either a linear or uniform distribution. In total, 128 different weight combinations have been tested, 65 of which follow a linear function distribution, while the remaining 63 follow uniform weight distribution patterns. For the 65 weight combinations following a linear function, 26 result from descending linear functions (an example shown in the top left of Figure B), 13 from central linear distributions (an example shown in the top middle of Figure B), and another 26 of ascending linear functions (an example shown in the top right of Figure B). Similarly, For the 63 weight combinations following a uniform distribution, 25 consist of left-side biased distributions (an example shown in the bottom left of Figure B), 13 of central uniform distributions (example shown in bottom middle of Figure B), and another 25 of right-side biased distributions (example shown in bottom right of Figure B).

| **Linear Weight Function** |  |  |
| --- | --- | --- |
|  |  |  |
| **Uniform Weight Function** |  |  |
|  |  |  |

**Figure B. Example of six different distributions of OWA weights. Top part: Linear weight function. Bottom part: Uniform weight function.**

In order to examine the trade-offs, Yager (1988) introduced the degree of ORNESS determining the proximity to the maximum operator for a particular set of weights (Chaji et al., 2018; Zabeo, 2011). The ORNESS index is given by:

$ORNESS\left( w_{1},\ldots,w_{n} \right)=\frac{1}{n-1}\sum_{i=1}^{n} w_{i}.(n-i)$ (Equation 8)

The ORNESS index evaluates the extent to which the indicators compensate each other. The ORNESS equal to 1 shows the highest proximity to a maximum operator indicating full compensative trade-offs (optimistic approach). Contrarily, ORNESS equal to zero indicates the highest propensity to a minimum operator reflecting perfect complementary behaviour (pessimistic approach). The special case of ORNESS equal to 0.5 determines the highest proximity to an average (arithmetic mean) operator (additive approach) (Pinar et al., 2014). The ANDNESS index is also introduced as the complement of the ORNESS ($ANDNESS+ORNESS=$1), and measures the level of complementarity among the indicators (Belles-Sampera et al., 2014; Dujmović and Cordeliers, 2006; Pinar et al., 2014). Figure C shows the ORNESS trends by using the 128 different combinations of weights for both linear and uniform patterns. The OWA operator controls the level of compensation by using a different order of weights. The order of weights corresponding to higher ORNESS levels indicates a higher degree of compensation and proximity to a maximum operator and vice versa.

**

**

**Figure C.** **Degree of ORNESS following a Linear (left), and a Uniform (right) distribution of OWA weights for all 128 different weight distributions.**

We use the designed combinations to perform sensitivity and robustness analysis on a resilience index. The sensitivity-robustness analysis is performed by considering the different normalization and aggregation procedures. To this end, we analyze the resilience data normalized with three different methods, using various combinations of OWA weights (both linear and uniform distributions) reflecting the ORNESS in the range of [0,1]. In addition, the original data (i.e. the data that feeds the transformation procedure) is also considered in order to identify possible effects of the Box-Cox transformation. In order to improve the end-user applicability and extract robust rankings out of various OWA configurations, we employ the relative dominance measure($\rho)$ proposed by Pinar et al. (2014), which indicates the extent of relative dominance of the i^th^ administrative unit across simulations (the derivation procedure is explained in the S4 Appendix). The $\rho$ measure takes into account the relationship between administrative units across the simulated combinations in order to investigate to what extent each unit either dominates or is being dominated by other units by considering the overall variability in the resilience results imposed by diverse inputs.

## 3 Dominance analysis

The following formulas have been employed to calculate the relative dominance measure comparing countries *i* and *j* administrative units (municipalities) included in the ranking (explained in 2.3 section in the manuscript). First of all, the “average cardinal dominance of municipality *i* on municipality *j* has to be calculated which is given by:

$\Delta\left( i,j \right)=\frac{1}{N}\sum_{k=1}^{K} F[R_{k}\left( i \right)-R_{k}\left( j \right)]$ (Equation 9)

where *N* is the number of municipalities considered in the analysis, *K* represents the number of models (OWA combinations with various normalized data) generated for the analysis, $R(i)$ and $R(j)$ are the CDRI scores for *i ^th^ and j ^th^* countries respectively, and $F(x)$ is given by:

$F\left( x \right)=\left\{ \begin{aligned} 0 if x<0 \\ x if x\geq0 \end{aligned} \right.$ (Equation 10)

The “average cardinal dominance shows the extent the *i ^th^* municipality dominates the *j ^th^* municipality on average*.* Afterwards, the total dominance measure of municipality *i* on every other municipality can be computed as follows:

$\rho^{+}\left( i \right)=\frac{1}{N-1}\sum_{j=1}^{N} \Delta\left( i,j \right)$ (Equation 11)

The degree to which municipality *i* is dominated by every other municipality is given by:

$\rho^{-}\left( i \right)=\frac{1}{N-1}\sum_{j=1}^{N} \Delta\left( j,i \right)$ (Equation 12)

Finally, the relative dominance measure can be calculated as follows:

$\rho\left( i \right)=\frac{\rho^{+}\left( i \right)}{\rho^{+}\left( i \right)+\rho^{-}\left( i \right)}$ (Equation 13)

This measure is defined within the range of [0,1] where measure 1 indicates the municipality *i* fully dominates all other municipalities and measure 0 depicts that municipality *i* has been thoroughly dominated by all other municipalities.

**References**

Aggarwal, M., 2015. Compensative weighted averaging aggregation operators. Appl. Soft Comput. 28, 368–378. https://doi.org/10.1016/J.ASOC.2014.09.049

Avkiran, N.K., Ringle, C.M., 2018. Partial Least Squares Structural Equation Modeling : Recent Advances in Banking and Finance. Springer, Cham, Switzerland. https://doi.org/10.1007/978-3-319-71691-6

Belles-Sampera, J., Merigó, J.M., Guillén, M., Santolino, M., 2014. Indicators for the characterization of discrete Choquet integrals. Inf. Sci. (Ny). 267, 201–216. https://doi.org/10.1016/J.INS.2014.01.047

Bergmeir, C., Hyndman, R.J., Benítez, J.M., 2016. Bagging exponential smoothing methods using STL decomposition and Box–Cox transformation. Int. J. Forecast. 32, 303–312. https://doi.org/10.1016/J.IJFORECAST.2015.07.002

Bicego, M., Baldo, S., 2016. Properties of the Box–Cox transformation for pattern classification. Neurocomputing 218, 390–400. https://doi.org/10.1016/J.NEUCOM.2016.08.081

Biswas, P., Pramanik, S., Giri, B.C., 2016. TOPSIS method for multi-attribute group decision-making under single-valued neutrosophic environment. Neural Comput. Appl. 27, 727–737. https://doi.org/10.1007/s00521-015-1891-2

Box, G.E.., Cox, D.R., 1964. An analysis of transformations. J. R. Stat. Soc. Ser. B 211–252.

Chaji, A., Fukuyama, H., Khanjani Shiraz, R., 2018. Selecting a model for generating OWA operator weights in MAGDM problems by maximum entropy membership function. Comput. Ind. Eng. 124, 370–378. https://doi.org/10.1016/J.CIE.2018.07.040

Dujmović, J., Cordeliers, L., 2006. A comparison of andness/orness indicators, in: Proceedings of the 11th Information Processing and Management of Uncertainty International Conference (IPMU 2006).

Fernandez, M., Bucaram, S., Renteria, W., 2017. (Non-) robustness of vulnerability assessments to climate change: An application to New Zealand. J. Environ. Manage. 203, 400–412. https://doi.org/10.1016/j.jenvman.2017.07.054

Frigerio, I., De Amicis, M., 2016. Mapping social vulnerability to natural hazards in Italy: A suitable tool for risk mitigation strategies. Environ. Sci. Policy 63, 187–196. https://doi.org/10.1016/J.ENVSCI.2016.06.001

Fullér, R., 1996. OWA operators in decision making, in: Exploring the Limits of Support Systems. pp. 85–104.

Greco, S., Ishizaka, A., Tasiou, M., Torrisi, G., 2018. On the Methodological Framework of Composite Indices: A Review of the Issues of Weighting, Aggregation, and Robustness. Soc. Indic. Res. 1–34. https://doi.org/10.1007/s11205-017-1832-9

Hagenlocher, M., Hölbling, D., Kienberger, S., Vanhuysse, S., Zeil, P., 2016. Spatial assessment of social vulnerability in the context of landmines and explosive remnants of war in Battambang province, Cambodia. Int. J. Disaster Risk Reduct. 15, 148–161. https://doi.org/10.1016/J.IJDRR.2015.11.003

Han, A.K., 1987. A non-parametric analysis of transformations. J. Econom. 35, 191–209. https://doi.org/10.1016/0304-4076(87)90023-6

Hebbali, A., 2018. olsrr: Tools for Building OLS Regression Models. R Packag. version 0.5.1.

Jin, L., Kalina, M., Qian, G., 2017. Discrete and continuous recursive forms of OWA operators. Fuzzy Sets Syst. 308, 106–122. https://doi.org/10.1016/J.FSS.2016.04.017

KC, B., Shepherd, J.M., Gaither, C.J., 2015. Climate change vulnerability assessment in Georgia. Appl. Geogr. 62, 62–74. https://doi.org/10.1016/j.apgeog.2015.04.007

Lai, D., 2010. Box–Cox transformation for spatial linear models: a study on lattice data. Stat. Pap. 51, 853–864. https://doi.org/10.1007/s00362-008-0178-4

Langhans, S.D., Reichert, P., Schuwirth, N., 2014. The method matters: A guide for indicator aggregation in ecological assessments. Ecol. Indic. 45, 494–507. https://doi.org/10.1016/J.ECOLIND.2014.05.014

Liu, Y., Zhou, J., Chen, Y., 2014. Using fuzzy non-linear regression to identify the degree of compensation among customer requirements in QFD. Neurocomputing 142, 115–124. https://doi.org/10.1016/J.NEUCOM.2014.01.053

Lucarelli, C., Mazziotta, M., Talucci, V., Ungaro, P., 2014. Composite Index for Measuring Italian Regions’ Environmental Quality Over Time, in: METMA VII and GRASPA14 Conference. Torino.

Maggino, F., 2017. Complexity in society: from indicators construction to their synthesis. Springer International Publishing. https://doi.org/10.1007/978-3-319-60595-1

Makoka, D., 2008. Risk, Risk Management and Vulnerability to Poverty in Rural Malawi. Cuvillier Verlag.

Mazziotta, M., Pareto, A., 2014. A COMPOSITE INDEX FOR MEASURING ITALIAN REGIONS’DEVELOPMENT OVER TIME. Riv. Ital. di Econ. Demogr. e Stat. 68.

Mysiak, J., Torresan, S., Bosello, F., Mistry, M., Amadio, M., Marzi, S., Furlan, E., Sperotto, A., 2018. Climate risk index for Italy. Philos. Trans. A. Math. Phys. Eng. Sci. 376, 20170305. https://doi.org/10.1098/rsta.2017.0305

NIST/SEMATECH, 2013. E-Handbook of Statistical Methods [WWW Document]. Natl. Inst. Stand. Technol. (NIST), United States Dep. Commer. URL https://www.itl.nist.gov/div898/handbook/eda/section3/eda336.htm (accessed 9.5.18).

OECD, 2008. Handbook on constructing composite indicators. OECD Publ.

Pinar, M., Cruciani, C., Giove, S., Sostero, M., 2014. Constructing the FEEM sustainability index: A Choquet integral application. Ecol. Indic. 39, 189–202. https://doi.org/10.1016/J.ECOLIND.2013.12.012

Pohlert, T., 2017. ppcc: Probability Plot Correlation Coefficient Test. R Packag. version 1.0.

Proietti, T., Lütkepohl, H., 2013. Does the Box–Cox transformation help in forecasting macroeconomic time series? Int. J. Forecast. 29, 88–99. https://doi.org/10.1016/J.IJFORECAST.2012.06.001

Saisana, M., Domínguez-Torreiro, M., Vértesy, D., 2018. Joint Research Centre Statistical Audit of the 2018 Gobal Innovation Index, in: Global Innovation Index 2018. Ithaca, Fontainebleau, and Genev, pp. 71–88.

Sakia, R.M., 1992. The Box-Cox transformation technique: a review. Stat. 41, 169–178.

Shanian, A., Savadogo, O., 2006. TOPSIS multiple-criteria decision support analysis for material selection of metallic bipolar plates for polymer electrolyte fuel cell. J. Power Sources 159, 1095–1104. https://doi.org/10.1016/J.JPOWSOUR.2005.12.092

Yager, R., 1988. On ordered weighted averaging aggregation operators in multicriteria decisionmaking. IEEE Trans. Syst. Man. Cybern. 18, 183–190.

Zabeo, A., 2011. A decision support system for the assessment and management of surface waters. Ca’Foscari University of Venice.
