# Supplementary material for: Constructing a comprehensive disaster resilience index: The case of Italy
Source: PLoS One. 2019 Sep 16;14(9):e0221585. doi: 10.1371/journal.pone.0221585 (PMC6746365; doi:10.1371/journal.pone.0221585)
Supplement: S4 Appendix — (DOCX) [file pone.0221585.s004.docx]

**S4 Appendix. Results**

## 1. Resilience at municipal scale

In order to investigate the correlations between CDRI and each variable, the Pearson correlation coefficient has been calculated (De Groeve et al., 2015; OECD, 2008). The strength of correlations is in the range of “very weak” to “moderate” - classes defined by (Bendanillo et al., 2016) – and mostly statistically significant (*p* < 0.001) (Table A). Therefore, the results are not significantly biased toward any variable, as the correlations suggest.

**Table A. Pearson correlation between CDRI and considered variables**

| Correlation | |  |  |  |  |
| --- | --- | --- | --- | --- | --- |
| Indicator | Pearson Correlation | Sig. (2-tailed) | Indicator | Pearson Correlation | Sig. (2-tailed) |
| ACC1_D | 0.211 | 0 | COH_10_t | -0.158 | 0 |
| ACC2_D | 0.178 | 0 | COH_11 | 0.425 | 0 |
| HC_1_t | -0.05 | 0 | EDU_1_t | 0.429 | 0 |
| HC_2_t | -0.004 | 0.688 | EDU_2 | 0.144 | 0 |
| HC_3_t | 0.256 | 0 | EDU_3 | 0.359 | 0 |
| HC_4 | 0.153 | 0 | INS_1 | 0.427 | 0 |
| COH_1_t | 0.011 | 0.318 | ENV_1 | 0.465 | 0 |
| COH_2 | 0.231 | 0 | ENV_2_t | 0.423 | 0 |
| COH_3 | -0.076 | 0 | RE_1 | 0.494 | 0 |
| COH_4_t | 0.014 | 0.216 | RE_2 | -0.354 | 0 |
| COH_6 | 0.117 | 0 | RE_3 | 0.453 | 0 |
| COH_7 | 0.148 | 0 | RE_4_t | -0.133 | 0 |
| COH_8 | -0.206 | 0 | RE_5_t | 0.416 | 0 |

## 2. Sensitivity analysis

A full set of the cross-sections from various normalizations are provided in the following graphs.

**Figure A. Section of OWA scores derived from Topsis normalized data for different combination of weights for all the municipalities (linear and uniform)**

| 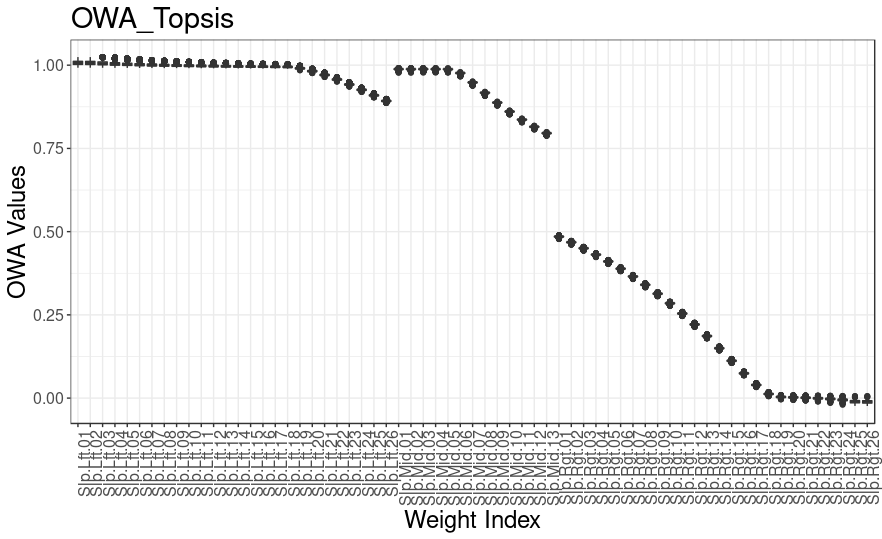 |
| --- |
| 1. Linear |
| 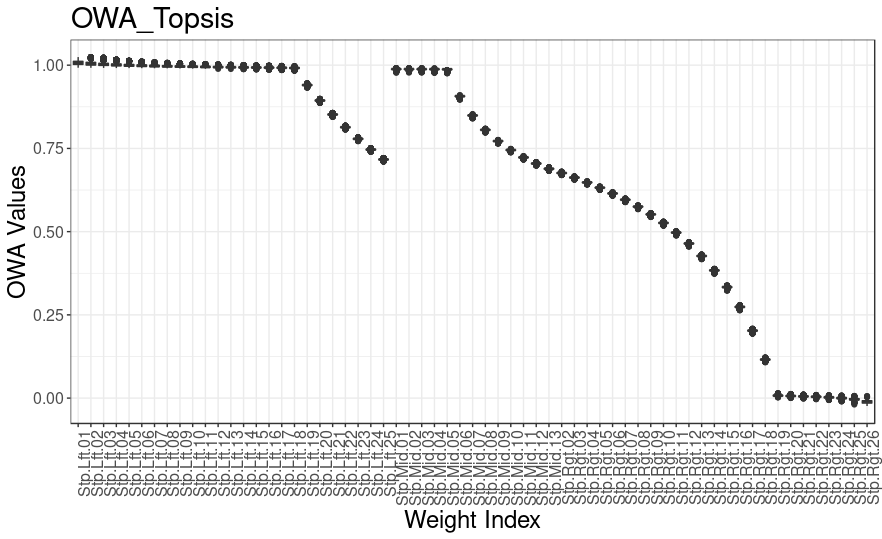 |
| 1. Uniform |

**Figure B. Section of OWA scores derived from Zscore normalized data for different combination of weights for all the municipalities (linear and uniform)**

| 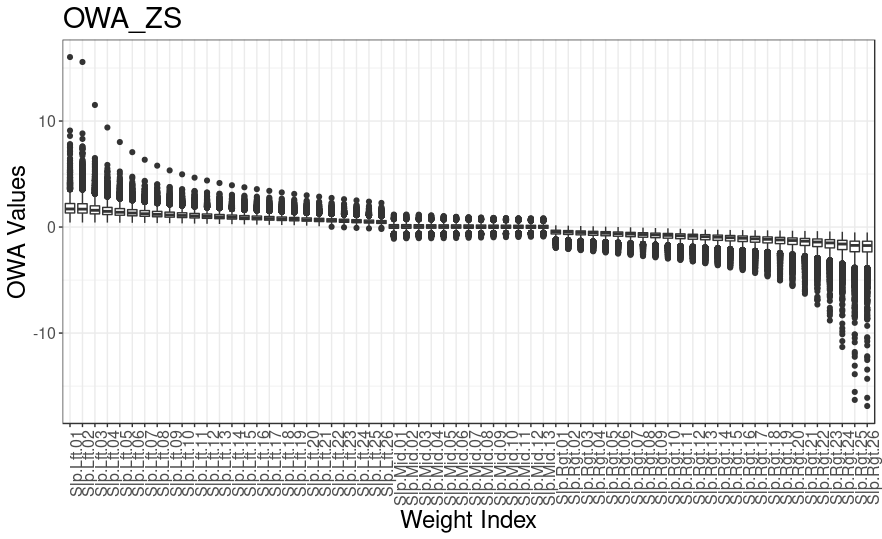 |
| --- |
| a) Linear |
| 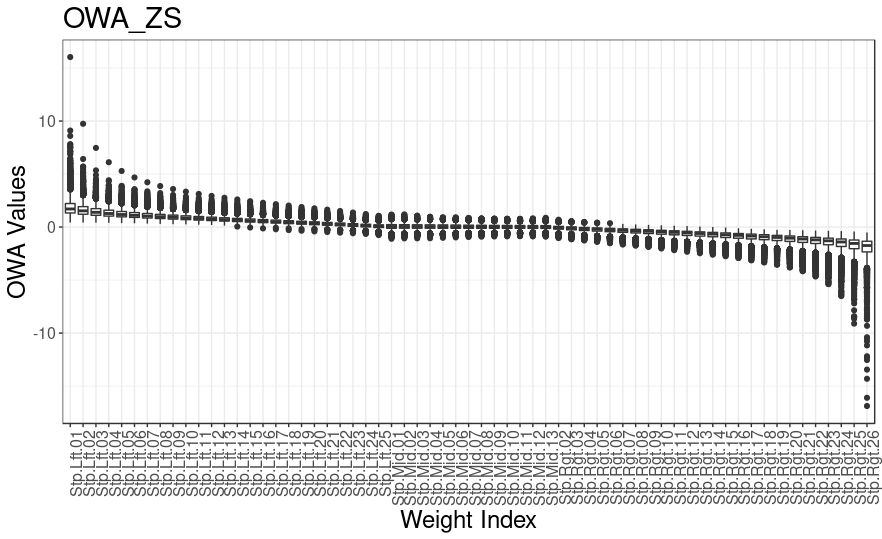 |
| b) Uniform |

**Figure C. Section of OWA scores derived from AMP normalized data (with no transformation) for different combination of weights for all the municipalities (linear and uniform)**

| 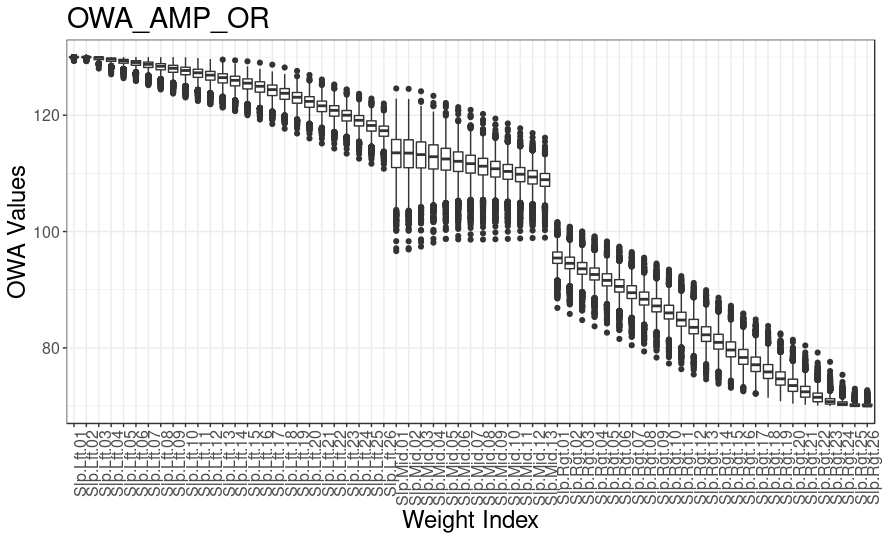 |
| --- |
| a) Linear |
| 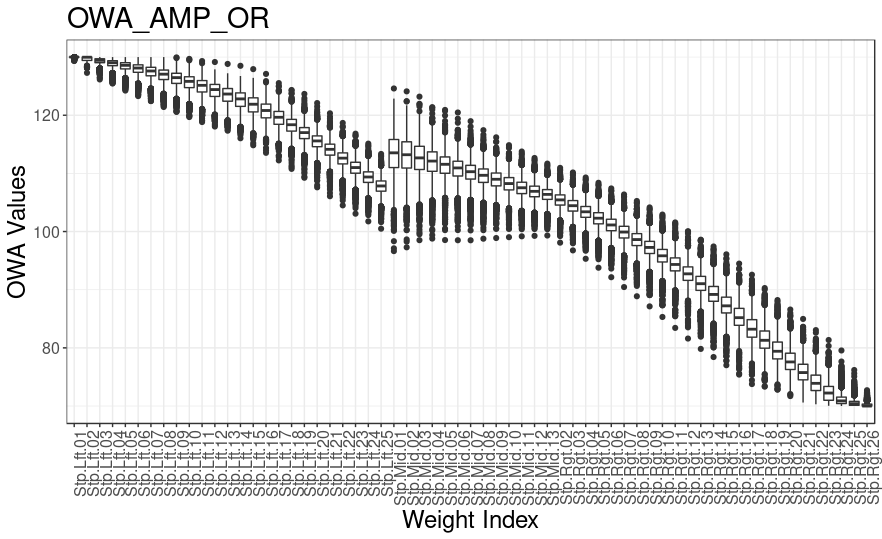 |
| b) Uniform |

**References**

Bendanillo, F.E., Yurong, R.R., Roble, N.D., Yee, J.C., Sotto, F.B., 2016. Species Composition, Abundance and Distribution of Seawater Bugs (Order Hemiptera: Class Insecta) in Badian, Cebu, Philippines. J. Aquat. Sci. Vol. 4, 2016, Pages 1-10 4, 1–10. https://doi.org/10.12691/JAS-4-1-1

De Groeve, T., Poljansek, K., Vernaccini, L., 2015. Index for Risk Management - INFORM. JRC Sci. Policy Reports - Eur. Comm. 96.

OECD, 2008. Handbook on constructing composite indicators. OECD Publ.
