# Supplementary material for: Constructing a comprehensive disaster resilience index: The case of Italy
Source: PLoS One. 2019 Sep 16;14(9):e0221585. doi: 10.1371/journal.pone.0221585 (PMC6746365; doi:10.1371/journal.pone.0221585)
Supplement: S5 Appendix — (DOCX) [file pone.0221585.s005.docx]

**S5 Appendix. Logic Scoring of Preference (LSP)**

The Logic Scoring of Preference (LSP) is a multi-criteria evaluation (MCE) method based on soft computing concepts of generalized conjunction/disjunction (Dujmovi’c, 1996; Dujmović et al., 2008; Montgomery et al., 2016). The main advantages of LSP method are i) the consistency with human evaluation reasoning in the case of non-linear aggregation criteria based on various compensation degrees and ii) capacity to incorporate large number of inputs while maintaining the importance of each input throughout the multicriteria evaluation (Montgomery et al., 2016). The LSP method is composed of the following three main steps:

*1) Developing a decision tree:* In the first stage, a decomposition structure of the evaluated object has to be designed. Figure A illustrates the decomposition structure of the CDRI used in the analysis. Since there are some calculation limitations in aggregating more than five variables, the criteria have been classified into two dimensions namely resources and social characteristics for final computations.


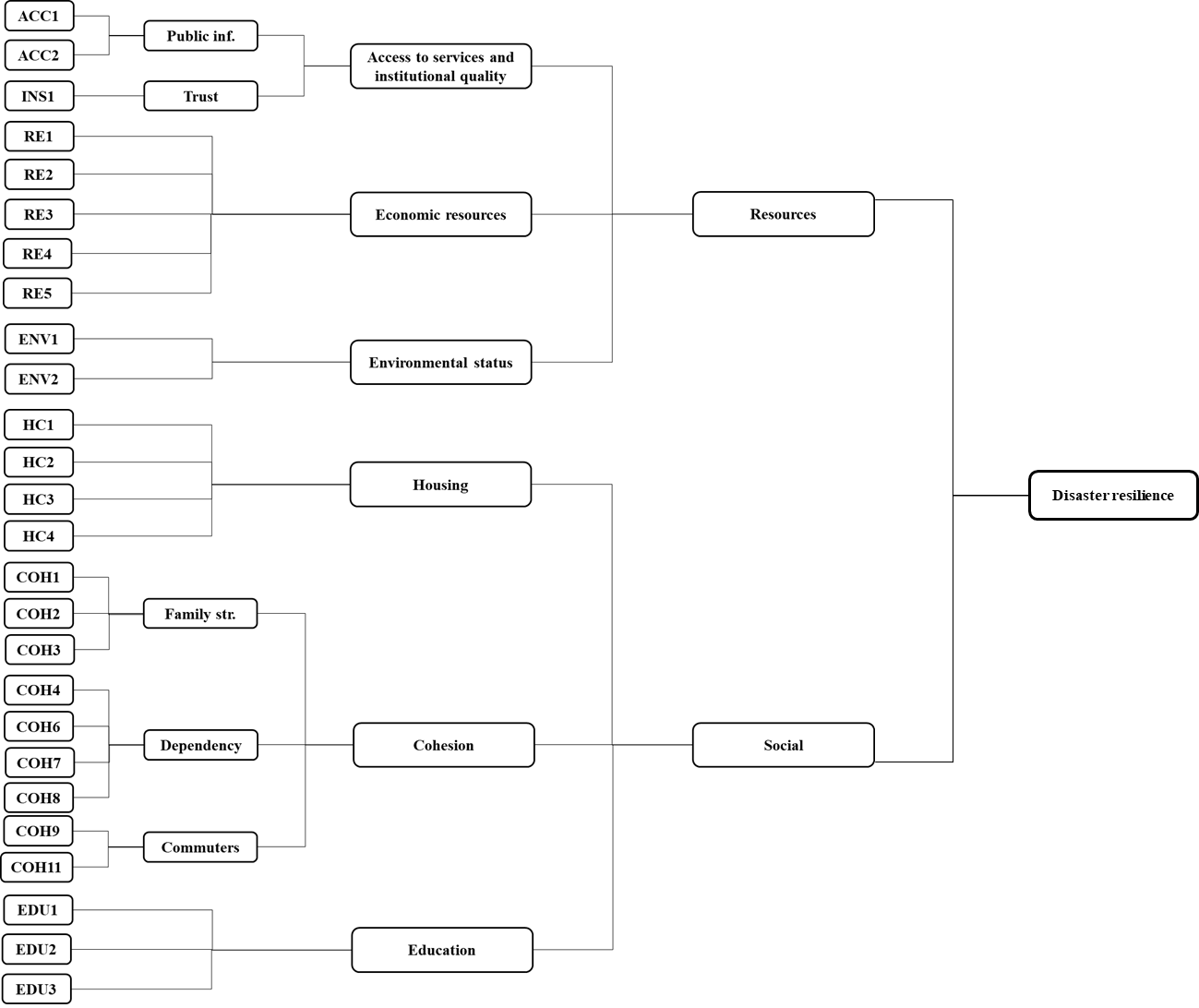


**Figure A. LSP decomposition structure of the CDRI**

*2) Defining the elementary attribute criteria:* in this stage, transformed attributes (indicators) have been normalized using Adjusted Mazziotta-Pareto (AMP). The AMP normalization is given by:

$r_{ij}=\frac{{(x}_{ij}-{Min}_{xj})}{{(Max}_{xj}-{Min}_{xj})}60+70$

where $x_{ij}$ is the value of the indicator j for the municipality *i* and ${Min}_{xj}$ and ${Max}_{xj}$ are the goalposts for the indicator j. To revert the scales of indicators with negative polarity, the complement of the equation with respect to 200 has been calculated (Lucarelli et al., 2014; Mazziotta and Pareto, 2014)

*3) Developing the aggregation structure:* In the next stage, the normalized attributes are aggregated using a power mean andor function ($M_{p}$) with various compensation degrees taking into account speciﬁc stakeholder’s goals and requirements. Those requirements affect the selection of logic aggregation operators, and the relative importance of individual attributes and their groups.

$M_{p}\left( x_{1},\ldots,x_{n} \right)={(\frac{1}{n}\sum_{i=1}^{n} x_{i}^{r})}^{\frac{1}{r}}$

In our study, we have replicated the aggregation at the nodes of the attribute tree using various LSP andor configurations (Table A) to analyse the sensitivity and robustness of the outcomes (Dujmovi’c, 1996; Dujmović et al., 2008; Fernandez et al., 2017; Montgomery et al., 2016). Table A contains the LSP andor parameters *r* corresponding to different conjunction/disjunction levels among attributes. The *r* values for two, three, four and five inputs have been extracted from Dujmovi’c (1996).

**Table A. Symbols and parameters of the LSP andor function**

| Operation | Symbol | r2 | r3 | r4 | r5 |
| --- | --- | --- | --- | --- | --- |
| Disjunction | D | max | max | max | max |
| Strong QD (+) | D++ | 20.63 | 24.3 | 27.11 | 30.09 |
| Strong QD | D+ | 9.521 | 11.095 | 12.27 | 13.235 |
| Strong QD (-) | D+- | 5.802 | 6.675 | 7.316 | 7.819 |
| Medium QD | DA | 3.929 | 4.45 | 4.825 | 5.111 |
| Weak QD (+) | D-+ | 2.792 | 3.101 | 3.318 | 3.479 |
| Weak QD | D- | 2.018 | 2.187 | 2.302 | 2.384 |
| Square Mean | SQU | 2 | 2 | 2 | 2 |
| Weak QD (-) | D-- | 1.449 | 1.519 | 1.565 | 1.596 |
| Arithmetic Mean | A | 1 | 1 | 1 | 1 |
| Weak QC (-) | C-- | 0.619 | 0.573 | 0.546 | 0.526 |
| Weak QC | C- | 0.261 | 0.192 | 0.153 | 0.129 |
| Geometric Mean | GEO | 0 | 0 | 0 | 0 |
| Weak QC (+) | C-+ | -0.148 | -0.208 | -0.235 | -0.251 |
| Medium QC | CA | -0.72 | -0.732 | -0.721 | -0.707 |
| Harmonic Mean | HAR | -1 | -1 | -1 | -1 |
| Strong QC (-) | C+- | -1.655 | -1.55 | -1.455 | -1.38 |
| Strong QC | C+ | -3.51 | -3.114 | -2.823 | -2.606 |
| Strong QC (+) | C++ | -9.06 | -7.639 | -6.689 | -6.013 |
| Conjunction | C | min | min | min | min |

Figure B illustrates the LSP scores for all municipalities derived from andor parameters shown in Table A. As expected, the aggregated values follow a decreasing trend while moving from strong disjunction to strong conjunction assumptions (extremes for min and max operators).

**
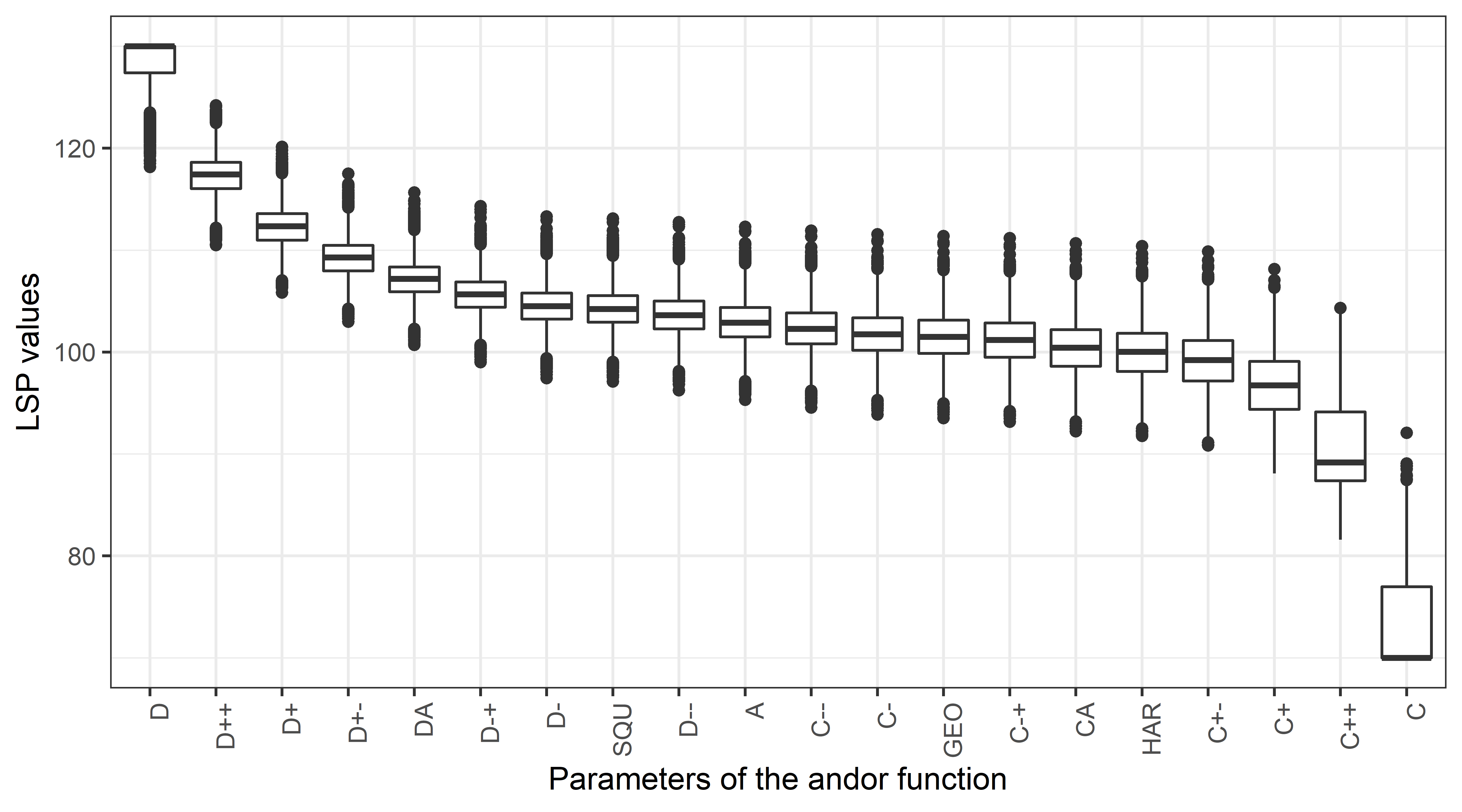
**

**Figure B. LSP scores derived from various andor parameters for all the municipalities**

In order to provide robust final scores for municipalities considering all the andor simulated configurations, relative dominance measure has been computed. The results of dominance analysis can be used to identify relative resilience measures across the municipalities. Figure C illustrates standardized relative dominance scores for Italian municipalities considering the overall variability imposed by diverse LSP configurations.

**
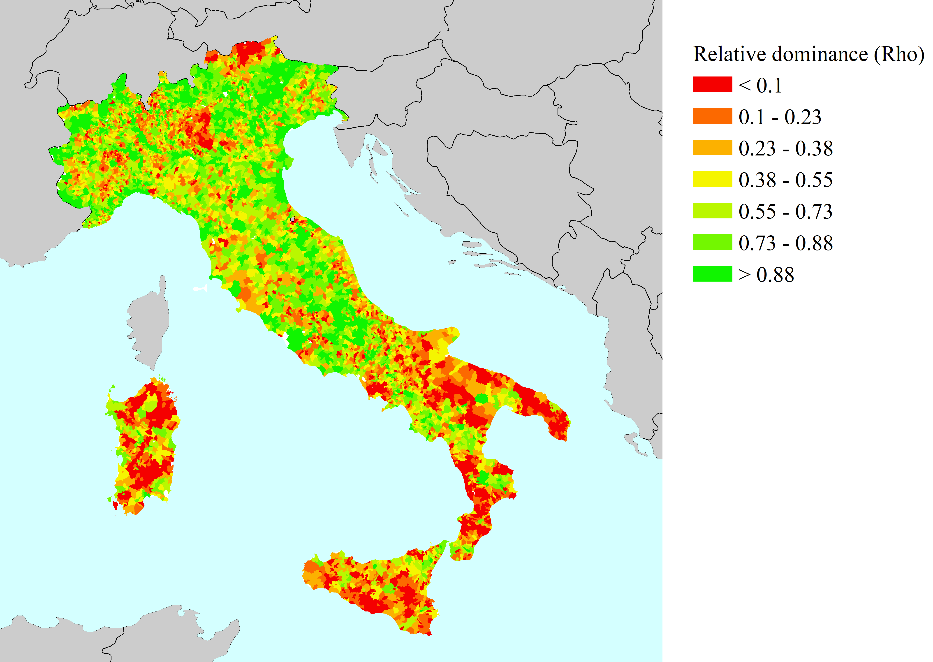
**

**Figure C. Relative dominance scores derived from various andor configurations.**

Figure D shows a comparison between the dominance measures obtained from LSP and OWA robustness simulations. Both results show approximately analogous trends across municipalities. Nevertheless, the one derived from OWA simulations displays higher deviations from the mean values specially for municipalities subjected to below-average performance in terms of disaster resilience. The difference may caused by i) the number of models simulated for each analysis, ii) the type of normalizations used. For instance, non-linear Topsis normalization behaves as a low pass filter, and the slope drastically changes after a threshold located in the high Andness tail of the OWA weights distribution with low compensation levels (Figure 9-c in manuscript). This behavior results in lower scores for below-average performance municipalities in compare to results obtained by other normalizations and consequently drags down the dominance measures for those municipalities.

| **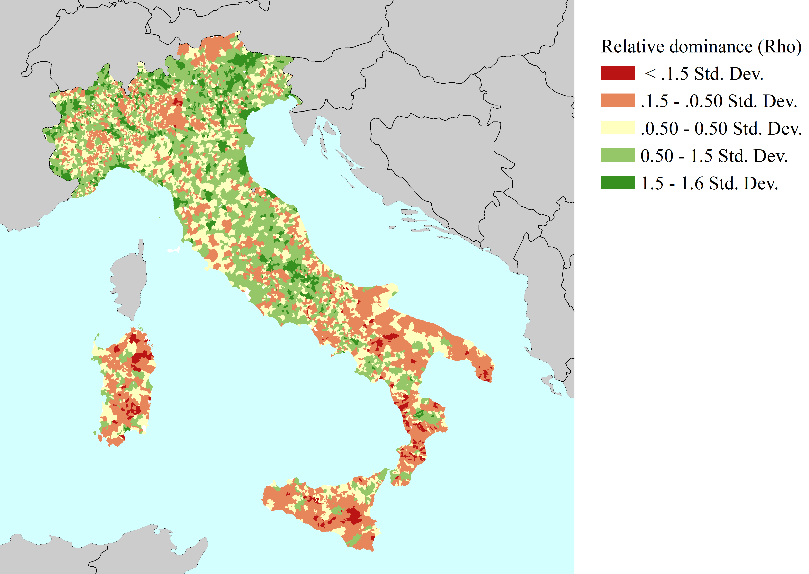** | **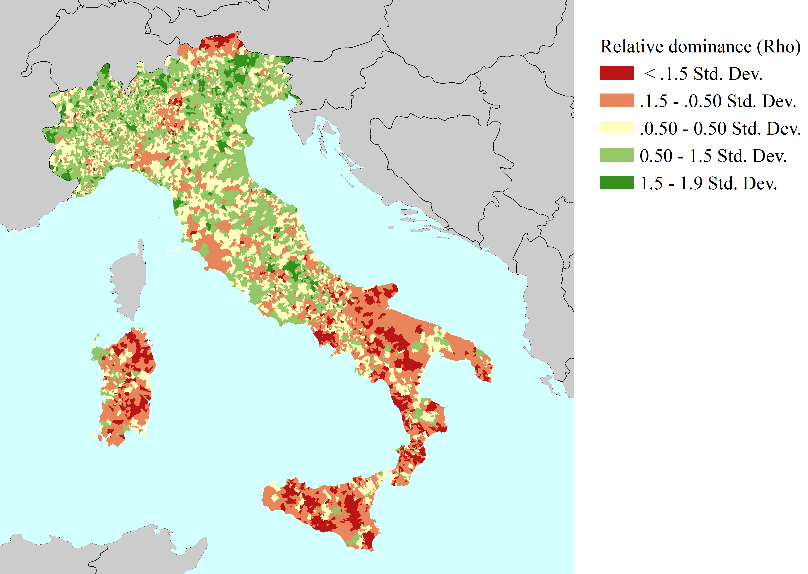** | **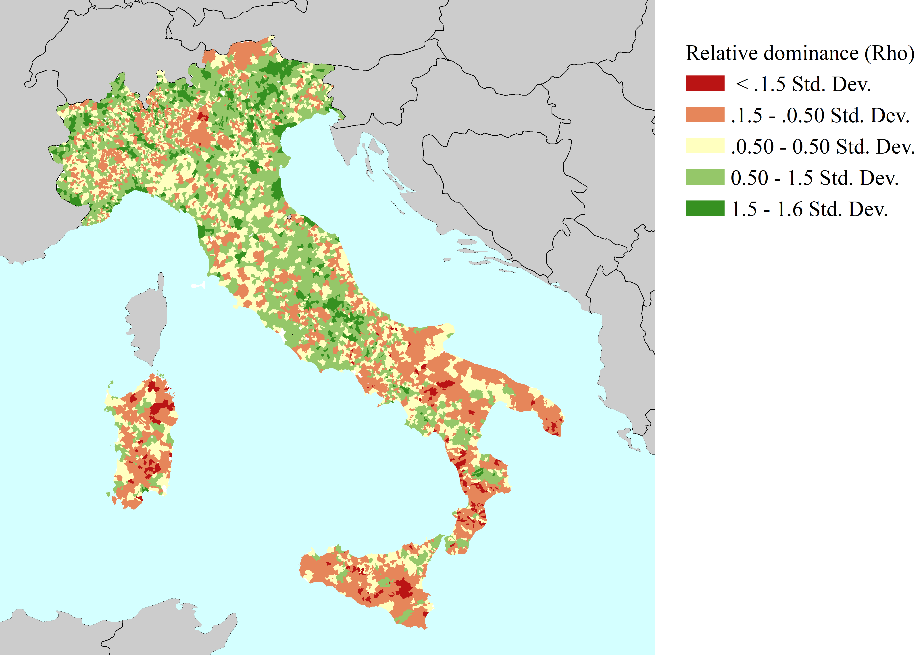** |
| --- | --- | --- |
| a) LSP | b) OWA |  |

**Figure D. Comparisons between CDRI relative dominance measures derived from LSP and OWA configurations.**

**References**

Dujmovi’c, J., 1996. A Method For Evaluation And Selection Of Complex Hardware And Software Systems. C. 96 Proc. 1, 368–378.

Dujmović, J.J., Ralph, J.W., Dorfman, L.J., 2008. Evaluation of disease severity and patient disability using the lsp method. Proc. 12th Inf. Process. Manag. Uncertain. Int. Conf. (IPMU 2008) 1398–1405.

Fernandez, M., Bucaram, S., Renteria, W., 2017. (Non-) robustness of vulnerability assessments to climate change: An application to New Zealand. J. Environ. Manage. 203, 400–412. https://doi.org/10.1016/j.jenvman.2017.07.054

Lucarelli, C., Mazziotta, M., Talucci, V., Ungaro, P., 2014. Composite Index for Measuring Italian Regions’ Environmental Quality Over Time, in: METMA VII and GRASPA14 Conference. Torino.

Mazziotta, M., Pareto, A., 2014. A COMPOSITE INDEX FOR MEASURING ITALIAN REGIONS’DEVELOPMENT OVER TIME. Riv. Ital. di Econ. Demogr. e Stat. 68.

Montgomery, B., Dragićević, S., Dujmović, J., Schmidt, M., 2016. A GIS-based Logic Scoring of Preference method for evaluation of land capability and suitability for agriculture. Comput. Electron. Agric. 124, 340–353. https://doi.org/10.1016/J.COMPAG.2016.04.013
