# Supplementary material for: Constructing a comprehensive disaster resilience index: The case of Italy
Source: PLoS One. 2019 Sep 16;14(9):e0221585. doi: 10.1371/journal.pone.0221585 (PMC6746365; doi:10.1371/journal.pone.0221585)
Supplement: S6 Appendix — (DOCX) [file pone.0221585.s006.docx]

**S6 Appendix. Comparison between original and population corrected form of distance decay indicators and final indices.**

We have used the distance decay indicators in the current form (excluding the underlying population size) to replicate the classification of the disadvantaged (inner) areas as currently used in the “Italian National Strategy for Inner Areas (NSIA)” elaborated in National Plan of Reform. The aforesaid strategy is one of the most comprehensive and integrated strategies for tackling the problems of depopulation and low access to services in Europe. Accordingly, inner areas are the ones with low population density, characterised by their distance from the main service centres (education, health and mobility), make up 53 % of Italian municipalities (4 261), are home to 23 % of the Italian population (13 540 000 inhabitants) and cover 60 % of the national territory. Although within the definition of inner areas there are also marked differences (e.g. demographics), the distance-decay measures are the priority focus for policy interventions (Barca et al., 2014; ENRD, 2018). We have applied the same logic to assess disadvantaged municipalities in terms of fire and rescue units as the matter of consistency.

In this Annex, we investigate the sensitivity of the distance-decay indicators and final index to underlying population size. To do so, the normalized distance indicators have been multiplied by a population correction coefficient. In the case of the “travel distance to service centers”, the population of the inner municipalities (excluding service centers) were normalized in the range of [0.1,0.99] to avoid zero values using the following formula:

$NP_{i}=\frac{{(x}_{i}-{Min}_{x})}{{(Max}_{x}-{Min}_{x})}0.89+0.1$ (Equation A)

where $x_{i}$ is the population for the municipality *i* and ${Min}_{x}$ and ${Max}_{x}$ are the goalposts for the population values. In the case of the “travel distance to fire brigades”, the population of the municipalities without fire brigades were normalized in the range of [o.1,0.99] using Equation A. The standard deviation visualization method has been used to better show the relative differences.

**A) Travel distance to Service centers**

Figure A shows the standardized values for the original and population equalized distance to service center’ indicator. Minor changes can be observed specially in Sicilia region due to higher population coefficient values.

| 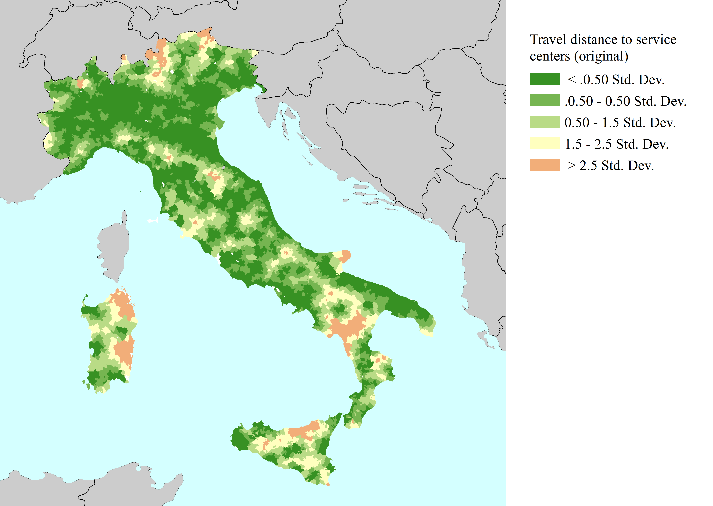 | 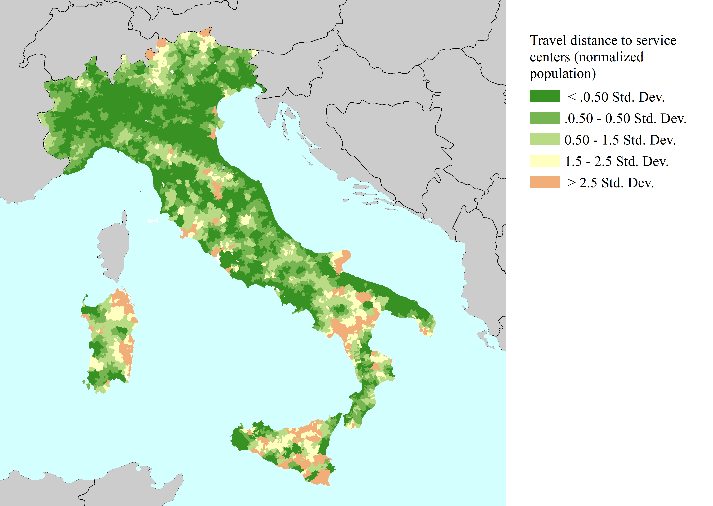 |
| --- | --- |
| a) Travel distance to service centers (original data) | b) Travel distance to service centers (normalized population) |

**Figure A. Travel distance to service centers – original and population corrected.**

**B) Travel distance to fire brigades**

Figure B shows the standardized values for the original and population equalized distance to fire brigades’ indicator. The values multiplied by population coefficient show higher standard deviation range in compare to original one (2.5 std vs 1.5 std) which indicates higher variance in the case of remotest areas.

| 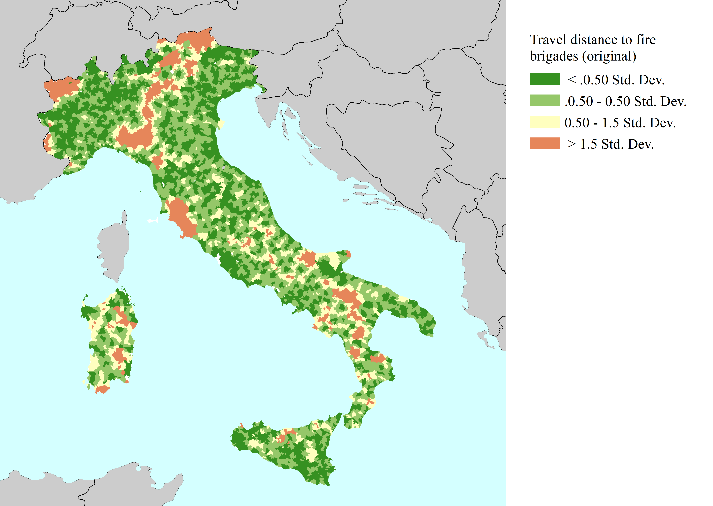 | 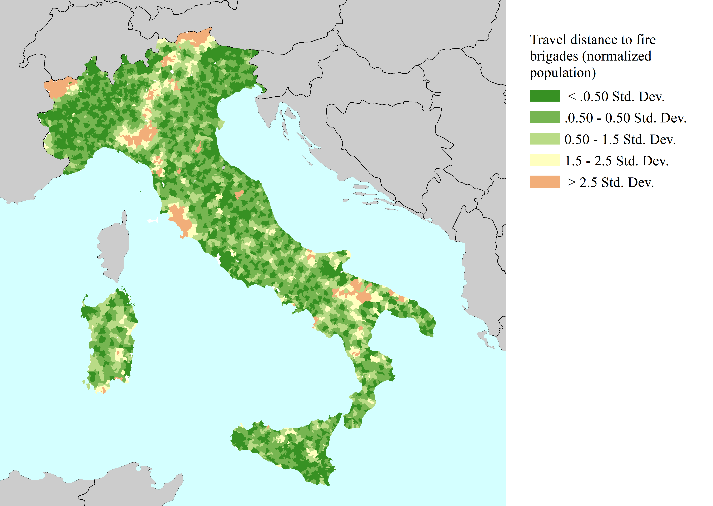 |
| --- | --- |
| a) Travel distance to fire brigades (original data) | b) Travel distance to fire brigades (normalized population) |

**Figure B. Travel distance to fire brigades – original and population corrected.**

**C) Comparison between original and new CDRI rankings**

The results show minor differences in terms of rank reversals. Although the standard deviation is slightly smoothened in compare to the original index, the relative resilience measure among the units and relative dispersions are quite unchanged. This can be explained by means of the Inner Areas’ definition in Italy in which the demographics (in the form of population density) has been already elaborated.

| 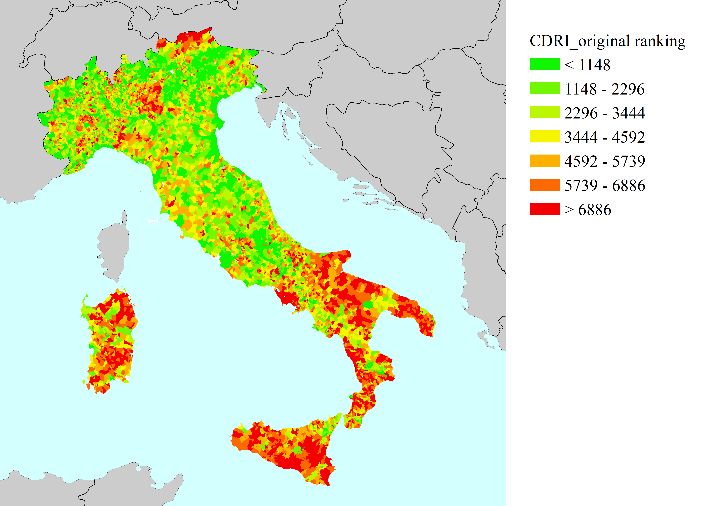 | 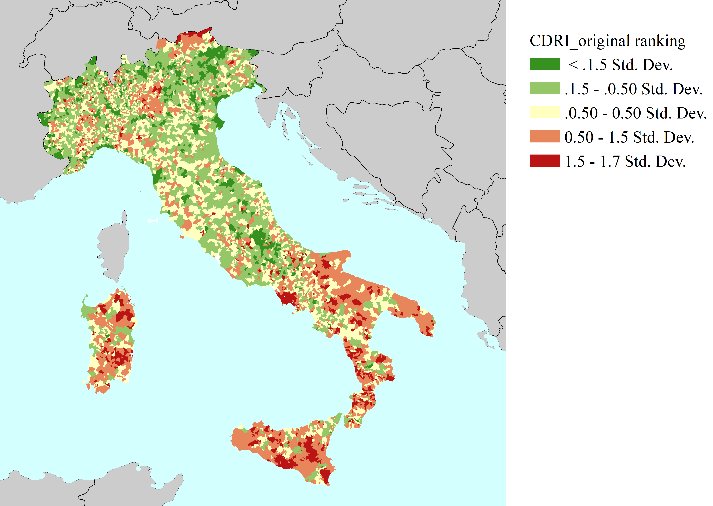 |
| --- | --- |
| a) Original CDRI | |
| 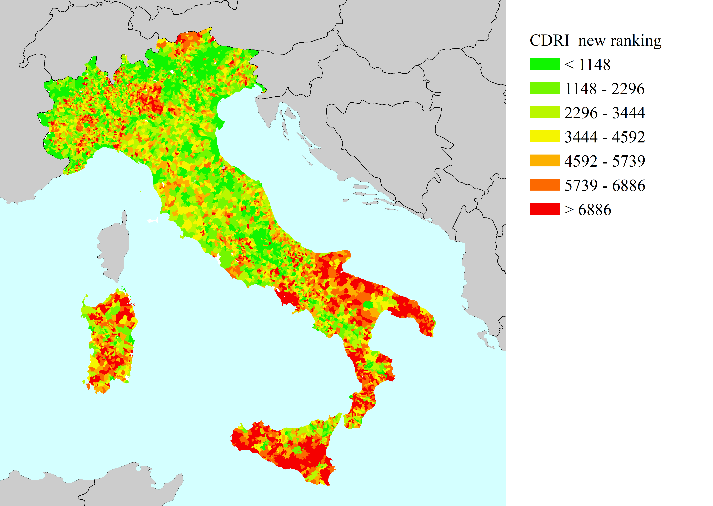 | 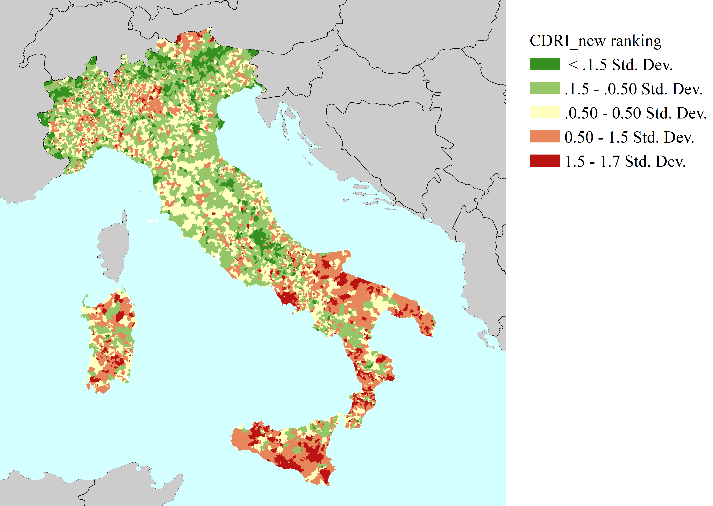 |
| b) New CDRI | |

**Figure C. Comparison between original and new CDRI rankings**
